# Active heterogeneous mode coupling in bi-level multi-physically architected metamaterials for temporal, on-demand and tunable programming

**DOI:** 10.1038/s44172-025-00420-7

**Published:** 2025-06-07

**Authors:** S. Mondal, T. Mukhopadhyay, S. Naskar

**Affiliations:** Faculty of Engineering and Physical Sciences, University of Southampton, Southampton, UK

**Keywords:** Aerospace engineering, Mechanical engineering

## Abstract

Traditionally materials show an uncoupled response between normal and shear modes of deformation. Here we propose to achieve heterogeneous mode coupling among the normal and shear modes, but in conventional symmetric lattice geometries through intuitively mounting electro-active elements. The proposed bi-level multi-physically architected metamaterials lead to an unprecedented programmable voltage-dependent normal–shear constitutive mode coupling and active multi-modal stiffness modulation capability for critically exploitable periodic or aperiodic, on-demand and temporally tunable mechanical responses. Further, active partial cloaking concerning the effect of far-field complex stresses can be achieved, leading to the prospect of averting a range of failure and serviceability conditions. The tunable heterogeneous mode coupling in the new class of symmetric metamaterials would lead to real-time control of mechanical responses for temporal programming in a wide range of advanced mechanical applications, including morphing and transformable geometries, locomotion in soft robotics, embedded actuators, enhanced multi-modal energy harvesting, vibration and wave propagation control.

## Abstract (web summary)

A class of bi-level architected piezoelectric lattice metamaterials is introduced that is capable of tunably coupling shear and axial deformation modes. This strategy could allow facile control of active shape morphing for robotic locomotion.

## Introduction

Artificially engineered multi-physical lattice-based metamaterials have started receiving tremendous attention over the last couple of years due to their ability to modulate physical properties and shapes actively even after manufacturing. The term multi-functional is used often in this context for referring to a range of material characteristics that are counterintuitive and unusual for realizing simultaneously compared to those of any typical naturally occurring materials^1,2^. Some examples of such characteristics include ultra-lightweightness with high specific stiffness, property modulation, zero or negative elastic moduli and Poisson’s ratios, vanishing shear modulus, negative mass density, electromagnetic and mechanical cloaking, tunable wave propagation characteristics, and their on-demand programmability^3–8^. Here the term lattice indicates 2D and 3D repetitive-unit cells (representative volume element) based design where each unit cell can have a regular shape, such as square, hexagonal, triangular, pentagon, etc., leading to a bending or stretching dominated behavior of the connecting beam-like straight or curved members. After tremendous progress in computationally conceptualizing and manufacturing lattice metamaterials with complex cell geometries over the last decade, a strong rationale has evolved lately to achieve active and on-demand property modulation in real-time with greater sensitivity. The central theme of this paper is to demonstrate active normal–shear mode coupling in regular symmetric 2D geometries through the introduction of bi-level rationally designed multi-physical architectures at the elementary beam-level that is also applicable to a wide range of other 2D and 3D lattices.

Initial works on mechanical metamaterials can be traced back to the research concerning effective elastic properties obtained through passive-type designs of lattice architectures, where the effective properties can be obtained as a function of unit cell geometries along with intrinsic material properties^9,10^. In this class of metamaterials, the normal and shear modes are typically decoupled, indicating that there would be normal deformation under far-field normal stresses and shear deformation under far-field shear stresses. Further, there would be no instance of normal and shear deformations simultaneously under the application of only one mode of stress or deformation. A large number of these metamaterials are developed on the traditional premise that once these are manufactured based on a unit cell geometry, the effective mechanical properties cannot be modulated further on an active and on-demand basis. The mechanical analyses in such lattice metamaterials involve evaluating the effective elastic properties, failure strength, energy absorption capacity and their simultaneous multi-objective modulation. In the current paper, we will focus on hexagonal honeycombs for demonstrating the active normal–shear mode coupling. Such hexagonal lattice geometries with efficient space-filling features are widely adopted in engineering applications and found in naturally occurring structural forms across the length scales^11^. Masters and Evans^12^ reported an analytical model for the prediction of elastic constants of hexagonal honeycombs considering flexural, stretching, and hinging of cell members. Wang and Stronge^13^ used a micropolar elasticity theory to derive the stiffness matrix for regular hexagonal honeycombs. Balawi and Abot^14^ reported a strain-energy-based refined analytic model for regular honeycombs taking curvature into consideration at each intersection point of the lattice. Xu et al.^15^ modified the traditional regular honeycomb into AuxHex structure (unit cells with both auxetic and hexagonal honeycomb patterns) in their study and derived the effective elastic moduli and plastic collapse stresses of the lattice. Mukhopadhyay et al.^16^ developed the theory for heterogeneous multi-material lattices, while Mukherjee and Adhikari^17^ further extended the theory to incorporate beam-level axial and shear deformations. Mukhopadhyay et al.^18^ derived the closed-form elastic moduli expressions of multi-material honeycomb lattices considering the effect of filler material. In the field of multi-material lattices, functionally graded metamaterials with longitudinal and thickness-wise beam-level gradation have been proposed for elasticity tailoring, failure mode manipulation (ductile and brittle) and applications in extreme surrounding environments (including hydrogen storage tank)^19,20^. Khalili and Alavi^21^ utilized the modified strain gradient theory to derive elastic moduli of the microcellular auxetic honeycomb lattices. Other directions of research in the field of cellular lattice metamaterials include nonlinear large deformation analysis^22^, the effect of residual and intrinsic stresses^23,24^, the effect of structural irregularity in the lattice geometry^11,25^, the influence of vibrating environment on the effective elastic moduli of lattices^26^, and single-curvature beam lattices^27^ and anti-curvature lattice designs^28,29^. Lately, the concept of inverse design and exploitation of machine learning algorithms have shown promising outcomes in developing novel metamaterial architectures^30–32^.

**Fig. 1:**
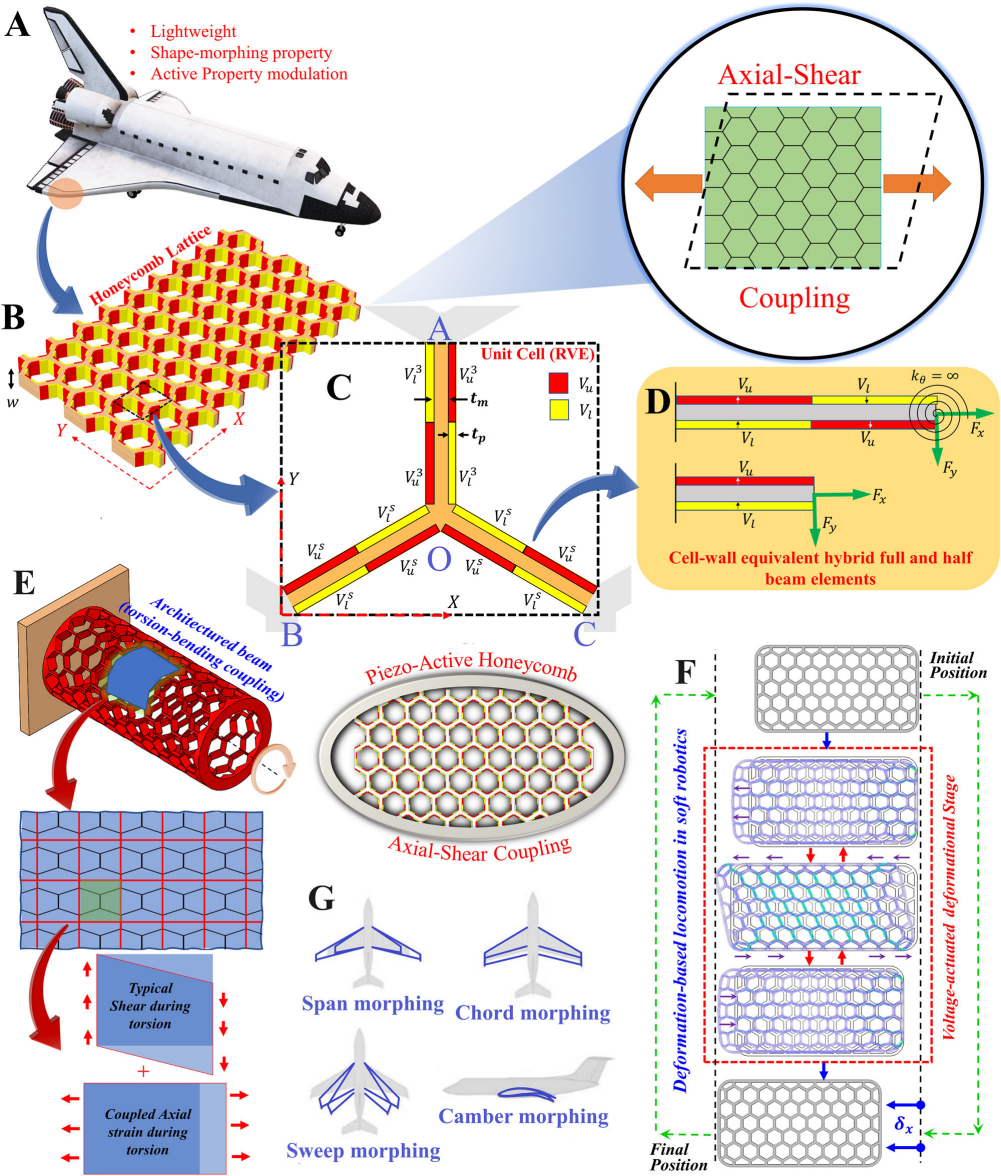
Bi-level architected lattice metamaterials with active mode coupling. **A** Prospective application of active lattices with mode coupling in adaptive wing morphing structures. **B** Active lattices with normal–shear mode coupling including visual representation through equivalent continua. **C** Representative volume element or unit cell for computing the homogenized mechanical behavior. Note that the lengths of upright (i.e. vertical) and slant members are taken as unequal here (*h* and *L* respectively), while the angle of the slant member BO with *X*-axis is *θ*. The joints in the unit cell (A, B, C and O) are indicated using blue color to differentiate these from sub-figure numbers. **D** Beam-level composite architecture with optimally placed active material (piezo) components. **E** Normal-shear mode coupling metamaterials resulting in tunable normal–torsion-bending coupling modes. **F** Prospective application of active mode coupling through periodic actuation for deformation-based locomotion in soft robotics. **G** Prospective exploitation of normal–shear mode coupling in aerodynamically adaptive wing morphing as aerofoil skin^53^. Note that the central theme of this paper is normal–shear coupling realized through piezo-active honeycombs as shown in the middle of the figure.

The literature reviewed in the preceding paragraph does not consider the notion of real-time active property modulation of effective mechanical properties. This implies it is impossible to modify or change the characteristics of the metamaterials once they are manufactured in order to suit specific applications or active operational demands. In this context, to possess on-demand property modulation capability, the metamaterial architecture should contain active elements in the geometry that can be activated based on thermoelasticity, magnetostriction, piezoelectricity, or other multiphysics-based phenomena^1^. The smart theories of such active components can be qualitatively appreciated through the typical expressions^33,34^: $\epsilon =s\sigma +dE,\epsilon =s\sigma +{d^{\prime}} H,$ and *ϵ* = *sσ*−*β*Δ*T*, where *σ* and *ϵ* are strain and stress; *E* and *H* are the applied electric and magnetic field; Δ is the applied temperature variation; *s*, *d*, ${d^{\prime}}$, *β* are elastic compliance, piezoelectric coefficient, magnetoelastic constant and thermal moduli tensor respectively. It can be noticed that the elastic deformations (strains) of such smart materials can be programmed by an externally applied electric field, magnetic field and temperature difference along with typical mechanical stresses which unfolds a de novo scope of exploiting their coupled physics along with metamaterials architectures to achieve on-demand property modulation. For instance, Sinha and Mukhopadhyay^35^ and Singh et al.^36^ proposed active honeycomb lattices made of magneto-active elements, wherein the effective stiffness can be modulated (contactless) as a function of magnetic field and mechanical stresses. Lim^37^ reported a temperature-adaptable Poisson’s ratio modulation in honeycombs with rectangular unit cells having rigid crossbeams (as upright members) and alternating bimetallic strips (as slant members) as the cell walls. In Wang and Liu’s work^38^, a similar unit cell structure is modified with piezoelectric elements where one of the strips of the bimetallic cell wall is replaced by a piezoelectric patch. The rigid crossbeam member is kept rigid and of a single material as before. They reported a half-beam-based analytical model to formulate its voltage-dependent effective Young’s moduli. The work of Wang and Liu^38^ is inspired by earlier research of Singh et al.^39,40^, where they reported a bottom-up unit cell-based analytical framework for active honeycombs using a modified piezo-stiffness matrix. Different multi-physical property modulation aspects were highlighted including the existence of negative Young’s modulus in the static scenarios, on-demand sign reversal of Poisson’s ratios, the effect of asymmetric piezo-placements on effective mechanical properties of honeycomb, etc. However, it has been observed experimentally and analytically that lattices with alternating bi-material piezoelectric elements give rise to higher piezoelectric sensitivity compared to conventional unimorph and bimorph configurations^41–43^. Motivated by these findings, in the present study we would propose a novel alternating tri-layered piezo-metallic strip-based bi-level (coupled design space at the beam level and the unit cell level) metamaterial architecture (including slant as well as upright members as depicted in Fig. 1A–D) with unique active capabilities, as discussed in the following sections. In the context of earlier works^39,40^, we introduce the placement of piezoelectric elements more effectively following a physics-informed beam-level architecture for achieving higher voltage sensitivity, and subsequently adding the piezoelectric elements to the vertical upright members leads to an unprecedented programmable mode coupling between the axial and shear deformations. The earlier works show that shear modulus cannot be modulated as a function of voltage. As an integral part of this study, we would show here that shear modulus can also be voltage-dependent along with Young’s moduli and Poisson’s ratios following the current active tri-member design. Further, from the analytical derivation viewpoint, we would introduce a direct and more accurate formulation from the very fundamental constitutive equation of piezoelectricity, rather than adopting an approximate equivalent moment and force-based approach used in the earlier works.

In the literature of metamaterials, symmetric unit cell geometries like hexagonal, rhombic, rectangular or triangular tessellations, while preferred for the ease of manufacturing, cannot lead to normal–shear mode coupling under applied unimodal strain or stress. So far, only a few special classes of lattice have been found to exhibit such mode coupling, albeit in a passive and non-programmable regime (i.e. the active property modulation is not achievable). For instance, 2D chiral cellular lattices, composed of arc-shaped, V-shaped, and semicircular-shaped cell walls^44,45^, have been investigated for their effective normal and coupled mechanical properties. Fleisch et al.^46^ reported an experimental study to investigate a normal–shear coupling effect in modified 2.5D and 3D chiral-based mechanical metamaterials. Mousanezhad et al.^47^ investigated the effective elastic properties of chiral and anti-chiral honeycombs based on an energy-based approach. A critical review of such literature concerning normal–shear mode coupling in lattice metamaterials reveals that besides complex unit cell geometries, the current state-of-the-art does not possess the capability of active and on-demand modulation of such heterogeneous mode coupling. The proposed bi-level multi-physically architected metamaterials would lead to an unprecedented programmable voltage-dependent normal–shear mode coupling for critically exploitable temporally periodic or aperiodic, on-demand and tunable mechanical responses. Further, as a derivative of such a metamaterial would lead to normal–torsional mode coupling as depicted in Fig. 1E. The active programmable modulation in the coupled responses, obtained through symmetric lattice geometries, would have crucial applications in soft robotics, aero-dynamically adaptive morphing aircraft wings and wind turbine blades, robotic control and gripper applications, MEMS devices (refer to Fig. 1F, G), and a range of other advanced engineering technologies.

## Methods

### Underlying concepts of the high-fidelity computational framework for actively coupled strain field

The multi-scale bottom-up framework for developing active metamaterials is illustrated in Fig. 1A–D, starting from beam-level architecture, unit cells and lattices to the level of structural application. We have considered a tri-membered unit cell, wherein the piezo patches are placed on the vertical and slant beam substrates as pairs for greater voltage sensitivity. Such beam-level architecture is rationalized through the physics-based insight that the bending moment at the center of each beam-like member becomes zero due to the periodic boundary condition of the unit cells, leading to an S-like beam-level deformed shape under the application of lattice-level far-field stresses. Thus, placement of the piezo patches in the current form would either tend to accentuate or dilute the S-like curvatures under far-field mechanical stresses, depending on the polarity of applied voltages. This would, in turn, control the lattice level effective deformation behavior as a compound effect of the voltage-induced and mechanical far-filed stress-induced beam-level deformations. In the present section, analytical formulations are divided into the following two sections (beam level and unit cell level) where individual deflections of each cell wall (i.e. idealized beams) under electromechanical loadings are obtained considering cell walls as Euler–Bernoulli beams. The influence of beam-level deformation has been extended further to the unit cell level to quantify the overall elastic deformation and electromechanical coupling phenomena of the honeycomb lattice. Note that both axial and bending contributions in the cell’s overall deformation are taken into consideration throughout the present formulation for achieving a high level of accuracy. Although it has been observed that the contribution of axial (and also shear) deformation is generally at a minimal level in comparison to that of bending unless *t*/*L* > 0.2 (cell wall’s thickness, *t*, and length, *L*) for hexagonal cellular lattices^48^.

In the following sections, we first present the beam-level deformation physics under multi-physical stimuli and subsequently, the lattice-level computational analysis is presented.

### Beam-level deformation physics

In the present study, the cell walls of the honeycomb are analyzed by considering it as Euler–Bernoulli beam with an out-of-plane width of *w*. Piezoelectric patches (e.g. PZT) are embedded onto the cell surfaces in an anti-symmetric manner, wherein in the first half of the wall, two piezo patches are attached to the top and bottom surfaces of the substrate layer. In the second half, the same piezo patches are attached in a reverse way (refer to Fig. 1B–D). During the application of the external remote mechanical stresses and under the application of voltage, each cell wall undergoes deformation where each node (joints) behaves as rotationally restrained to maintain a periodic deformed shape. In each member of the unit cell (i.e. representative volume element), the scheme of applied voltage is depicted in Fig. 1C that leads to an anti-symmetric ‘S’ curvature at the beam level. The end nodes of the cell walls are taken as rotationally restrained (this boundary condition satisfies the periodicity of the unit cells in deformed configurations), and the closed-form expression for such beam’s deformation is derived here using the fundamentals of Euler–Bernoulli beam theory. Due to the existence of symmetry in the bending moment diagram about the mid-point of the beam (with a null bending moment at the mid-point, as a result of the both-end rotationally restrained boundary condition), it is quite reasonable to consider the beam of length *L* as two cantilever beams of length *L*/2 joined at the mid-point. The total deflection (axial and transverse) will be the summation of the cantilevers’ respective deflections. Generally, piezo patches in such beam configuration will generate an equivalent moment and axial force in the system. The present formulation is developed directly from the very fundamental constitutive equation of piezoelectricity. Existing approximate equivalent moment and force approach^39,49^ are used later for validating the present formulation.

The honeycomb cell walls are embedded with piezoelectric patches on both sides in an alternating manner. Note that here this alteration denotes the change of polarization directions and voltages over its span. For the span 0 < *x* < *L*/2, the polarization direction in both piezo layers is along the *z*-direction whereas in *L*/2 < *x* < *L*, it is along the negative *z*-direction of the cell wall (*x*–*z* is the attached local in-plane coordinate system of each cell wall). The global coordinate system *X*–*Y* is attached to the lattice where *w* is the out-of-plane width of it. Since the lattice is periodic in nature, we can concentrate on one single representative volume element (or unit cell) and analyze it with appropriate periodic boundary conditions (as ensured by the rotationally restrained edges of the connecting beams) to obtain the global effective mechanical properties. In the present case, the tri-membered unit cell is shown with *t*_m_ and *t*_p_ as the thicknesses of the substrate (metallic) layer and piezo layers respectively. Under the simultaneous application of voltages on the outer piezo surfaces and far-field mechanical load to the lattice (refer to Fig. S1), the metamaterial will exhibit a coupling state between its normal and shear strains. Even under the simultaneous application of far-field mechanical stresses and electrical voltage, due to the existence of symmetry in the overall bending moment diagram, the full beam can be analyzed with the help of a cantilever-type bimorph beam with length *L*/2. Considering the sense of applied voltages on the beam surfaces and polarization directions of each layer (shown in Fig. 1D), the following constitutive stress-strain relations for each beam layer can be obtained:

1a $${\epsilon }_{x}^{{{u}}}={s}_{11}^{{ {E}}}{\sigma }_{x}^{{{u}}}-{d}_{31}\left(\frac{{V}_{{{u}}}}{{t}_{{{p}}}}\right)$$

1b $${\epsilon }_{x}^{ {{m}}}={s}_{11}^{{ {m}}}{\sigma }_{x}^{{ {m}}}$$

1c $${\epsilon }_{x}^{{ {l}}}={s}_{11}^{{ {E}}}{\sigma }_{x}^{{ {l}}}+{d}_{31}\left(\frac{{V}_{{{l}}}}{{t}_{{{p}}}}\right)$$

Here, (u, m, l) denotes the upper, mid and bottom layers of the beam, respectively. *ϵ*_*x*_ and *σ*_*x*_ indicate the internal uniaxial strain and stress generated across the beam’s cross-section due to the application of external mechanical (*F*_*x*_ and *F*_*y*_) and electrical loads (*V*_*u*_ and *V*_*l*_). *d*_31_ and ${s}_{11}^{{ {E}}}$ are the piezoelectric coupling coefficient and the elastic compliance of the piezoelectric element at a constant electric field, respectively. The thickness of each piezoelectric layer is taken as *t*_p_ whereas that of the substrate layer is taken as *t*_s_. The in-plane coordinate system (*z*−*x*) is taken along the neutral axis of the beam, located symmetrically w.r.t. two piezo layers.

Considering the principle of superposition under small deformation, axial and transverse deflection of the beam-like cell wall members can be obtained as (refer to subsections S2.1 and S2.2 of the supplementary material for detailed derivation)

2a $${\delta }_{x}=\frac{L\,{s}_{{{\rm{11}}}}^{{ {m}}}\,\left({F}_{x}\,{s}_{{{\rm{11}}}}^{{ {E}}}+{V}_{{{l}}}\,{d}_{31}\,w-{V}_{{{u}}}\,{d}_{31}\,w\right)}{w\,{s}_{{ {e}}}^{{ {T}}}}$$

2b $${\delta }_{z}=\frac{{L}^{3}\,{s}_{{{\rm{11}}}}^{{ {E}}}\,{s}_{{{\rm{11}}}}^{{ {m}}}}{w\,{s}_{{ {e}}}^{{ {t}}}}\,{F}_{y}-\frac{3{L}^{2}\,{d}_{31}\,{s}_{{{\rm{11}}}}^{ {{m}}}\,\left({t}_{{ {m}}}+{t}_{{{p}}}\right)}{2{s}_{{ {e}}}^{{ {t}}}}\,\left({V}_{{{u}}}+{V}_{{{l}}}\right)$$

where ${s}_{{ {e}}}^{{ {t}}}={s}_{{{\rm{11}}}}^{{ {E}}}\,{{t}_{ {{m}}}}^{3}+6\,{s}_{{{\rm{11}}}}^{{ {m}}}\,{{t}_{{ {m}}}}^{2}\,{t}_{{{p}}}+12\,{s}_{{{\rm{11}}}}^{ {{m}}}\,{t}_{{ {m}}}\,{{t}_{{{p}}}}^{2}+8\,{s}_{{{\rm{11}}}}^{{ {m}}}\,{{t}_{{{p}}}}^{3}$ and ${s}_{{ {e}}}^{{ {T}}}={s}_{11}^{ {{E}}}{t}_{{ {m}}}+2{s}_{11}^{{ {m}}}{t}_{{{p}}}$. In the following paragraph, we discuss some of the special cases that can be deduced for bi-morph beam elements.

Considering equation 2 and based on the polarity and magnitude of voltages (*V*_*u*_ and *V*_*l*_) applied on piezoelectric layers (refer to Fig. 1D), three possible actuation scenarios (mode) can be achieved. (I) *Hybrid actuation mode*: *V*_*u*_ ≠ *V*_*l*_ and thus, there will be both—axial and bending piezoelectric deformations of the beam due to the piezo-loads. Formulations in all the sections are done according to this mode to generalize the problem. (II) *Pure bending mode*: *V*_*u*_ = *V*_*l*_ and thus, there will be only piezoelectric bending deformation to the beam due to the piezo-loads. (III) *Pure axial mode*: *V*_*u*_ = −*V*_*l*_ and thus, there will be only piezoelectric axial deformation to the beam due to the piezo-loads. However, in all these aforementioned modes, mechanical deformations (both—axial and bending) will exist due to the external mechanical loads *F*_*x*_ and *F*_*y*_ applied on the beam. Therefore, the total deformation under the assumption of small strain, can be obtained as a superposition of the deformation components due to mechanical load and piezoelectric load.

### Lattice-level elastic stress and strain fields: Bottom-up beam-based computational framework

In this section, we discuss a bottom-up beam-based approach for obtaining the lattice-level deformation fields. The beam-level deformation physics discussed in the preceding section is extended here to the lattice level. Before going straight to the main formulation, it is imperative to discuss the deformation behavior of piezo-embedded vertical member of the unit cell. To illustrate that, an approximate piezoelectric moment-force term (*M*_V_ and *F*_V_) is introduced. Afterward, a simple bent problem is exemplified with the help of those two terms. An equivalent moment (*M*_V_) and axial force (*F*_V_) operating at the two opposite ends (nodes) of the beam can be used to simulate the effect of piezo patches on the deformation of the beam^49^. Figure S3A shows a two-noded and three-noded piezoelectric beam element and their equivalent moment-force arrangement. *M*_V_ can be obtained from the present formulation by equating *v*_*L*/2_ with the mechanical transverse deflection of a cantilever due to the moment *M*_V_. Similarly, *F*_V_ can be obtained by equating *u*_*L*/2_ with the axial deflection of a cantilever due to the axial force *F*_V_.

Analytical expressions of terms, *M*_V_ and *F*_V_ are not given here because these terms are not used in the present formulation, rather only shown for illustration purposes in later subsections. Now, to understand whether there will be any effect of applied voltages of the vertical member of the present unit cell on the slant members, one simple two-membered (AOB) bent structure is considered (Fig. S3B). The applied voltages of the vertical member (OA) are approximated by its equivalent moment-force terms (introduced previously) whereas the slant member (OB) is kept inactive. Considering the free-body diagram of AOB, the calculated internal bending moment, *M*(*x*) at any cut of the slant member (OB) can be given as: $M(x)=2{M}_{{ {V}}}-{M}_{{ {V}}}-{M}_{ {{V}}}+{F}_{{ {V}}}(L-x)\cos \theta -{F}_{{ {V}}}(L-x)\cos \theta =0$. As there is no bending moment generation due to *M*_V_ and *F*_V_ here, there will be no bending deformation of the slant member due to the applied voltage in the vertical member. The same has been verified with a three-membered unit cell in COMSOL (shown in Fig. S3C) where the voltage is applied on the vertical member only. It can be noted that there is no deformation to the slant members. All these observations can be summarized as: no deflection and slope changes occur in the slant members due to voltages applied on the vertical members of the honeycomb unit cell. This understanding has been utilized in succeeding sections to derive the effective elastic properties of the honeycomb metamaterial under different load cases.

To understand the nature of the coupled deformation field we will consider three different scenarios of far-field applied stresses, covering normal and shear stresses (refer to subsections S3.1, S3.2 and S3.3).). As shown in Fig. 1C, the voltages applied on vertical members of the unit cell are ${V}_{{{u}}}^{3}$ and ${V}_{{{l}}}^{3}$, whereas that of two slant members are ${V}_{{{u}}}^{{{s}}}$ and ${V}_{{{l}}}^{{{s}}}$. Under such electrical loading, in each member of the unit cell, there will be piezoelectric axial as well as bending deformations. In the present formulation, a linear-elastic deformation under a small strain assumption is used for the honeycomb.

Under the application of external mechanical uniaxial stress (*σ*_*x*_) in the *X*-direction, the effective normal and shear strain components can be obtained as

3a $${\epsilon }_{X}=\frac{{\delta }_{{ {BO}}}^{{ {b}}}\sin \theta +{\delta }_{{ {BO}}}^{{ {a}}}\cos \theta }{L\cos \theta }$$

3b $${\epsilon }_{{Y}}=-\frac{-{\delta }_{{ {BO}}}^{{ {a}}}\sin \theta +{\delta }_{{ {BO}}}^{ {{b}}}\cos \theta -{\delta }_{{ {AO}}}^{ {{a}}}}{h+L\sin \theta }$$

3c $${\gamma }_{XY}^{{ {C}}}=\frac{{\delta }_{{ {AO}}}^{{ {b}}}}{h+L\sin \theta }$$

In the above expressions, ${\delta }_{{ {BO}}}^{{ {b}}},{\delta }_{{ {BO}}}^{{ {a}}},{\delta }_{{ {AO}}}^{{ {a}}}$ and ${\delta }_{{ {AO}}}^{{ {b}}}$ can be obtained based on the beam-level deformation physics discussed in the preceding section (the exact expressions are also provided in subsection S3.1). The superscript C is used to denote coupled strain components, which would normally be absent in conventional materials. Subsequently, using the fundamental definitions of Young’s moduli and Poisson’s ratios, the following closed-form expressions can be obtained

4 $${E}_{1}=\frac{{\sigma }_{x}}{{\epsilon }_{X}}=\frac{{\lambda }_{1}^{{E}_{1}}\left({V}_{{{R}}}^{{{s}}}\right)}{{\lambda }_{2}^{{E}_{1}}\left({V}_{{{R}}}^{{{s}}}\right)+{\lambda }_{3}^{{E}_{1}}\left({V}_{{{R}}}^{{{s}}}\right)\left(\frac{{V}_{{{u}}}^{{{s}}}}{{\sigma }_{x}}\right)}$$

5 $${\nu }_{12}=-\frac{{\epsilon }_{Y}}{{\epsilon }_{X}}=\frac{{\beta }_{1}^{{\nu }_{12}}\left({V}_{{{R}}},{V}_{{{R}}}^{{{s}}},{V}_{{{R}}}^{3}\right)+{\beta }_{2}^{{\nu }_{12}}\left({V}_{{{R}}},{V}_{{{R}}}^{{{s}}},{V}_{{{R}}}^{3}\right)\left(\frac{{V}_{{{u}}}^{{{s}}}}{{\sigma }_{x}}\right)}{{\beta }_{3}^{{\nu }_{12}}\left({V}_{{{R}}},{V}_{{{R}}}^{{{s}}},{V}_{{{R}}}^{3}\right)+{\beta }_{4}^{{\nu }_{12}}\left({V}_{{{R}}},{V}_{{{R}}}^{{{s}}},{V}_{{{R}}}^{3}\right)\left(\frac{{V}_{{{u}}}^{{{s}}}}{{\sigma }_{x}}\right)}$$

Here the expressions of the coefficients (${\lambda }_{i}^{{E}_{1}}$ and ${\beta }_{i}^{{\nu }_{12}}$) are given in section S6 of supplementary material where the following ratios have been used: ${L}_{{{R}}}=\frac{h}{L},{t}_{{{R}}}=\frac{{t}_{{ {m}}}}{{t}_{{{p}}}},{s}_{11}^{{{R}}}=\frac{{s}_{11}^{{ {m}}}}{{s}_{11}^{{ {E}}}},{V}_{{{R}}}=\frac{{V}_{{{u}}}^{{{s}}}}{{V}_{{{u}}}^{3}},{V}_{{{R}}}^{{{s}}}=\frac{{V}_{{{u}}}^{{{s}}}}{{V}_{{{l}}}^{{{s}}}},{V}_{{{R}}}^{3}=\frac{{V}_{{{u}}}^{3}}{{V}_{{{l}}}^{3}}$. Note that the coefficients, ${\lambda }_{i}^{{E}_{1}}$ are the functions of ${V}_{{{R}}}^{{{s}}}$ only, whereas ${\beta }_{i}^{{\nu }_{12}}$ are the functions of ${V}_{{{R}}},{V}_{{{R}}}^{{{s}}}$, and ${V}_{{{R}}}^{3}$. From the expressions as functions of *σ*_*x*_ and ${V}_{{{u}}}^{{{s}}}$ in Eqs. (4) and (5), it is evident that the voltage applied on the vertical member has an influence on the effective Poission’s ratio of the lattice, whereas the effective Young’s modulus is solely controllable by voltages on the slant members.

Under the application of external mechanical uniaxial stress (*σ*_*y*_) in *Y*-direction, the effective normal and shear strain components can be obtained as

6a $${\epsilon }_{X}=-\frac{-{\delta }_{{ {BO}}}^{ {{a}}}\cos \theta +{\delta }_{{ {BO}}}^{{ {b}}}\sin \theta }{L\cos \theta }$$

6b $${\epsilon }_{{ {Y}}}=\frac{{\delta }_{{ {AO}}}^{{ {a}}}+{\delta }_{{ {BO}}}^{{ {b}}}\cos \theta +{\delta }_{{ {BO}}}^{{ {a}}}\sin \theta }{h+L\sin \theta }$$

6c $${\gamma }_{XY}^{{ {C}}}=\frac{{\delta }_{ {{AO}}}^{{ {b}}}}{h+L\sin \theta }$$

In the above expressions, ${\delta }_{ {{BO}}}^{{ {b}}},{\delta }_{{ {BO}}}^{{ {a}}},{\delta }_{{ {AO}}}^{ {{a}}}$ and ${\delta }_{ {{AO}}}^{{ {b}}}$ can be obtained based on the beam-level deformation physics discussed in the preceding section (the exact expressions are also provided in subsection S3.2). Subsequently, using the fundamental definitions of Young’s moduli and Poisson’s ratios, the following closed-form expressions can be obtained:

7 $${E}_{2}=\frac{{\sigma }_{{ {Y}}}}{{\epsilon }_{{ {Y}}}}=\frac{{\lambda }_{1}^{{E}_{2}}\left({V}_{{{R}}},{V}_{{{R}}}^{{{s}}},{V}_{{{R}}}^{3}\right)}{{\lambda }_{2}^{{E}_{2}}\left({V}_{{{R}}},{V}_{{{R}}}^{{{s}}},{V}_{{{R}}}^{3}\right)+{\lambda }_{3}^{{E}_{2}}\left({V}_{{{R}}},{V}_{{{R}}}^{{{s}}},{V}_{{{R}}}^{3}\right)\left(\frac{{V}_{{{u}}}^{{{s}}}}{{\sigma }_{{ {y}}}}\right)}$$

8 $${\nu }_{21}=-\frac{{\epsilon }_{X}}{{\epsilon }_{Y}}=\frac{{\beta }_{1}^{{\nu }_{21}}\left({V}_{R},{V}_{R}^{s},{V}_{R}^{3}\right)+{\beta }_{2}^{{\nu }_{21}}\left({V}_{R},{V}_{R}^{s},{V}_{R}^{3}\right)\left(\frac{{V}_{u}^{s}}{{\sigma }_{y}}\right)}{{\beta }_{3}^{{\nu }_{21}}\left({V}_{R},{V}_{R}^{s},{V}_{R}^{3}\right)+{\beta }_{4}^{{\nu }_{21}}\left({V}_{R},{V}_{R}^{s},{V}_{R}^{3}\right)\left(\frac{{V}_{u}^{s}}{{\sigma }_{y}}\right)}$$

The expressions of the coefficients (${\lambda }_{i}^{{E}_{2}}$ and ${\beta }_{i}^{{\nu }_{12}}$) are given in section S7 of supplementary material where the same ratios as mentioned earlier have been used to simplify the expressions. Note that both the coefficients, ${\lambda }_{i}^{{E}_{2}}$ and, ${\beta }_{i}^{{\nu }_{21}}$ are the functions of ${V}_{{{R}}},{V}_{{{R}}}^{{{s}}}$, and ${V}_{{{R}}}^{3}$. Similar dependency trends of voltages and elastic properties (refer to Eqs. (4) and (5)) can be observed in Eqs. (7) and (8). However, contrary to dependency trends of prior *X*-directional far-field stress (refer to Eqs. (4) and (5)), both the effective Poisson’s ratio and the effective Young’s modulus in *Y*-directional far-field stress are influenced by the voltage applied on the vertical member.

Under the application of external far-field shear stress (*τ*_*XY*_), the effective normal and shear strain components can be obtained as

9a $${\epsilon }_{X}^{{ {C}}}=\frac{{\delta }_{{ {B0}}}^{{ {A}}}\cos \theta +{\delta }_{{ {BO}}}^{{ {b}}}\sin \theta }{L\cos \theta }$$

9b $${\epsilon }_{Y}^{{ {C}}}=\frac{{\delta }_{{ {AO}}}^{{ {a}}}+{\delta }_{{ {BO}}}^{{ {A}}}\sin \theta -{\delta }_{{ {BO}}}^{{ {b}}}\cos \theta }{h+L\sin \theta }$$

9c $${\gamma }_{XY}=\frac{{\delta }_{{ {AO}}}^{{{s}}}}{(h+L\sin \theta )}+\frac{{\delta }_{{ {BO}}}^{{ {a}}}\cos \theta }{(h+L\sin \theta )}+2\frac{{\delta }_{ {{BO}}}^{{ {a}}}\sin \theta }{(2L\cos \theta )}$$

In the above expressions, ${\delta }_{{{BO}}}^{{{b}}},{\delta }_{{{BO}}}^{{{A}}},{\delta }_{{{BO}}}^{{{a}}},{\delta }_{{{AO}}}^{{{a}}}$ and ${\delta }_{{{AO}}}^{{{b}}}$ can be obtained based on the beam-level deformation physics discussed in the preceding section (the exact expressions are also provided in subsection S3.3). Subsequently, using the fundamental definitions of shear modulus, the following closed-form expression can be obtained

10 $${G}_{12}=\frac{{\tau }_{XY}}{{\gamma }_{XY}}=\frac{{\lambda }_{1}^{{G}_{12}}\left({V}_{{{R}}},{V}_{{{R}}}^{3}\right)}{{\lambda }_{2}^{{G}_{12}}\left({V}_{{{R}}},{V}_{{{R}}}^{3}\right)+{\lambda }_{3}^{{G}_{12}}\left({V}_{{{R}}}^{3}\right)\left(\frac{{V}_{{{u}}}^{{{s}}}}{{\tau }_{XY}}\right)}$$

The coefficients ${\lambda }_{i}^{{G}_{12}}(i=1,2,3)$ are given in section S8 of supplementary material. Here the coefficients, ${\lambda }_{1}^{{G}_{12}}$ and ${\lambda }_{2}^{{G}_{12}}$ are the functions of ${V}_{{{R}}},{V}_{{{R}}}^{3}$, whereas ${\lambda }_{3}^{{G}_{12}}$ is the function of ${V}_{{{R}}}^{3}$ only. It can be noted that the shear modulus is voltage-dependent in the proposed metamaterial along with Young’s moduli and Poisson’s ratios.

From Eqs. (3) and (6), it may be noted that for uniaxial normal loading cases, the voltages applied on the vertical member (${V}_{{{u}}}^{3}$ and ${V}_{{{l}}}^{3}$) will be solely responsible for the generation of coupled shear deformation in the unit cell. In other words, these two deformations (normal and shear) will have independently controllable capabilities if two different voltages are applied on the slant and vertical member i.e. ${V}_{{{u}}}^{3}\ne {V}_{{{u}}}^{{{s}}}$ and ${V}_{{{l}}}^{3}\ne {V}_{{{l}}}^{{{s}}}$. On the other hand, they will become coupled (dependent) if equal voltages are applied throughout the unit cell i.e. ${V}_{{{u}}}^{3}={V}_{{{u}}}^{{{s}}}={V}_{{{u}}}$ and ${V}_{{{l}}}^{3}={V}_{ {{l}}}^{{{s}}}={V}_{{{l}}}$. In the latter scenario, voltage (*V*_*u*_ and *V*_*l*_) becomes the only external coupling factor.

Contrary to uniaxial normal loading cases, an opposite trend has been observed in shear loading case. It can be seen from Eq. (9) that voltages on slant member (${V}_{{{u}}}^{{{s}}}$ and ${V}_{{{l}}}^{{{s}}}$) will only induce normal deformation to the unit cell. Applied shear and coupled normal modes will become dependent only if equal voltages are applied throughout the unit cell i.e. ${V}_{{{u}}}^{3}={V}_{{{u}}}^{{{s}}}={V}_{{{u}}}$ and ${V}_{{{l}}}^{3}={V}_{{{l}}}^{{{s}}}={V}_{{{l}}}$. External mechanical stresses (*σ*_*X*_ or *σ*_*Y*_ or *τ*_*XY*_) will have no influences on the coupled deformation in their respective load cases. It’s only external voltage that will play the role of such coupling phenomena. As the primary objective of the present work is to highlight axial–shear coupling phenomena, the aforementioned coupling condition (i.e. ${V}_{{{u}}}^{3}={V}_{{{u}}}^{{{s}}}={V}_{{{u}}}$ and ${V}_{{{l}}}^{3}={V}_{{{l}}}^{{{s}}}={V}_{{{l}}}$) is used afterward for presenting numerical results unless otherwise mentioned.

## Results and discussion

### Multi-level validation of the computational framework

Before presenting the computational results on active normal–shear mode coupling, it is imperative to validate the accuracy of the current analytical model both at the cell wall (beam) as well as unit cell (RVE) levels. We discuss the beam-level validation first, wherein the boundary conditions are taken the same as one end fixed and the other end rotationally restrained (to maintain the periodicity in the unit cells as discussed earlier). Throughout the beam validation, PZT5H (lead zirconate titanate) and aluminum are used as piezoelectric and substrate materials, respectively. Piezoelectric poling directions are taken normal to the beam’s longitudinal axis. The same active beam has been modeled within the Piezoelectricity multiphysics interface of the COMSOL Multiphysics FEA (finite element analysis) environment for performing static analysis using triangular finer mesh elements. A perfect bonding interface between the piezo layer and substrate layer is assumed and the condition of ground-electrode (*V* = 0) is applied at this interface only.

We have considered different deformation modes for the beam-level validation. First, the pure-bending-deformation condition is established by considering only transverse mechanical force (*F*_*y*_) applied on one end of the beam. The piezoelectric voltage applied on the upper surface is taken as the same magnitude and of the same polarity as that of the bottom surface (*V*_*u*_ = *V*_*l*_). It will result in a traverse deflection to the beam, with no axial deformation. Figure S5 shows the linear variation of the deflection with its two external input parameters. Figure S5A and B highlight the independent influences of voltage and force where one of them is taken as zero, whereas, in Fig. S5C and D, both are taken as non-zero.

Figure S6 highlights a pure-axial-deformation scenario where only an axial load (*F*_*x*_) is applied and voltages are taken as *V*_*u*_ = −*V*_*l*_. This too provides a linear variation with such external loads. It can be observed that the cell walls (i.e. composite piezoelectric beams) are bending-dominant as their axial deformations are noticeably less than the transverse deformation. For example, for the current beam with no external mechanical loads, the ratio of axial piezoelectric deformation to transverse piezoelectric deformation is in the order of  ~10^−3^. A good agreement unfolds when comparing the results of the current beam-level formulation with those of the matrix-based technique and COMSOL. It gives us adequate confidence to implement Eq. (2) in deriving the elastic deformation at the lattice level based on unit cell deformations. Though Figs. S5 and S6 demonstrate the influence of external load parameters separately, it has also been confirmed that present closed-form expressions for beam deformation (*δ*_*x*_ and *δ*_*z*_) are equally applicable to the system in which all loads (*F*_*x*_ ≠ 0, *F*_*y*_ ≠ 0, *V*_s_ ≠ 0 and *V*_*l*_ ≠ 0) are active.

Due to the lack of literature on smart honeycomb structures with the present bi-level architected piezo-configuration, three different validation methodologies have been adopted at the unit cell and lattice level instead of direct comparison with available data. (1) The lattice level effective elastic properties are compared with the closed-form results of available literature^48^ where the thickness of piezoelectric components is considered as zero (i.e. conventional mono-material honeycomb lattices) as a special case. (2) The results are compared with a unit cell-based assembled direct stiffness method (matrix approach) conceptualized in the present work with the help of beam-level stiffness matrices including piezo elements. Note that such a direct and brute-force analytical method in the context of active lattice analysis is proposed for the first time here considering an appropriate unit cell and periodic boundary conditions. Though such an approach is more straightforward (compared to the bottom-up approach proposed in the preceding sections) once the appropriate unit cell and boundary conditions are figured out, it is computationally expensive and less insightful. (3) Further, the lattices are simulated in one computational finite element package to achieve more confidence. For the sake of flow in the understanding, the assembled direct stiffness method and the approach based on the commercial finite element package are described in the following two paragraphs before presenting the validation results at the unit cell and lattice level.

A modified stiffness matrix and an equivalent piezoelectric load vector for a 2D two-noded piezo-embedded hybrid beam (bimorph) element can be derived by using strain-energy principles and variational method considering the present hybrid beam (refer to Fig. 1D)^39^. If the beam element of length *L* and out-of-plane width *w*, having three degrees of freedom (axial, transverse and rotational deformations) in each node is subjected to the external voltages *V*_*u*_ and *V*_*l*_ on the upper and bottom piezo layer, respectively, its 6 × 6 stiffness matrix [*K*] and load vector [*F*_*p*_] can be given as follows:

11a $$K=\left[\begin{array}{cccccc}\frac{{E}_{i}{A}_{i}}{L}&0&-\frac{{E}_{i}{H}_{i}}{L}&-\frac{{E}_{i}{A}_{i}}{L}&0&\frac{{E}_{i}{H}_{i}}{L}\\ 0&12\frac{{E}_{i}{I}_{i}}{{L}^{3}}&6\frac{{E}_{i}{I}_{i}}{{L}^{2}}&0&-12\frac{{E}_{i}{I}_{i}}{{L}^{3}}&6\frac{{E}_{i}{I}_{i}}{{L}^{2}}\\ -\frac{{E}_{i}{H}_{i}}{L}&6\frac{{E}_{i}{I}_{i}}{{L}^{2}}&4\frac{{E}_{i}{I}_{i}}{L}&\frac{{E}_{i}{H}_{i}}{L}&-6\frac{{E}_{i}{I}_{i}}{{L}^{2}}&2\frac{{E}_{i}{I}_{i}}{L}\\ -\frac{{E}_{i}{A}_{i}}{L}&0&\frac{{E}_{i}{H}_{i}}{L}&\frac{{E}_{i}{A}_{i}}{L}&0&-\frac{{E}_{i}{H}_{i}}{L}\\ 0&-12\frac{{E}_{i}{I}_{i}}{{L}^{3}}&-6\frac{{E}_{i}{I}_{i}}{{L}^{2}}&0&12\frac{{E}_{i}{I}_{i}}{{L}^{3}}&-6\frac{{E}_{i}{I}_{i}}{{L}^{2}}\\ \frac{{E}_{i}{H}_{i}}{L}&6\frac{{E}_{i}{I}_{i}}{{L}^{2}}&2\frac{{E}_{i}{I}_{i}}{L}&-\frac{{E}_{i}{H}_{i}}{L}&-6\frac{{E}_{i}{I}_{i}}{{L}^{2}}&4\frac{{E}_{i}{I}_{i}}{L}\end{array}\right]$$

11b $${F}_{{{p}}}=\left\{\begin{array}{cccccc}{B}_{{{p}}}\frac{({V}_{{{u}}}-{V}_{{{l}}})}{2}&0&-{J}_{{{p}}}\frac{({V}_{{{u}}}+{V}_{{{l}}})}{2}&-{B}_{{{p}}}\frac{({V}_{{{u}}}-{V}_{{{l}}})}{2}&0&{J}_{{{p}}}\frac{({V}_{{{u}}}+{V}_{{{l}}})}{2}\end{array}\right\}$$

Here the dummy index *i* denotes the piezo (p) layers (upper and bottom layer both) and middle substrate (m) layer of the beam and therefore, *E*_*i*_*A*_*i*_ = *E*_*p*_*A*_*p*_ + *E*_*m*_*A*_*m*_, *E*_*i*_*H*_*i*_ = *E*_*p*_*H*_*p*_ + *E*_*m*_*H*_*m*_ and *E*_*i*_*I*_*i*_ = *E*_*p*_*I*_*p*_ + *E*_*m*_*I*_*m*_. The values of *A*_*i*_, *H*_*i*_, *I*_*i*_, *B*_*p*_ and *J*_*p*_ can be given as: $$\left\{\begin{array}{ccc}{A}_{i}&{H}_{i}&{I}_{i}\end{array}\right\}={\iint }_{i(= {{m,p}})}\left\{\begin{array}{ccc}1&z&{z}^{2}\end{array}\right\}\,{ {d}}y\,{ {d}}z$$ and $$\left\{\begin{array}{cc}{B}_{{{p}}}&{J}_{{{p}}}\end{array}\right\}={\iint }_{{{p}}}\frac{{d}_{31}}{{t}_{{{p}}}{s}_{11}^{{{E}}}}\left\{\begin{array}{cc}1&z\end{array}\right\}\,{{d}}y\,{{d}}z$$. The current unit cell in Fig. 1C can be considered as a symmetric triangular assembly of six two-noded hybrid beam elements. Applying the modified stiffness matrix ([*K*]) and the equivalent piezo-load vector (*F*_*p*_) along with typical mechanical load vector (*F*_*m*_), the equilibrium equation of each two-noded beam element at their local coordinate systems can be written as [*K*]{*ϕ*} = [*F*] = [*F*_*p*_] + [*F*_*m*_]. After assembling all the matrices, the global force–displacement equation of the unit cell is obtained through symbolic computing and solved under appropriate periodic mechanical boundary and loading conditions. For mechanical nodal forces and moments, the same shown in Figs. S2 and S4 have been followed. Note that the assembled direct stiffness method involves a range of matrix operations including, evaluation of the individual beam stiffness matrices of the constituting beams in a unit cell, determination of the orientation matrices of each of the members, conversion of the individual beam-level stiffness matrices from local to global exes system, structural assembly to obtain the global stiffness matrices, and evaluation of the load vector, followed by imposition of periodic boundary condition and computationally intensive matrix inversion. While mechanically straightforward to implement, this approach becomes computationally intensive. On the contrary, the proposed bottom-up approach leads to direct closed-form expressions for the normal and shear deformations along with the effective elastic moduli of the active lattices, making it computationally efficient and physically insightful.

As a separate method of unit cell level validation, the same active lattice unit cell has also been modeled within the Piezoelectricity multiphysics interface of COMSOL Multiphysics FEA environment for performing static analysis using triangular finer mesh elements (13,091 elements). Throughout the validation, PZT5H (lead zirconate titanate) and aluminum are used as piezoelectric and substrate materials, respectively. As piezoelectric poling directions are taken normal to the walls’ longitudinal axes, six different coordinate systems are defined using base vectors for three cell walls where the third axis directions are aligned with respective poling directions. A perfect bonding interface between the piezo layer and substrate layer is assumed and the condition of ground-electrode (*V* = 0) is applied at this interface only. Rotations about out-of-plane axis are restricted at the outer three nodes of the RVE for normal loading cases, whereas in shear loading cases, equivalent reaction moments (refer to Fig. S4) are applied at the nodes manually. For each FEA simulation, the parameters that are taken constant are summarized in Table S1. An initial validation for the present unit cell-based approach at the lattice level can be made if we render each hybrid member of the lattice close to a non-piezoelectric mono-material linear elastic member. This can be done by simply changing the following parameters as ${s}_{11}^{{{E}}}\to {s}_{11}^{{{m}}}$ and *V*_*u*_ = *V*_*l*_ → 0 (i.e. ${V}_{{{R}}}^{{{s}}}={V}_{{{R}}}^{3}\to 0$) in our derived closed-formed expressions. It will then converge to the closed-form expressions of effective elastic moduli from the existing references^17,48^, corroborating the accuracy and validity of the present analytic model in an exact manner through a special case. Subsequently to include the piezoelectric effect, a hybrid bending-dominant unit cell structure is taken into account. Here the term, hybrid, implies the condition: *V*_*u*_ ≠ *V*_*l*_. The term, bending-dominant, indicates the fact that axial deformation due to both mechanical as well as piezoelectric effects is ignored as the ratio of axial to bending deformation is around 10^−3^ ~ 10^−4^ for the considered parameters (refer to Table S1).

Figure S7 shows the elastic strain variations (comparative results) for all three loading cases, associated with their coupled state where external stress (tensile and shear) is varied between 10 and 1000 N m^−2^. The ratio of voltages on the upper and lower surfaces of the piezoelectric layer in all three members of the unit cell is taken between −2 and 2. As the present formulations are performed under small deformation assumption, linear relations between stress and strain are observed in the plots. Figure S7A–I reveal an important fact that the parameter *V*_*u*_/*V*_*l*_ can reverse the lattice’s tensile effect. For instance, as shown in Fig. S7B, compressive deformation begins to take place in a certain range of *V*_*u*_/*V*_*l*_ ratio (*V*_*u*_/*V*_*l*_ = − 2 to  −1.3) when a constant tensile load (*σ*_*x*_ = 100 N m^−2^) is applied to the system. The results obtained from both the unit cell-based assembled direct stiffness method (matrix approach) and finite element-based approach are noted to be in excellent agreement with the beam-based analytical approach, leading to adequate confidence for extending the investigation further concerning normal–shear coupling phenomena.

Coming to the validity of the fundamental reciprocal theorem in the context of effective elastic moduli, it has been verified numerically that the following relation holds good for all the lattice configurations, applied far-field stresses and voltages: ${\lim }_{{V}_{{{u}}}^{{{s}}}\to 0}\frac{{E}_{1}{\nu }_{21}}{{E}_{2}{\nu }_{12}}=1$. This enhances reliance on the analytical derivations concerning strain fields and the expressions of effective elastic constants.

To summarize, we have proposed an insightful bottom-up beam-based analytical framework for exploring the normal–shear coupling and effective elastic moduli of the proposed active lattices. The bottom-up beam-based analytical framework is further validated with the unit cell-based assembled direct stiffness method (matrix approach) and finite element-based approach considering active elements. In addition, we present an exact lattice-level analytical validation with respect to available literature considering the special case of passive conventional lattices. The beam-level deformation physics is separately validated before proceeding to the lattice-level validation. Such an extensive validation considering elementary beam level and lattice level and involving multiple approaches would garner adequate confidence in the computational results discussed herein.

### Active mode coupling and constitutive programming of elastic moduli

#### Normal-shear mode coupling

**Fig. 2:**
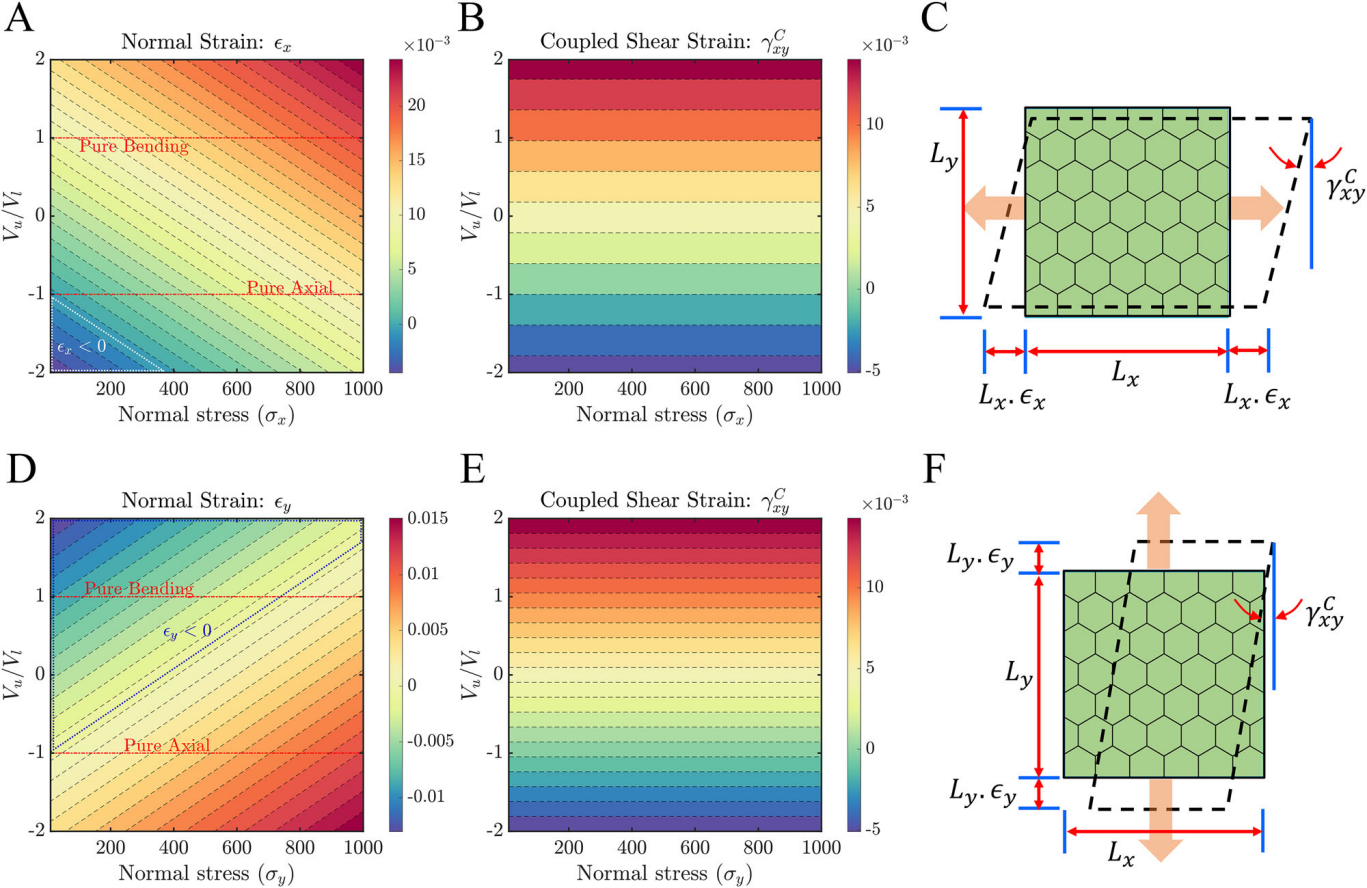
Normal-shear mode coupling under the application of normal far-field stress. Contour plots are presented for the induced strain of a regular honeycomb lattice (*L*_R_ = 1 and *θ* = 30) as a function of the applied direct stress (*σ*) and piezoelectric voltage ratio (*V*_*u*_/*V*_*l*_). **A** and **B** Normal strain (*ϵ*_*x*_) and its associated shear strain (${\gamma }_{xy}^{C}$) when normal far-field stress is applied along the X-direction. **C** Schematic visualization of (*X*-directional) normal and associated shear strain at lattice level. **D** and **E** Normal strain (*ϵ*_*y*_) and its associated shear strain (${\gamma }_{xy}^{ {{C}}}$) when normal far-field stress is applied along the *Y*-direction. **F** Schematic visualization of (*Y*-directional) normal and associated shear strain at lattice level. Note that the white (in sub-figure **A**) and blued-dotted (in sub-figure **D**) regions represent the negative strain (compression) when tensile stress is given as input. The red-dotted (in sub-figures **A** and **D**) lines highlight the two different piezoelectric actuation modes i.e. pure-bending (*V*_*u*_ = *V*_*l*_) and pure-axial (*V*_*u*_ = −*V*_*l*_). The normal stress is given in N m^−2^ units.

The present section elaborates on the normal–shear coupling correlation of the current hybrid active honeycomb lattice. To achieve the coupling, the aforementioned relation (i.e.${V}_{{{u}}}^{3}={V}_{{{u}}}^{{{s}}}={V}_{{{u}}}$ and ${V}_{{{l}}}^{3}={V}_{{{l}}}^{{{s}}}={V}_{{{l}}}$) between voltages of the cell members is conformed throughout this section. The external tensile stresses and voltages are considered here in the range of 10–1000 N m^−2^ and  −200 to 200 V, respectively. Therefore, the hybrid voltage ratio (*V*_*u*_/*V*_*l*_) in each member is adjusted between −2 and 2, while the voltage in the bottom layer is maintained at 100 V. Figure 2A and B shows the output normal strain and its coupled in-plane shear strain when an external axial stress *σ*_*x*_ is applied to the lattice along *X*-direction. Figure 2A represents the linear dependency of normal strain (*ϵ*_*x*_) with applied normal stress and voltage ratio where both of these parameters are in direct relation with *ϵ*_*x*_. For a certain range of voltage and normal stress, the domination of voltage-induced load over mechanical applied load can reverse the sense of output axial strain which is shown by the white-dotted triangular region in Fig. 2A. For example, for a voltage ratio of (−1.8) and applied *x*-directional tensile stress of 60 N m^−2^, compression along *x*-direction is being shown by the lattice with a strain value of 0.0029, establishing the notion of negative Young’s modulus. Additionally, it can be noticed that the strain values close to the member’s piezoelectric pure bending state are higher than its pure axial state. It has also been verified by plotting separately the same normal strain with respect to the strain at its piezoelectric pure bending state (*V*_*u*_ = *V*_*l*_) (refer to Supplementary Fig. S8 for further insights). This implies that the current lattice is a bending-dominant structure for the given piezoelectric material and structural parameters (refer to Table S1). The same is true for coupled shear strain (${\gamma }_{xy}^{{{C}}}$) as well which is shown in Fig. 2B.

Figure 2D and E depicts the strain spectrums for *Y*-directional normal loading case (considering both the magnitude and signs of the strains). The ranges of hybrid voltage ratio and external stress are kept the same as prior loading cases. In contrast to the previous *X*-directional loading, the current *Y*-directional tensile loading state exhibits an increase in output normal strain *ϵ*_*y*_ with applied stress but a decrease in voltage ratio. Further, in comparison to the prior loading instance, there has been a larger area in Fig. 2D where the output strain’s sign inverts (shown by a dotted border). Note that in *X*-, *Y*-directional normal states and their associated shear states, it has been noticed that the zero-strain line (*ϵ*_*x*/*y*/*xy*_ = 0) divides the spectrum into two regions where the strain (absolute) values are symmetric with respect to one another, albeit having opposite sense. In Fig. 2D, for instance, the combination of *V*_*u*_/*V*_*l*_ = 0.5858, *σ*_*x*_ = 500 N m^−2^, and *V*_*u*_/*V*_*l*_ = 0.30303, *σ*_*x*_ = 570 N m^−2^ can yield a normal strain with a magnitude of 0.001013 since these two are situated on level lines that are symmetric with regard to the zero-strain line. Coming to its coupled shear strain ${\gamma }_{xy}^{{ {C}}}$ in Fig. 2E, the trend is similar to that of *X*-directional loading scenario as it is contributed by the vertical member under the same piezoelectric loading. Note that the clockwise and anticlockwise modes of shear strain generation are observed in the piezoelectric pure-axial state in both cases (refer to Fig. 2B and E). In other words, shear deformation occurs in the anti-clockwise sense in all other regions except in the range of −1 and −2 of the hybrid voltage ratio, where clockwise shear deformation develops.

**Fig. 3:**
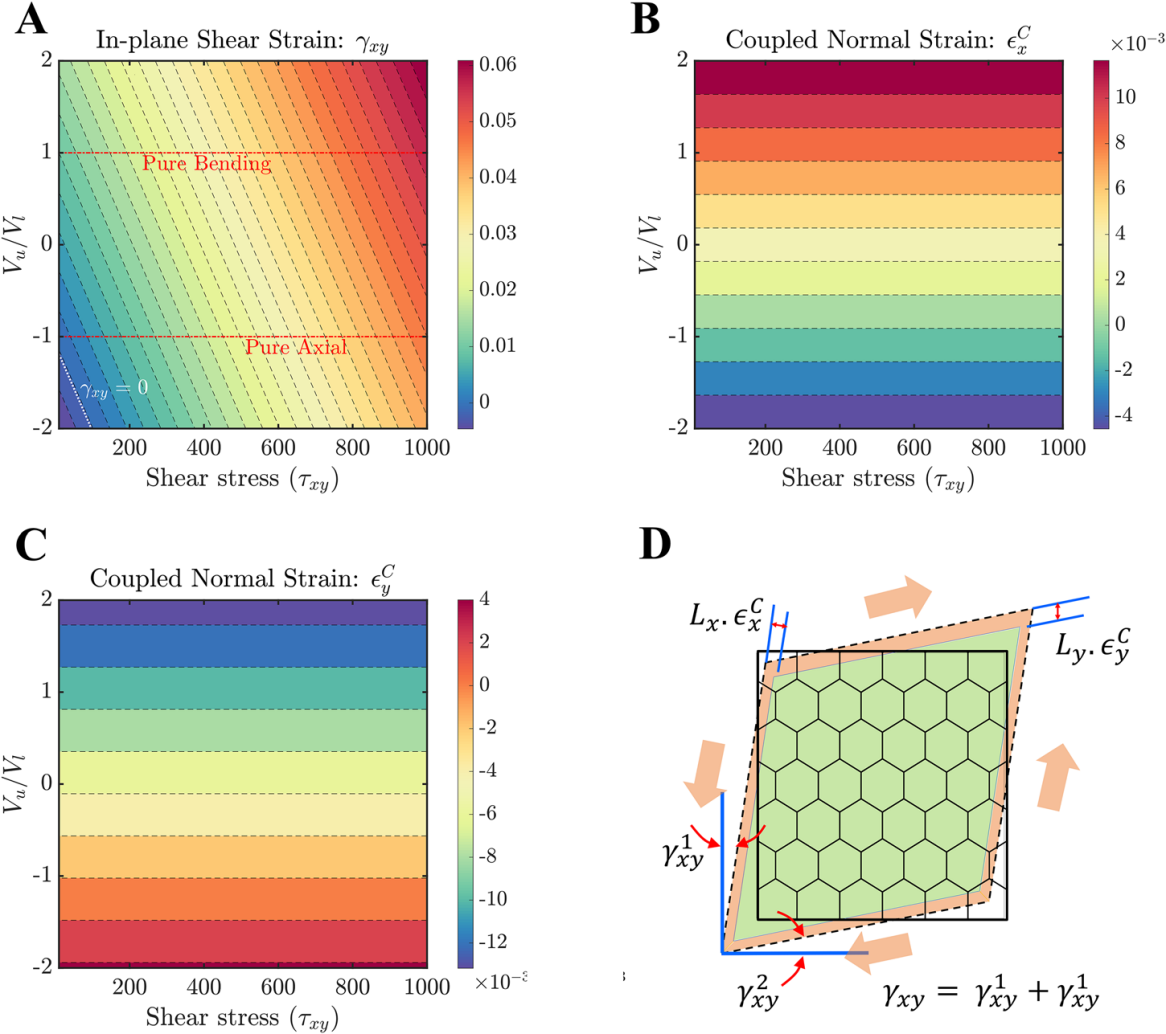
Normal-shear mode coupling under the application of far-field shear stress. Contour plots are presented for induced in-plane shear strain and coupled normal strains in a regular honeycomb lattice (*L*_R_ = 1 and *θ* = 30) as the function of applied shear stress (*τ*_*xy*_) and the piezoelectric voltage ratio (*V*_*u*_/*V*_*l*_). **A** Variation of shear strain (*γ*_*xy*_) when the far-field shear stress is applied in an anti-clockwise direction. **B**, **C** Coupled normal strain (*ϵ*^C^) along *X* and *Y*-direction of the lattice, when a far-field shear stress is applied. **D** Schematic visualization of in-plane shear and associated normal strains under the application of far-field shear stress. Note that in sub-figure A, the white-dotted line represents the zero-shear strain line (*γ*_*xy*_ = 0), while the red-dotted lines highlight two different piezoelectric actuation modes i.e. pure-bending (*V*_*u*_ = *V*_*l*_) and pure-axial (*V*_*u*_ = −*V*_*l*_). The shear stress is given in N m^−2^ units.

Figure 3A–C shows the variation in shear strain and its associated coupled normal strains in two directions (*X* and *Y*) of the lattice under external shear stress. Both the external loading parameters (applied shear stress and piezoelectric voltage ratio) are in direct relation with in-plane shear strain *γ*_*xy*_. All combinations of the loading parameters shown in Fig. 3A result in positive shear deformation, with the exception of those whose values fall beyond the zero-strain line (*γ*_*xy*_ = 0). For example, the present lattice gives a negative shear strain of magnitude 0.002 when a positive shear stress of 40 N m^−2^ is applied to it in the presence of an applied hybrid voltage ratio of  −1.72. This establishes the notion of negative shear moduli from a purely mechanical viewpoint. For the current external shear stress range investigated here, it has been observed that the strain values at the pure-axial state are roughly 18–90% lower than its pure-bending state. Furthermore, the coupled normal strains in *X* and *Y*-directions show a symmetric behavior with respect to the pure-axial state (refer to Fig. 3B and C). In other words, at pure-axial state (*V*_*u*_/*V*_*l*_ = −1), both give zero normal strain and the sense of strains beyond this state is opposite to each other. For instance, tensile strain is obtained along the *X*-direction in the voltage ratio range (−1 to 2), whilst compression of almost the same magnitude occurs in the *Y*-direction. It is important to note that in all the aforementioned load cases, the definition of hybrid voltage ratio is kept the same for both the slant and vertical member. Two different hybrid voltage ratios on these two members will give the lattice more flexibility in controlling the global strain field.

**Fig. 4:**
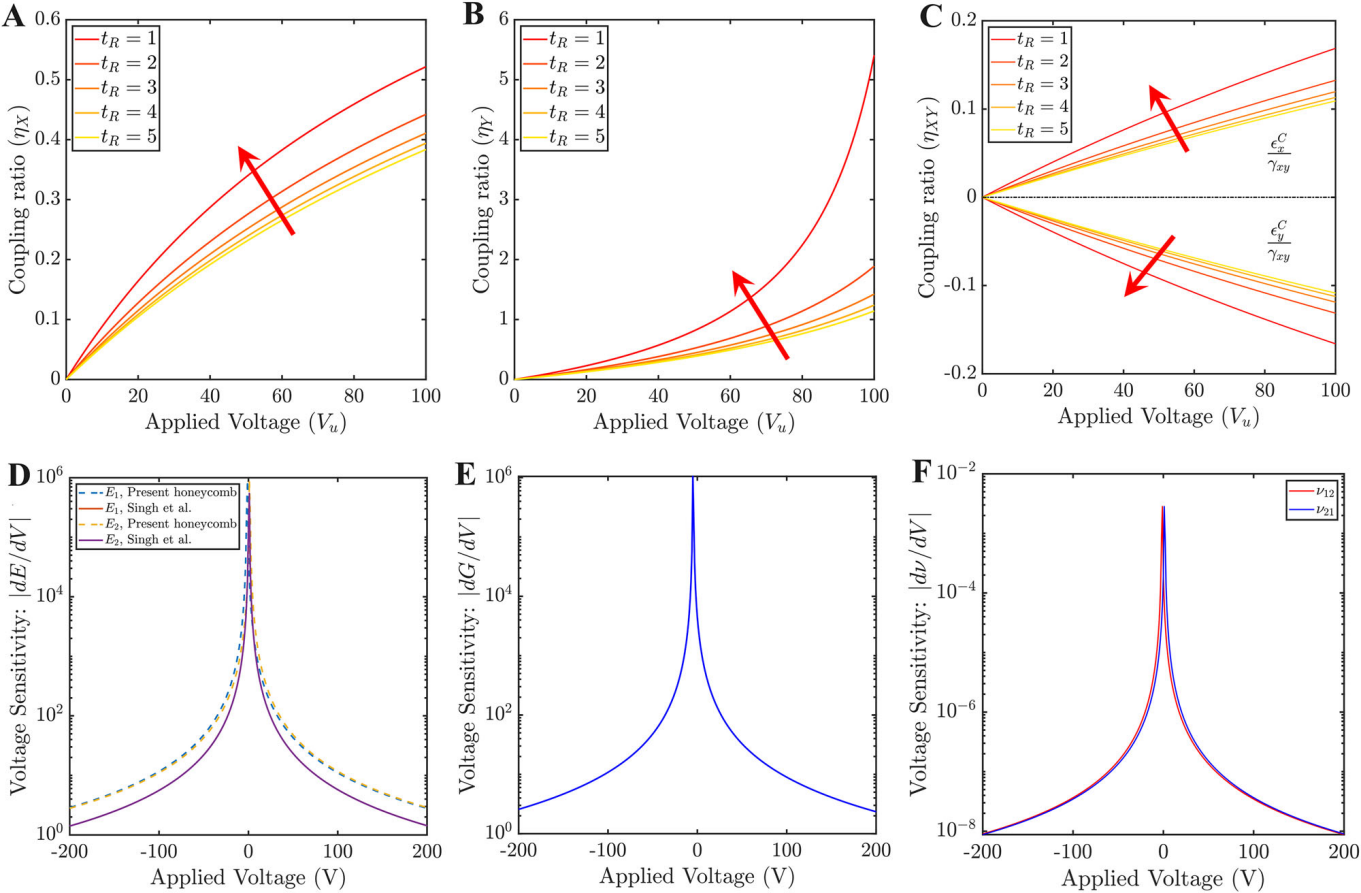
Sensitivity of cell wall architecture and external stimuli (applied voltage, *V*_*u*_) on mode coupling. **A** Variation of coupling ratio as a function of voltage applied under *X*-directional loading for different piezo thicknesses. **B** Variation of coupling ratio as a function of voltage applied under *Y*-directional loading for different piezo thicknesses. **C** Variation of coupling ratios as a function of voltage applied under shear loading for different piezo thicknesses. Note that in the above three cases, a constant stress (*σ*, *τ*) of 1000 Pa and a hybrid-voltage-ratio of 2 is maintained throughout the lattice. The red arrows here in sub-figures **A**–**C** denote an increase in piezo thicknesses. The general slope of the curves in sub-figures **A**–**C** indicates the sensitivity of piezo thickness. **D** Voltage sensitivity of Young’s moduli. For comparing it with the honeycomb lattice proposed by Singh el al.^39^, equal voltage (*V*_*u*_ = *V*_*l*_ = *V*) is applied on both the piezoelectric layers in the present lattice at constant stress (*σ* = 10 Pa). **E** Voltage sensitivity of shear modulus in the present lattice at constant shear stress, *τ*_*xy*_ = 10 Pa. **F** Voltage sensitivities of Poisson’s ratios at constant stress (*σ* = 10 Pa). The above results are presented considering *L*_R_ = 1, *θ* = 30. The voltage sensitivities for elastic moduli are given in N m^−2^ V^−1^, while those for Poisson’s ratios are given in V^−1^.

In Fig. 4A–C, the extent of normal–shear coupling is quantified by introducing a parameter *η* which is the ratio of the coupled strains (${\gamma }_{xy}^{C},{\gamma }_{xy}^{C},{\epsilon }_{x,y}^{C}$) to the corresponding strains (*ϵ*_*x*_, *ϵ*_*x*_, *γ*_*xy*_) at the applied loading direction. It can be seen that there is no coupling (*η* = 0) when piezo layers are in a passive state (*V* = 0) which is equivalent to the established theory in existing literature^48^. The coupling effect increases with applied piezo voltage as well as the thickness of the piezo layer in all the load cases. Such an increase is more rapid in the *Y*-directional loading scenario for the given voltage range (0–100 V) and its value goes beyond 1 after a certain voltage. In Fig. 4C, the normal–shear coupling about the *X*- and *Y*-direction are shown separately. It results in two different strain ratios which are opposite in sense. There exists a very minimal difference in the values along these two directions. Note that as we have shown the result at a constant hybrid-voltage ratio, the results are valid for a wide range of voltages of piezoelectric layers. At the very high voltage zone (beyond 10^3^–10^4^ V), such trends would vary to some extent due to emergent nonlinearity which is not shown here diagrammatically. In normal loading cases (*X* and *Y*), the aforementioned trend of thickness with coupling ratio will change at the high voltage zone. In Fig. 4A–C, the general slope of the curves indicates the sensitivity of piezo thickness on the corresponding coupling ratio, wherein a clear trend can be noticed that the voltage sensitivity increases with increasing piezo thickness.

#### Remarks. Uncoupled normal and shear modes: The notion of partial mode cloaking

It can be noted that the central theme of this article is to demonstrate active mode coupling between the normal and shear strains. As discussed in the preceding sections, this means both normal and shear strains would co-exist under the application of far-field normal or shear stresses. However, it is possible to achieve an unprecedented completely decoupled response, wherein only shear strain (and no normal strain) can be obtained under far-field normal stresses, and only normal strain (and no shear strain) can be obtained under far-field shear stresses.

Based on the expressions of corresponding strain fields as presented in the “Methods” section, Section S4 provides exact analytical conditions and further numerical demonstrations (refer to Fig. S10) for three different scenarios: (I) *ϵ*_*x*_ = *ϵ*_*y*_ = 0 and ${\gamma }_{XY}^{UC}\ne 0$, under applied far-field normal stress in *X*-direction, (II) *ϵ*_*x*_ = *ϵ*_*y*_ = 0 and ${\gamma }_{XY}^{UC}\ne 0$, under applied far-field normal stress in *Y*-direction, and (III) *γ*_*xy*_ = 0, ${\epsilon }_{X}^{{ {UC}}}\ne 0$ and ${\epsilon }_{Y}^{{ {UC}}}\ne 0$, under applied far-field shear stress. Note that the subscript UC is used here to represent the uncoupled strain components under the application of far-field stress in a different mode. In this context, the ability of achieving zero-strain in the direction of applied far-field stress puts forth the notion of partial mode cloaking^5^, wherein the influence of unwanted and additional far-field stress can effectively be negated as per active application-specific demands. During operational and service-life conditions, such active partial cloaking of the effect of far-field stresses or loads would be crucial to avert a range of prospective failure and serviceability constraints. The on-demand cloaking models will subsequently lead to advanced digital twins, wherein the effect of unwanted stresses in intelligent mechanical, aerospace, and biomedical structures can be identified in real-time and subsequently their influences in terms of targeted strains can be eliminated through programmed voltages for an uninterrupted mechanical performance.

#### Effective elastic properties

After exploring the strain field under applied far-field mechanical stress and external voltage, the next level derivative of interest is the voltage-dependent modulation of effective elastic properties (such as Young’s moduli, shear modulus, and Poisson’s ratios). Here computational results of the effective elastic moduli are presented following ${V}_{{{R}}}=1;{V}_{{{R}}}^{{{s}}}={V}_{{{R}}}^{3}=\frac{{V}_{{{u}}}}{{V}_{{{l}}}}$ for the sake of reducing the number of influencing parameters (refer to the closed-form expressions presented in the preceding section). First, we establish that piezoelectric lattices with cell walls made of alternating bi-material strips show higher sensitivity and magnitudes than normal non-alternating piezoelectric lattices. A comparative study of the current lattice architecture with respect to the literature^39^ is performed by obtaining the voltage sensitivity of its effective elastic moduli with respect to applied piezo-voltages (refer to Fig. 4D–F). Here only two moduli (*E*_1_ and *E*_2_) are compared with the literature as the remaining moduli (*G*_12_, *ν*_12_, and *ν*_21_) are voltage-independent in the existing literature^39^. It can be noted that overall sensitivity has improved for both moduli which makes the present lattice architecture a better choice for active property modulation. Similar trends of sensitivity are also observed in Fig. 4E and F for the case of shear modulus, *G*_12_, and Poisson’s ratios, *ν*_12_ and *ν*_21_.

**Fig. 5:**
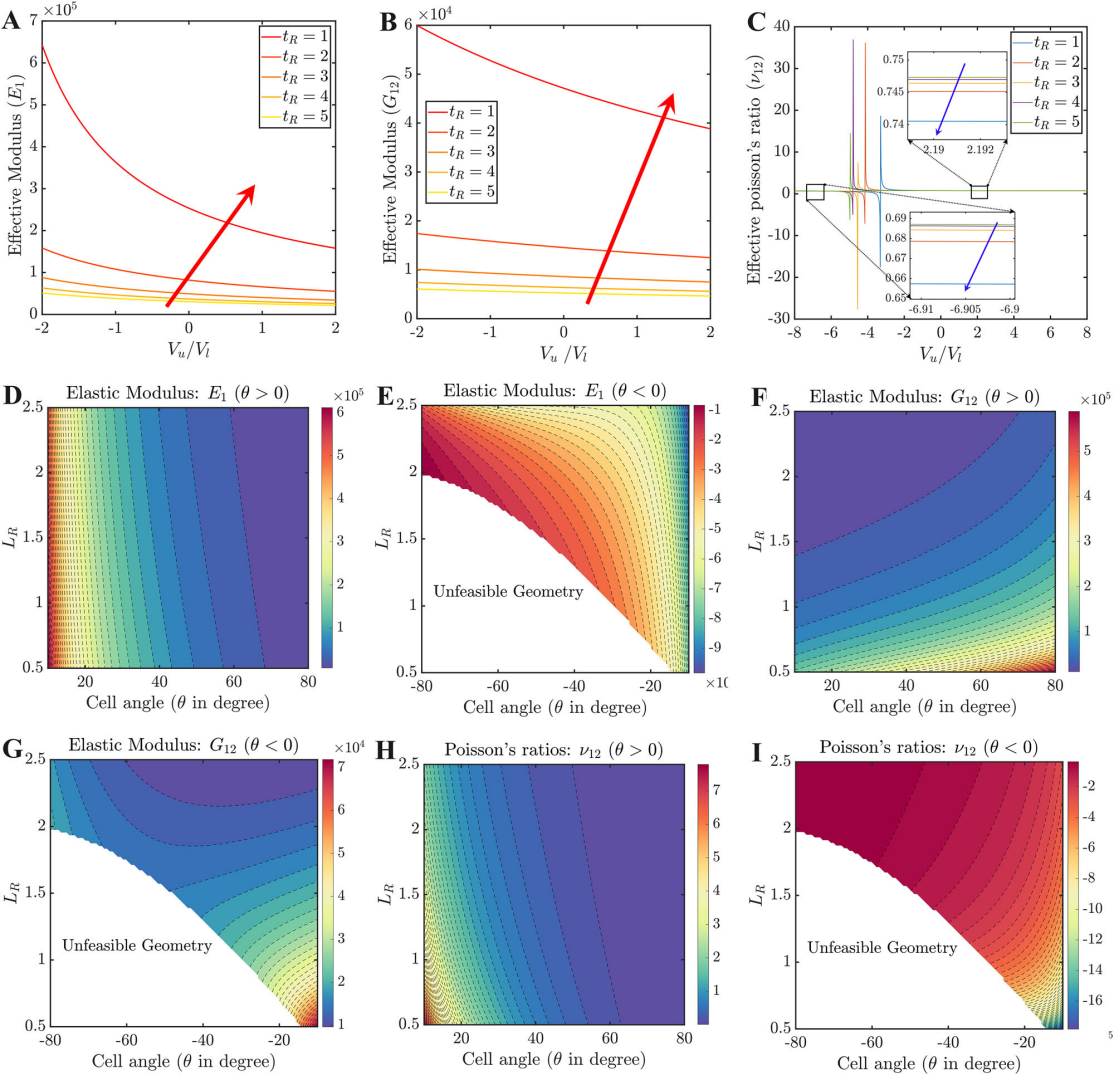
Influence of lattice architecture and external stimuli on the effective elastic properties. **A** Variation of Young’s modulus, *E*_1_ with hybrid-voltage ratio at constant mechanical normal stress (*σ*_*x*_) for different values of *t*_R_. **B** Variation of shear modulus, *G*_12_ with the hybrid-voltage ratio at constant shear stress (*τ*_*xy*_) for different values of *t*_R_. **C** Variation of Poisson’s ratio, *ν*_12_ with the hybrid-voltage ratio at constant normal stress (*σ*_*x*_) for different values of *t*_R_. **D**, **F**, **H** Contour plots of effective elastic moduli for non-auxetic lattices as a function of *L*_R_(=*h*/*L*) and cell angle (*θ*). **E**, **G**, and **I** Contour plots of effective elastic moduli for auxetic lattice as a function of *L*_R_(=*h*/*L*) and cell angle (*θ*). Here the constant stresses (*σ*_*x*_, *σ*_*y*_, *τ*_*xy*_) and the voltage, *V*_*l*_ are kept equal to 1000 Pa and 100 V, whereas the length of the inclined member and thickness of the substrate layer are kept at 60 and 0.3 mm, respectively. The arrows (red in sub-figures **A** and **B**, blue in sub-figure **C** denote an increase in piezo thicknesses. In the first three sub-figures, the following condition is conformed: *L*_R_ = 1.5 and *θ* = 30, while in the remaining sub-figures: *t*_R_ = 1 and *V*_R_ = 3. The elastic moduli are here given in N m^−2^.

Three geometrical parameters in the design space (*t*_R_, *L*_R_, and *θ*) are considered here to explore their influences on five effective constants (*E*_1_, *E*_2_, *G*_12_, *ν*_12_, and *ν*_21_) of the lattice. All the analyses are performed at a constant static mechanical stress of 1000 Pa. Figs. 5A–C and S8A, B present the nonlinear variations of elastic constants with hybrid-voltage-ratio (in the range of −2 to 2) at five different thicknesses of the piezo layers. Elastic moduli gradually reduce with the voltage ratios, whereas the same increases with piezo-thicknesses (refer to Fig. 5A–C). A similar trend is observed in other existing piezo-embedded lattice structures^38^ which indirectly affirms the validity of the present results. However, if we extend the hybrid-voltage-ratios to the higher range, the existence of a single critical ratio (${V}_{{{R}}}^{0}$) for each piezo-thickness-ratio will be observed where the magnitude of moduli becomes significantly higher. A few examples of such critical ratios are given in Table S2. The existence of these critical ratios is also shown in Figs. 5C, S9B, and S11. Such critical values are realized due to a complex coupled effect of applied voltage, far-field stress level and unit cell geometry, the physics of which is exactly captured through the proposed bottom-up analytical framework. Overall, the magnitudes of elastic moduli will decrease nonlinearly as the distance from the critical point increases and eventually, it becomes less varying. It has also been noticed that in the pre-critical zone, negative elastic modulus will prevail whereas the post-critical zone offers only positive elastic modulus (Fig. 5A–C are within the post-critical zone). In this context, it may be noted that earlier literature^39,50^ reported a few static and dynamic studies where the existence of negative elastic moduli was noted, indicating a state transition in the lattice deformation behavior.

Figures 5C and S9B show the variation for two Poisson’s ratios with hybrid-voltage-ratio (in the range of −8 to 8). In contrast to *ν*_21_, which has a direct relationship with piezo-thicknesses, *ν*_12_ has an inverse relationship with it. Similar to elastic moduli, there exist critical ratios for each thickness which are listed in Table S2. As the distance from the critical point rises, Poisson’s ratio, *ν*_12_ reduces in the post-critical zone (and it increases in the pre-critical zone) and becomes nearly saturated at a certain value. The Poisson’s ratio, *ν*_21_ shows a similar trend, except in the instance where *t*_R_ = 1. In the case of *t*_R_ = 1, an opposite trend will be observed in two zones. Note that positive Poisson ratios will prevail in both zones unless an auxetic configuration is considered.

Figures. 5D–I and S9C–F present the influence of other two geometrical parameters i.e. height-to-length ratio (*L*_R_) and cell angle (*θ*), taking *t*_R_ and voltage ratio as constant. Results are shown for auxetic (*θ* < 0) and non-auxetic lattices (*θ* > 0) separately. For auxetic lattice, the geometrical constraint i.e. ${L}_{R}+2\sin \theta > 0$ is conformed so that the vertices of the interior cell member remain untouched during the manufacturing and deformation^16,51^. In the figures, unfeasible geometry indicates the combination of those *L*_R_ and *θ* where the aforementioned geometric constraints are being violated. Note that in the present study, *θ* is taken anti-clockwise as positive. In Figs. 5D, F and S9C, it can be seen for non-auxetic lattices that (for a particular height-ratio) effective *E*_2_ and *G*_12_ increase with cell angles whereas an opposite trend is observed in the case of *E*_1_. The magnitudes of the moduli are smaller than that of the lattice’s intrinsic material properties. This kind of elastic moduli trend with cell angles is supported by existing literature. For instance, utilizing the volume-average methodology and the energy method, Qiu et al.^52^ reported a similar pattern in their non-auxetic normal honeycombs. Figs. 5E, G and S9D present moduli in an auxetic scenario where only *E*_1_ exhibits a negative Young’s modulus. It is observed that the trends of *E*_1_ and *E*_2_ with cell angle are quite similar (magnitude-wise) to those of non-auxetic cases. While *G*_12_ increases with the magnitudes of cell-angle (∣*θ*∣) at higher height ratios, and the trend reverses at lower height ratios. Regarding height ratios, in both auxetic and non-auxetic lattices, an overall increment and a decrement with *L*_R_ are observed in *E*_2_ and *G*_12_, respectively. However, the diminishing tendency of *E*_1_ with height-ratio in non-axuetic lattices is reversed in auxetic lattices.

The contour plots for Poisson’s ratios (*ν*_12_ and *ν*_21_) are depicted in Figs. 5H, I and S9E, F for both the lattice architectures. It has been noted that negative in-plane Poisson’s ratio predominates in auxetic lattices, and vice-versa for the non-auxetic configurations. Overall the magnitude of *ν*_12_ gradually declines with the cell angle in both architectures, whereas the trend becomes the opposite in *ν*_21_. Compared to elastic moduli, the effect of cell angle on Poisson’s ratios is less dominant. Moreover, *ν*_12_ in both lattices is found to be in direct relation with the height ratio (*L*_R_), but the relation becomes inverse in the case of *ν*_21_.

### Summary and perspective

The numerical results presented in the preceding section demonstrate active and programmable normal–shear mode coupling based on the bi-level optimally mounted metamaterial architecture. This leads to achieving on-demand controllable coupled shear deformation along with normal deformations under the application of far-field normal stress, and vice-versa. It is demonstrated that the normal and shear modes can be completely uncoupled under specific conditions of applied far-field stress and the voltages, leading to a notion of on-demand partial mode cloaking. Further, we show that the proposed active metamaterial architecture shows programmable elastic moduli (two Young’s moduli and shear modulus) and Poisson’s ratios as a function of applied external voltage, wherein a single material can be made stiff or flexible in real-time by orders of magnitude.

Through the proposed lattice metamaterial, we show that active modulation of elastic properties and normal–shear coupling can be realized through simple symmetric unit cell architectures, leading to improved manufacturing flexibility and efficiency. Here the discussion regarding ease of manufacturing is not limited to additive manufacturing, where achieving complex lattice geometries is relatively more straightforward, but exorbitantly time-consuming in many cases. On the contrary, traditional subtractive manufacturing methods are still widely used for making hexagonal lattices at industrial scales. In such methods, having a simple geometry for the lattice leads to efficient manufacturing processes. The active components can be attached to the beam substrates as patches, or realized through coating. However, with the recent progress in additive manufacturing, the proposed lattices can be envisaged to be additively manufactured along with the active components wherein the expense, manufacturing duration, mechanical strength, and durability factors should be investigated further.

Concerning the proposed multi-physical metamaterial, we have developed a high-fidelity bottom-up beam-based computational framework that is validated independently at multiple length scales for ascertaining prediction accuracy. This includes validation of the optimally mounted piezoelectric elementary beams with appropriate boundary conditions to conform unit cell level periodicity and subsequent lattice-level validation considering unit cells. The bottom-up beam-based analytical framework for analyzing the proposed metamaterial is validated with a unit cell-based assembled direct stiffness method (matrix approach) and finite element-based approach considering active elements. In addition, we present an exact lattice-level analytical validation with respect to available literature considering the special case of passive conventional lattices. Such extensive validation considering elementary beam level and lattice level and involving multiple approaches would garner adequate confidence in the computational outcomes.

The active and programmable control on the normal–shear mode coupling and the effective stiffness along with the capability to modulate the deformation field in normal and shear directions could be exploited in applications where lightweight, on-demand specific stiffness, as well as active shape-morphing properties, are demanded such as morphing aerofoil and wind turbine blades, MEMS devices, robotic control and gripper applications. For example, in order to achieve aerodynamically adaptive optimal flight performance, on-demand span, chord, and sweep morphing in aerofoils can be achieved readily through active in-plane deformation, while camber morphing could be attained through programmable differential deformation of multi-layer metamaterials (refer to Fig. 1G)^53^. Further, the property of active and on-demand seamless transition of the proposed metamaterial between stiff and flexible characteristics can be exploited during operational conditions. In conventional materials, high stiffness demands high density as well, leading to additional weight. However, higher stiffness (both in normal and shear modes), on an on-demand basis, can be achieved in the proposed metamaterial actively through applied voltage without any additional weight. For example, under flying conditions, the aircraft wings would require higher stiffness due to increased aerodynamic loads. Such demands can be actively met using the proposed metamaterial by supplying algorithmically controlled voltage. In addition, on-demand flexibility in the metamaterial can also be achieved to avert resonance and improve energy absorption capability under impact loading. Further, during operational and service-life conditions, the notion of active partial cloaking concerning the effect of far-field stresses or loads would be crucial to avert a range of prospective failure and serviceability constraints. Exploitation of the full potential of the proposed metamaterial would lead to substantially reduced fuel consumption, reduced carbon footprint, enhanced economic benefits, and sustainability^54^ during manufacturing and operational conditions.

In the field of soft robotics, the concept of achieving movement through deformation mechanisms is often referred to as deformation-based locomotion, leading to navigating complex and dynamic environments, adapting to various tasks, and interacting safely with humans and other objects. This approach involves designing and utilizing soft, flexible materials and structures that can change their shape and deform to generate motion, rather than relying on traditional rigid components and mechanisms^55,56^. Such locomotion in the proposed piezo-embedded honeycomb metamaterial with normal–shear coupling can be readily achieved and temporally controlled using external voltages. It can be accomplished through repetitive and temporally programmed voltage-induced deformation stages as shown in Fig. 1F involving sequential loading and unloading, wherein the coupled shear deformation plays a crucial role (refer to the supplementary movies M1 and M2 showing a 3D rendered view and 2D view, respectively). The locomotion can be realized using two frameworks: (1) sequential loading and unloading along the normal vertical direction while keeping a constant electrical voltage and (2) sequentially applying voltage and then turning it off under a given value of loading. The spring in the schematic design of supplementary movie M1 is attached to allow some normal deformation while realizing shear deformation under the application of sequential normal load or voltage. However, it may be possible to achieve completely uncoupled shear and normal modes as discussed in the remarks (refer to the normal–shear mode coupling section), and the required spring stiffness can be designed commensurate to the extent of normal–shear coupling. Note that the upper and lower boundary conditions assume a vital role here, through which temporally programmed normal deformation is applied for the locomotion. As illustrated in Fig. 1F, the robotic metamaterial can move from the initial to the final position by application of sequential vertical far-field stresses (or sequentially applied voltage) as discussed above and exploiting the resulting shear deformation. In each of the stages, after the application of vertical stresses (or voltage), one of the horizontal edges needs to be restrained with the corresponding support and the opposite edge should be free to move (refer to the supplementary movie M1). This will enable the movement of the robotic metamaterial in a particular direction depending on the polarity of applied voltage and (/or) far-field vertical deformation (or stress). Interestingly, if the applied voltage is zero, the proposed metamaterial would not have any locomotion. Thus, it is possible to have a load-bearing stationary metamaterial working on a particular spot and then have locomotion to move it to a different spot for performing further load-bearing operations in the new location. Such on-demand robotic motion and load-bearing performance are proposed for the first time in this work.

As the first-ever work in this field, we have confined our analytical formulation to the linear small deformation regime. The aspect of active mode coupling can be readily extended to the nonlinear large-deformation regime by adopting nonlinear deformation mechanics at the beam level and including incremental change in unit cell geometry^28^. However, it becomes qualitatively clear that the active normal–shear mode coupling can also be realized in the nonlinear regime, albeit the current formulation needs further modification for a quantifiable measure. In such scenarios, relatively more flexible versions of piezoelectric patches can be used (such as piezo composites) following a similar metamaterial architecture. For the soft robotic applications proposed here, the rate of motion would be smaller per actuation cycle under the small deformation assumption. However, based on the above discussion on active normal–shear mode coupling under large deformation, the same metamaterial framework can be adopted for incorporating larger deformation per cycle to achieve improved robotic motion based on the nonlinear analysis of the unit cells.

A direct derivative of normal–shear active mode coupling proposed here is further coupling among normal–torsional-bending modes, as explained in Fig. 1E. We propose an axis-symmetric concentric cylindrical shape with the piezo-embedded honeycombs as curved walls. Comparing such architected cylindrical metastructure to typical solid circular beams, when a torsional load is applied at the end of the beam, any small rectangular element on the surface exhibits a shear strain along with normal strain, as depicted in the figure. Conversely, under the application of normal axial force to the metastructure, there will be shear and normal deformations. Such mixed-mode strains would eventually result in a coupling among normal and torsional modes of deformations (under externally applied axial or torsional forces), along with the feature of active programmability due to the presence of piezoelectric composite bi-level honeycomb-like architecture. Note that the bending deformation mode can also be coupled along with normal and torsional deformation therein by introducing variable application of voltage along the cross-section of the proposed beam. In summary, we show that active and programmable normal–shear, normal–torsion and normal–torsion-bending mode couplings are feasible through the proposed bi-level metamaterial architecture, which can find applications in a range of actuators, sensors, and controllers^57^. For example in tunable mechanical filters, the programmable smart metamaterials can be exploited for signal processing and noise reduction. By adjusting the shear-axial coupling properties, these materials can selectively filter out specific mechanical frequencies or vibrations. The coupling between different modes can result in the conversion of twist or shear modes of wave propagation to longitudinal axial deformation modes, or vice-versa, depending on the intended functionalities of waveguides. More interestingly, the range of such filtration and conversion can be actively tuned based on functional online demands.

In the proposed bi-level architected metamaterial, the converse piezoelectric effect is exploited for obtaining programmable effective stiffness and active coupling in the deformation fields. However, the same metamaterial architecture can turn into a broadband energy harvesting device if the direct piezoelectric effect is utilized. Multiple modes of vibration including normal, shear, torsional, and bending modes can simultaneously be exploited there for enhanced power generation, but using just a single mode of piezoelectric deformation. Further, the effect of electromechanical coupling^58–60^ could be incorporated into the computational framework for a more accurate and efficient design of the metamaterial architectures.

## Conclusions

A bi-level multi-physically architected active class of lattice metamaterial is proposed in this article to achieve on-demand property modulation in real-time with greater external stimuli sensitivity. We break the traditional realm of the material constitutive behavior of uncoupled normal and shear modes through the concept of stimuli-responsive deformation physics. Shear strain can be achieved under far-field normal stresses, and vice-versa, wherein both shear and normal strains co-exist. It is further possible to achieve an unprecedented mode-wise completely decoupled stress-strain constitutive behavior, wherein only shear strain (and no normal strain) can be obtained under far-field normal stresses, and vice-versa. More interestingly, this is achieved in conventional symmetric lattice geometries through an intuitive physics-informed mounting of electro-active elements. The notion of active partial cloaking concerning the effect of far-field complex stresses is established under specific conditions of applied voltage, leading to the prospect of averting a range of failure and serviceability constraints.

The computational results demonstrate an unprecedented programmable voltage-dependent normal–shear mode coupling for critically exploitable temporally periodic or aperiodic, on-demand, and tunable mechanical responses. Further, the proposed active metamaterial architecture shows programmable elastic moduli (Young’s moduli and shear modulus) and Poisson’s ratios as a function of applied external voltage, wherein a single material can be made stiff or flexible depending on application-specific operational demands by orders of magnitude along with state transition. The manufacturing flexibility in terms of symmetric lattice geometry, along with actively tunable normal–shear mode coupling and programmable stiffness modulation capability in the new class of metamaterials would lead to real-time control of mechanical responses for temporal programming in a wide range of advanced mechanical applications, including morphing and transformable geometries, locomotion in soft robotics, embedded actuators, enhanced multi-modal energy harvesting, vibration, and wave propagation control.

## Supplementary information

Supplementary Information

Description of Additional Supplementary Files

Supplementary Data 1

## Data Availability

All data sets used to generate the results are available in the main paper and the supplementary material. Further details could be obtained from the corresponding author upon reasonable request.
