# Supplementary material for: Active heterogeneous mode coupling in bi-level multi-physically architected metamaterials for temporal, on-demand and tunable programming
Source: Commun Eng. 2025 Jun 7;4:103. doi: 10.1038/s44172-025-00420-7 (PMC12145453; doi:10.1038/s44172-025-00420-7)
Supplement: Supplementary file 1 — Supplementary Information [file 44172_2025_420_MOESM1_ESM.pdf]

Supplementary material: Active heterogeneous mode coupling in bi-level multi-physically architected metamaterials for temporal, on-demand and tunable programming

S. Mondal[a], T. Mukhopadhyay[a,*], S. Naskar[a,*]

[a]*Faculty of Engineering and Physical Sciences, University of Southampton, Southampton, UK*

Contents

S1 List of symbols **2**

S2 Analytical formulation concerning beam-level deformation physics **3**

S2.1 Axial deformation 3

S2.2 Transverse deformation 3

S3 Derivation for lattice-level elastic stress and strain fields **5**

S3.1 Normal uni-axial far-field stress in X-direction 5

S3.2 Normal uni-axial far-field stress in Y-direction 8

S3.3 Far-field shear stress 9

S4 Uncoupled normal and shear modes: Notion of partial mode cloaking **12**

S5 Additional numerical results **15**

S6 Coefficients of E_1 and ν_{12} **23**

S7 Coefficients of E_2 and ν_{21} **24**

S8 Coefficients of G_{12} **26**

*Email address: T.Mukhopadhyay@soton.ac.uk(TM), S.Naskar@soton.ac.uk (SN)

S1. List of symbols

t, θ	Cell wall's thickness and inclination angle
L, h	Length of slant and vertical members
w	Out-of-plane dimension of the honeycomb panel
t_p	Thickness of piezoelectric layer
t_s	Thickness of non-piezoelectric substrate layer
s_{11}^E	Elastic compliance of the piezoelectric element
s_{11}^m	Elastic compliance of the non-piezoelectric substrate layer
d_{31}	Piezoelectric coupling coefficient
M_{V_s}	Equivalent moment of piezo layer of slant members.
M_{V_3}	Equivalent moment of piezo layer of vertical members.
F_{V_s}	Equivalent axial force of piezo layer of slant members.
F_{V_3}	Equivalent axial force of piezo layer of vertical members.
V_u^s	Applied voltage on upper piezo layer of slant members
V_l^s	Applied voltage on bottom piezo layer of slant members
V_u^3	Applied voltage on upper piezo layer of vertical members
V_l^3	Applied voltage on lower piezo layer of vertical members
V_u	Common applied voltages on upper piezo layers of the members.
V_l	Common applied voltages on lower piezo layers of the members.
σ_x, σ_Y	External mechanical uniaxial stress along X- and Y-direction
τXY	Remote shear stress
ϵ_X, ϵ_Y	Normal strains under normal loading
γ_{XY}^C	Coupled shear strain under normal loading
γ_{XY}	Shear strain under shear loading
ϵ_X^C, ϵ_Y^C	Coupled normal strains under shear loading.
E_1, E_2, G_{12}	Effective Young's module and shear modulus
μ_{12}, μ_{21}	Effective Poisson's ratios
E_p	Young's modulus of piezoelectric layer
E_m	Young's modulus of non-piezoelectric substrate layer
η_X, η_Y, η_{XY}	Coupling ratios
t, t_1, t_2	Free variables

S2. Analytical formulation concerning beam-level deformation physics

S2.1. Axial deformation

To find out axial deformation, a uniform strain variation along the cross-section of the beam is assumed where the axial strain, ϵ_x can be given as follows

$$\epsilon_x = \frac{du}{dx} \tag{S1}$$

Here, u denotes the deflection along beam's length direction (x axis). Substituting ϵ_x for individual layers in equation 1a, the following stress equations can be obtained

$$\sigma_x^u = \frac{1}{s_{11}^E}\left(\frac{du}{dx}\right) + d_{31}\frac{1}{s_{11}^E}\left(\frac{V_u}{t_p}\right) \tag{S2a}$$

$$\sigma_x^m = \frac{1}{s_{11}^m}\left(\frac{du}{dx}\right) \tag{S2b}$$

$$\sigma_x^l = \frac{1}{s_{11}^E}\left(\frac{du}{dx}\right) - d_{31}\frac{1}{s_{11}^E}\left(\frac{V_l}{t_p}\right) \tag{S2c}$$

Now, taking the total internal force across thickness, $N(x)$ and equating it to the total external axial force, F_x, the following equation is obtained

$$N(x) = \int_0^w \int_{\frac{t_m}{2}}^{t_p+\frac{t_m}{2}} \sigma_x^u dydz + \int_0^w \int_{-\frac{t_m}{2}}^{\frac{t_m}{2}} \sigma_x^m dydz + \int_0^w \int_{-(t_p+\frac{t_m}{2})}^{-\frac{t_m}{2}} \sigma_x^l dydz = F_x \tag{S3}$$

Solving equation S3 for $\epsilon_x(=\frac{du}{dx})$, the following expression is obtained.

$$\frac{du}{dx} = \frac{s_{11}^m\,\left(F_x\,s_{11}^E + V_l\,d_{31}\,w - V_u\,d_{31}\,w\right)}{w\,\left(s_{11}^E\,t_m + 2\,s_{11}^m\,t_p\right)} \tag{S4}$$

Axial deformation of the cantilever beam of length $L/2$ can now be obtained from equation S4 as

$$u_{L/2} = \int_0^{\frac{L}{2}} \frac{du}{dx}dx \tag{S5}$$

The final closed-form expression of total axial deformation of the entire both-end rotationally restrained beam of length L

$$\delta_x = 2u_{L/2} = \frac{L\,s_{11}^m\,\left(F_x\,s_{11}^E + V_l\,d_{31}\,w - V_u\,d_{31}\,w\right)}{w\,s_e^T} \tag{S6}$$

where $s_e^T = s_{11}^E t_m + 2s_{11}^m t_p$.

S2.2. Transverse deformation

To find out transverse deformation, a linear strain variation along the cross-section of the beam is assumed where the axial (extensional) strain, ϵ_x can be given as follows

$$\epsilon_x = \kappa z = z\frac{d^2v}{dx^2} \tag{S7}$$

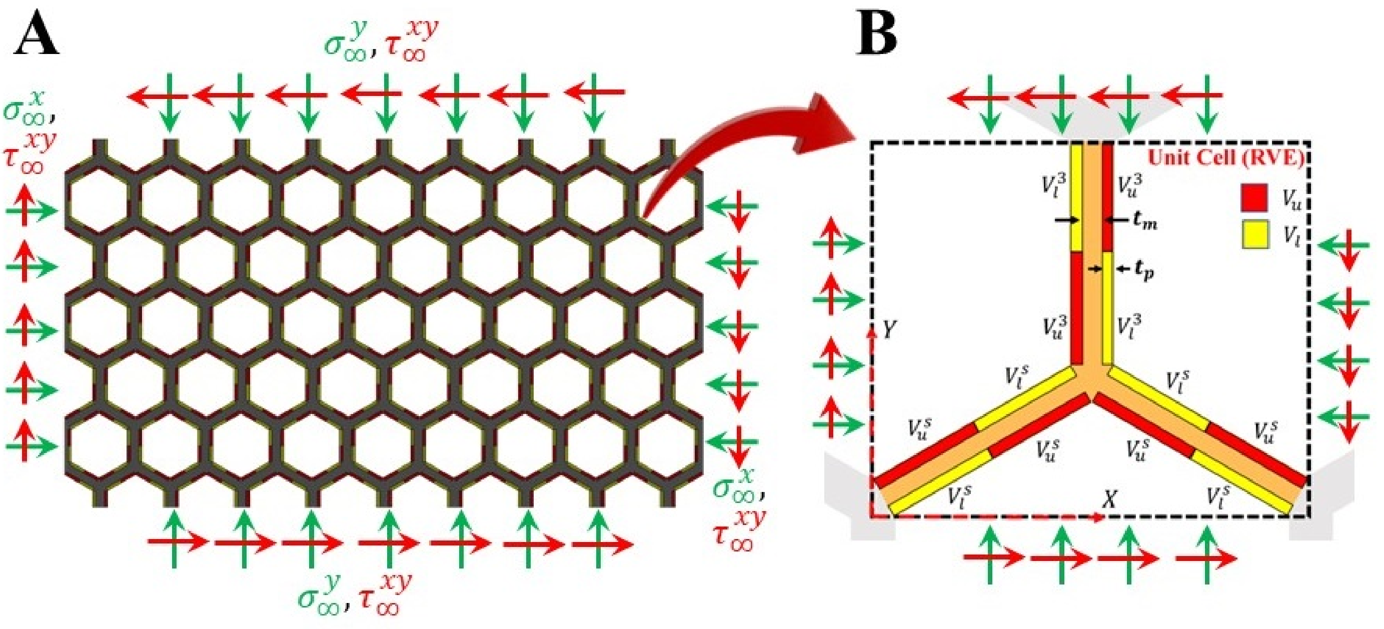

Fig. S1: Far-field and unit cell level stresses. **(A)** Far-field normal and shear stresses applied to the edges of a lattice. **(B)** Equivalent normal and shear stress applied to a unit cell. For calculating the elastic moduli individually, the normal stresses in longitudinal and transverse directions, and shear stress are applied accordingly one at a time.

Here, v denotes the deflection along beam's transverse direction (z axis). Substituting ϵ_x for individual layers in equation 1a, the following stress equations can be obtained

$$\sigma_x^u = \frac{1}{s_{11}^E}\left(z\frac{d^2v}{dx^2}\right) + d_{31}\frac{1}{s_{11}^E}\left(\frac{V_u}{t_p}\right) \tag{S8a}$$

$$\sigma_x^m = \frac{1}{s_{11}^m}\left(z\frac{d^2v}{dx^2}\right) \tag{S8b}$$

$$\sigma_x^l = \frac{1}{s_{11}^E}\left(z\frac{d^2v}{dx^2}\right) - d_{31}\frac{1}{s_{11}^E}\left(\frac{V_l}{t_p}\right) \tag{S8c}$$

Evaluating the total internal bending moment across the beam thickness, $M(x)$ at any point x along the longitudinal direction and equating it to the total moment due to the external transverse force, F_y, the following equation can be obtained.

$$M(x) = \int_0^w \int_{\frac{t_m}{2}}^{t_p+\frac{t_m}{2}} \sigma_x^u z dy dz + \int_0^w \int_{-\frac{t_m}{2}}^{\frac{t_m}{2}} \sigma_x^m z dy dz + \int_0^w \int_{-(t_p+\frac{t_m}{2})}^{-\frac{t_m}{2}} \sigma_x^l z dy dz = F_y\left(\frac{L}{2} - x\right) \tag{S9}$$

Solving equation S9 for curvature $\kappa(= \frac{d^2v}{dx^2})$, the following expression is obtained.

$$\frac{d^2v}{dx^2} = \frac{F_y\,\left(\frac{L}{2} - x\right)}{\frac{{t_m}^3\,w}{12\,s_{11}^m} + \frac{t_p\,w\,(3\,{t_m}^2+6\,t_m\,t_p+4\,{t_p}^2)}{6\,s_{11}^E}} \tag{S10}$$

Transverse deformation of a cantilever half beam of length $L/2$ can now be obtained from equation S10 as follows

$$v_{L/2} = \int_0^{\frac{L}{2}} \int_0^{\frac{L}{2}} \frac{d^2v}{dx^2} dx dx \tag{S11}$$

The final closed-form expression of total transverse deformation of the full-length (i.e. length L) beam with both-end rotationally restrained supports is given as

$$\begin{aligned} \delta_z &= 2v_{L/2} \\ &= \frac{L^3\, s_{11}^E\, s_{11}^m}{w\, s_e^t}\, F_y - \frac{3L^2\, d_{31}\, s_{11}^m\, (t_m + t_p)}{2s_e^t}\, V_u - \frac{3L^2\, d_{31}\, s_{11}^m\, (t_m + t_p)}{2s_e^t}\, V_l \end{aligned} \tag{S12}$$

where $s_e^t = s_{11}^E\, {t_m}^3 + 6\, s_{11}^m\, {t_m}^2\, t_p + 12\, s_{11}^m\, t_m\, {t_p}^2 + 8\, s_{11}^m\, {t_p}^3$. The other geometric parameters are explained in the schematic figures of the unit cells (refer to figure 1(B-D)).

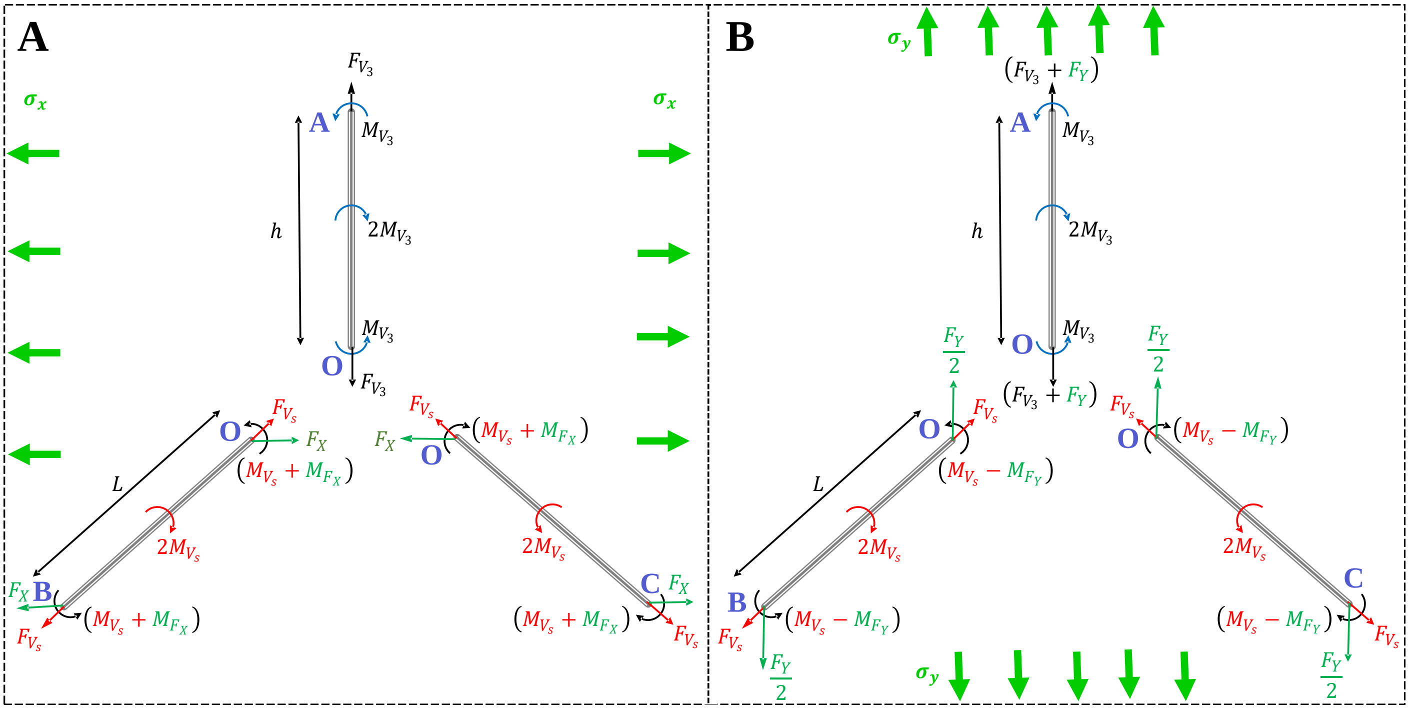

Fig. S2: Free body diagram of a unit cell under normal far-field stresses. (A) Uniaxial stress σ_x. **(B)** Uniaxial stress σ_y. Here contributions of piezoelectric loading are shown in terms of its equivalent nodal forces and moments. As the formulation has been derived considering two different voltages for vertical and slant cell members, equivalent piezo-loadings are shown different for these two members here. The joints in the unit cell (A, B, C and O) are indicated using blue color to differentiate these from subfigure numbers.

S3. Derivation for lattice-level elastic stress and strain fields

S3.1. Normal uni-axial far-field stress in X-direction

As shown in Figure 1(C), the voltages applied on vertical members of the unit cell are V_u^3 and V_l^3, whereas that of two slant members are V_u^s and V_l^s. Under such electrical loading, in each member of the unit cell, there will be piezoelectric axial as well as bending deformations. External mechanical uniaxial stress (σ_x) is applied to the honeycomb in X-direction. In the present formulation, a linear-elastic deformation under a small strain assumption is used for the honeycomb.

Figure S2(A) shows the free-body diagram of the unit cell considering its static equilibrium. Applied stress σ_x (at global scale) is transferred to the unit cell as nodal force F_X applied on nodes B and C

of the inclined cell walls having inclination angle θ. The influence of applied voltages is shown in the diagram with the help of its equivalent moment-force terms (M_V and F_V). The force F_x can be given as

$$F_X = \sigma_x(h + L\sin\theta)w \tag{S13}$$

The vertical cell wall (AO) will not be effected by F_X as it is acting symmetrically w.r.t AO. Rather, its deformation will be solely dependent on the applied voltages (V_u^3 and V_l^3). Therefore, the bending deflection in X-direction of the end A w.r.t. the end O can be given as

$$\delta_{AO}^b = \left(-\frac{3h^2\, d_{31}\, s_{11}^m\, (t_m + t_p)}{2s_e^t}\right)(V_u^3 + V_l^3) \tag{S14}$$

where $s_e^t = s_{11}^E\, t_m{}^3 + 6\, s_{11}^m\, t_m{}^2\, t_p + 12\, s_{11}^m\, t_m\, t_p{}^2 + 8\, s_{11}^m\, t_p{}^3$. Similarly, the axial deformation in Y-direction can be given as

$$\delta_{AO}^a = \frac{h\, s_{11}^m\, (V_l^3\, d_{31} - V_u^3\, d_{31})}{(s_{11}^E\, t_m + 2\, s_{11}^m\, t_p)} \tag{S15}$$

The deformation of slant cell walls solely depends on applied external mechanical stress and voltages on the piezoelectric layers of the slant members. Cumulative effect of bending moment M_{F_X} due to applied load F_X and the bending moment M_{V_s} due to applied voltage (V_s) will tend to bend the slant member BO and CO in a manner where end nodes will be rotationally intact after deformation (due to the symmetry of the unit cell). Here M_{F_X} can be derived considering the equilibrium of member BO (or CO) which is as follows

$$M_{F_X} = \frac{F_X L\sin\theta}{2} \tag{S16}$$

In member BO, the axial load along BO is $F_X\cos\theta + F_{V_s}$ and the transverse load normal to BO is $F_X\sin\theta$. From equation S12 and S6, bending (δ_{BO}^b) and axial (δ_{BO}^a) deformations w.r.t joint O can be obtained

$$\delta_{BO}^a = \frac{L\, s_{11}^m\, \left(F_X\cos\theta\, s_{11}^E + V_l^s\, d_{31}\, w - V_u^s\, d_{31}\, w\right)}{w\, (s_{11}^E\, t_m + 2\, s_{11}^m\, t_p)} \tag{S17}$$

$$\delta_{BO}^b = \frac{L^3\, s_{11}^E\, s_{11}^m}{w\, s_e^t} F_X\sin\theta - \left(\frac{3L^2\, d_{31}\, s_{11}^m\, (t_m + t_p)}{2s_e^t}\right)(V_u^s + V_l^s) \tag{S18}$$

Total normal strain in X-direction can be written as

$$\epsilon_X = \frac{\delta_{BO}^b\sin\theta + \delta_{BO}^a\cos\theta}{L\cos\theta} \tag{S19}$$

Total shear strain for the present loading case can be given as

$$\gamma_{XY}^C = \frac{\delta_{AO}^b}{h + L\sin\theta} \tag{S20}$$

Remarks. Effective elastic properties, E_1 and ν_{12}

To quantify effective elastic properties for the present loading condition, effective elastic modulus (E_1) parallel to X can be defined as

$$E_1 = \frac{\sigma_x}{\epsilon_X} = \frac{\lambda_1^{E_1}}{\lambda_2^{E_1} + \lambda_3^{E_1}\left(\frac{V_u^s}{\sigma_x}\right)} \tag{S21}$$

The effective Poisson's ratio, ν_{12} can also be defined by taking the negative ratio of strain perpendicular to and parallel to the present loading direction. The net strain ϵ_Y (due to the Poisson's effect) can be given as

$$\epsilon_Y = -\frac{-\delta_{BO}^a \sin\theta + \delta_{BO}^b \cos\theta - \delta_{AO}^a}{h + L\sin\theta} \tag{S22}$$

Therefore, the effective Poisson's ratio can be obtained as

$$\nu_{12} = -\frac{\epsilon_Y}{\epsilon_X} = \frac{\beta_1^{\nu_{12}} + \beta_2^{\nu_{12}}\left(\frac{V_u^s}{\sigma_x}\right)}{\beta_3^{\nu_{12}} + \beta_4^{\nu_{12}}\left(\frac{V_u^s}{\sigma_x}\right)} \tag{S23}$$

Here the expressions of the coefficients ($\lambda_i^{E_1}$ and $\beta_i^{\nu_{12}}$) are given in section S6 of supplementary material where the following ratios have been used: $L_R = \frac{h}{L}, t_R = \frac{t_m}{t_p}, s_{11}^R = \frac{s_{11}^m}{s_{11}^E}, V_R = \frac{V_u^s}{V_u^3}, V_R^s = \frac{V_u^s}{V_l^s}, V_R^3 = \frac{V_u^3}{V_l^3}$. From the expressions as functions of σ_x and V_u^s in equations S21 and S23, it is evident that the voltage applied on the vertical member has an influence on the effective Poission's ratio of the lattice, whereas the effective Young's modulus is solely controllable by voltages on the slant members.

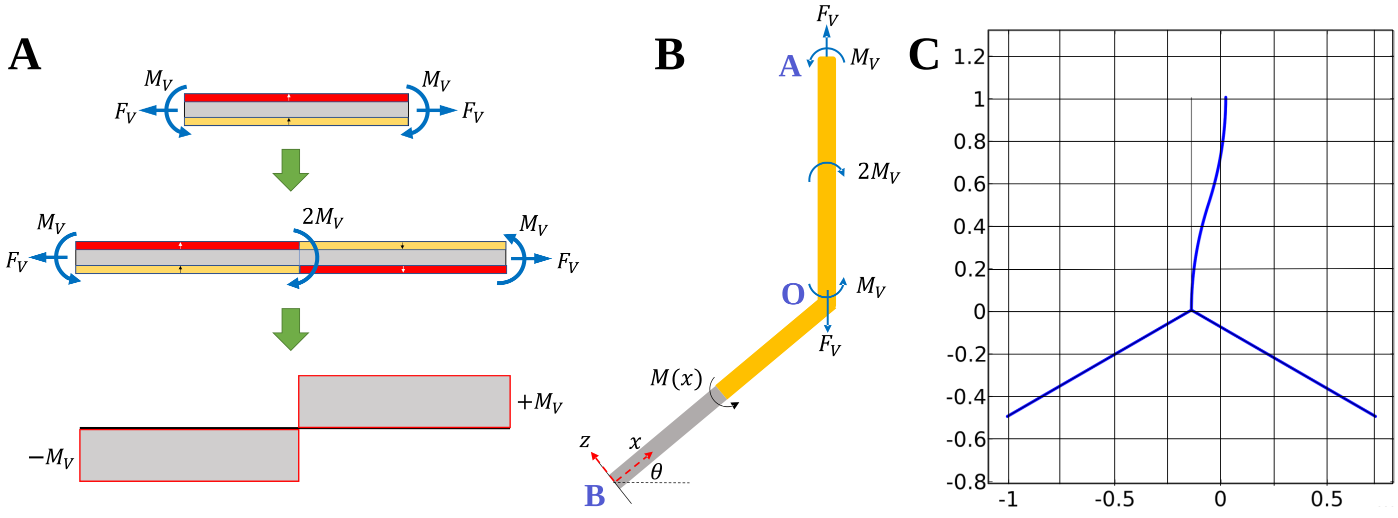

Fig. S3: Visualization of net zero joint-rotation due to piezoelectric effect on the vertical members. (A) Representation of piezoelectric loading in normal bimorph beam element (two-noded) and present hybrid piezo-beam element (three noded) in terms of its equivalent moment (M_V) and axial force (F_V). **(B)** Example of a simple beam-bent problem with vertical member under axial loads and equivalent moments ($M(x)$ represents the internal bending moment at an arbitrary cut of the inclined member). **(C)** COMSOL validation of zero-rotation at the intersection joint of the unit cell due to equivalent piezoelectric effect on the vertical member.

S3.2. Normal uni-axial far-field stress in Y-direction

Normal far-field stress σ_Y is applied to the honeycomb lattice along its global Y-direction (refer to Figure S2(B)). The stress can be written in terms of nodal force F_Y applied at the nodes of the unit cell as

$$F_Y = 2\sigma_Y L w \cos\theta \tag{S24}$$

This F_Y will not only tend to bend the slant members (BO and CO), but also elongate the vertical member (AO) unlike the case of loading in X direction. However, deformation cases associated with piezoelectric voltages will be the same as the case of loading in X direction. The applied mechanical load F_Y on AO will be equally distributed into slant members (BO and CO) due to their equal mechanical stiffness. Using equilibrium of the slant members, the bending moment M_{F_Y} can be obtained as follows

$$M_{F_Y} = \frac{F_Y L \cos\theta}{4} \tag{S25}$$

The axial deformation (δ^a_{AO}) and bending deformation (δ^b_{AO}) at A with respect to the joint O can can be obtained from equation S12 and S6 as

$$\delta^a_{AO} = \frac{h\, s^m_{11}\, \left(F_Y\, s^E_{11} + V^3_l\, d_{31}\, w - V^3_u\, d_{31}\, w\right)}{w\, \left(s^E_{11}\, t_m + 2\, s^m_{11}\, t_p\right)} \tag{S26}$$

$$\delta^b_{AO} = \left(-\frac{3h^2\, d_{31}\, s^m_{11}\, (t_m + t_p)}{2s^t_e}\right) (V^3_u + V^3_l) \tag{S27}$$

Now coming to slant member BO, the axial load acting along BO is $(F_Y/2)\sin\theta + F_{V_s}$ and the transverse load on BO is $(-F_Y/2)\cos\theta$. From equation S12 and S6, bending (δ^b_{BO}) and axial (δ^a_{BO}) deformations with respect to joint O can be derived as

$$\delta^a_{BO} = \frac{L\, s^m_{11}\, \left(F_Y \sin\theta\, s^E_{11} + 2V^s_l\, d_{31}\, w - 2V^s_u\, d_{31}\, w\right)}{2w\, \left(s^E_{11}\, t_m + 2\, s^m_{11}\, t_p\right)} \tag{S28}$$

$$-\delta^b_{BO} = \frac{L^3\, s^E_{11}\, s^m_{11}}{2w s^t_e} (-F_Y \cos\theta) - \left(\frac{3L^2\, d_{31}\, s^m_{11}\, (t_m + t_p)}{2s^t_e}\right) (V^s_u + V^s_v) \tag{S29}$$

Total normal strain in Y-direction can be written as

$$\epsilon_Y = \frac{\delta^a_{AO} + \delta^b_{BO}\cos\theta + \delta^a_{BO}\sin\theta}{h + L\sin\theta} \tag{S30}$$

Total shear strain resulting from bending deformation of the vertical member can be given as

$$\gamma^C_{XY} = \frac{\delta^b_{AO}}{h + L\sin\theta} \tag{S31}$$

Remarks. Effective elastic properties, E_2 and ν_{21}

Effective elastic properties for the loading in direction-Y have been quantified by the effective elastic modulus (E_2) and effective Poisson's ratio, ν_{21}. The effective elastic modulus can be obtained as

$$E_2 = \frac{\sigma_Y}{\epsilon_Y} = \frac{\lambda_1^{E_2}}{\lambda_2^{E_2} + \lambda_3^{E_2}\left(\frac{V_u^s}{\sigma_y}\right)} \tag{S32}$$

The effective Poisson's ratio, ν_{21} can be deduced by taking a negative ratio of strain perpendicular to and parallel to Y-directional loading. The net strain ϵ_X is given as

$$\epsilon_X = -\frac{-\delta_{BO}^a \cos\theta + \delta_{BO}^b \sin\theta}{L\cos\theta} \tag{S33}$$

Therefore, the effective Poisson's ratio can be obtained as

$$\nu_{21} = -\frac{\epsilon_X}{\epsilon_Y} = \frac{\beta_1^{\nu_{21}} + \beta_2^{\nu_{21}}\left(\frac{V_u^s}{\sigma_y}\right)}{\beta_3^{\nu_{21}} + \beta_4^{\nu_{21}}\left(\frac{V_u^s}{\sigma_y}\right)} \tag{S34}$$

The expressions of the coefficients ($\lambda_i^{E_1}$ and $\beta_i^{\nu_{12}}$) are given in section S7 of supplementary material where the same ratios as mentioned earlier have been used to simplify the expressions. Similar dependency trends of voltages and elastic properties (refer to equation S21 and S23) can be observed in equation S32 and S34.

S3.3. Far-field shear stress

The derivation of shear deformation and its associated normal deformations has been presented in this section by utilizing the principle of superposition. A remote shear stress τ_{XY} is applied on the lattice (and subsequently the unit cell) in a clockwise manner (refer to Figure S4). Considering the equilibrium of the unit cell ($\sum M = 0$) and writing τ_{XY} in terms of nodal forces (F and S), the following expression can be given for S

$$\sum M = 0 \Rightarrow S = \frac{F(h + L\sin\theta)}{2L\cos\theta} \tag{S35}$$

where $F = 2\tau_{XY}Lw\cos\theta$. Figure S4(A) shows the free-body diagram of the unit cell where the influence of piezoelectric effects are illustrated in terms of their equivalent moments and forces. The total shear deflection of the unit cell here will be contributed by the vertical member (AO) comprising of its bending deflection as well as rotational deflection with respect to the joint O. On the other hand, the piezoelectric voltages (V_u^s and V_l^s) on slant members will have no effect on the rotation of AO, as they are canceling each other at joint O. Rather, due to the symmetric nature of the piezoelectric loads of slant members, these voltages (V_u^s and V_l^s) will cause normal deformation to the unit cell.

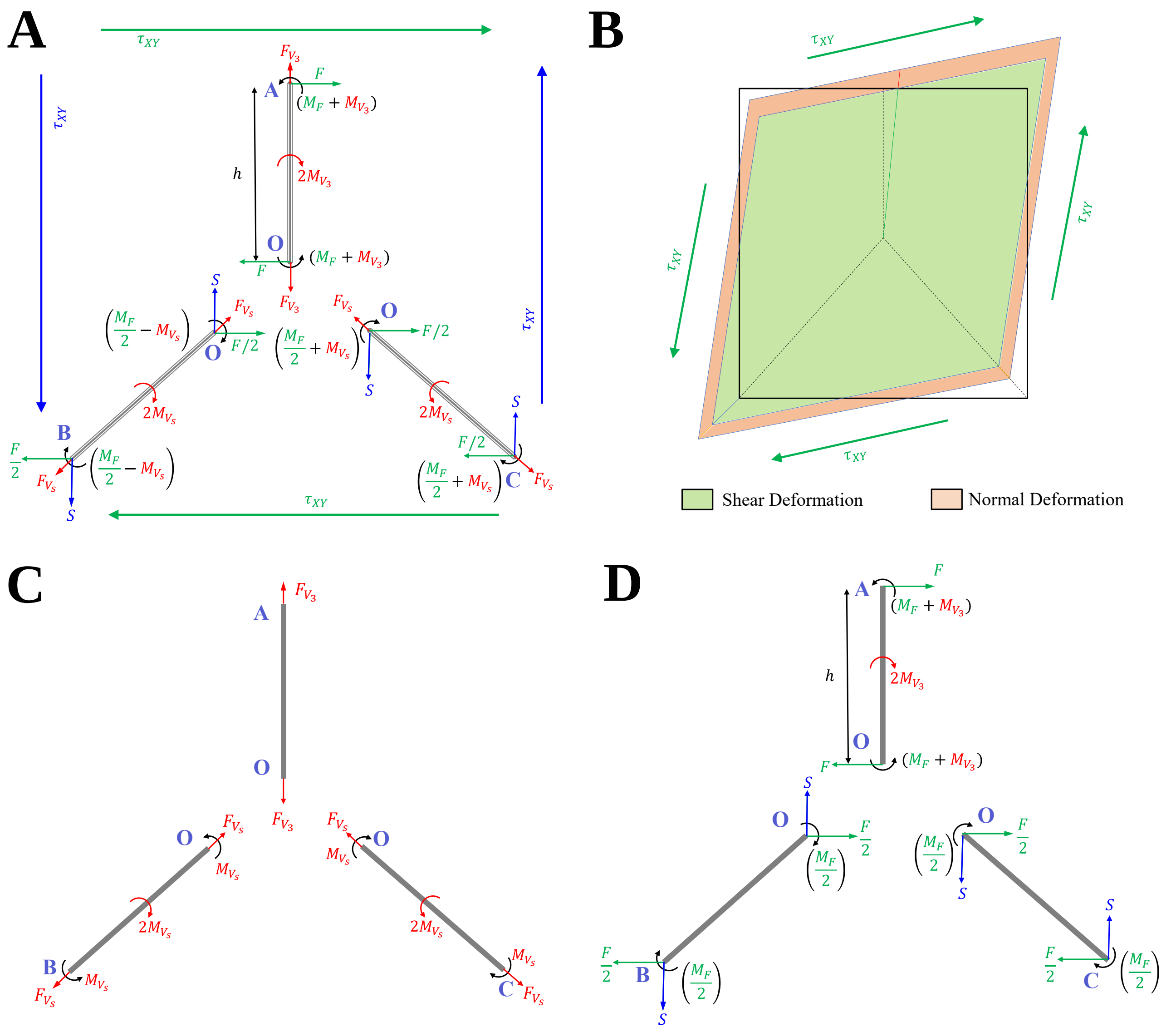

Fig. S4: Force analysis and free body diagram of the unit cell under far-field shear stresses. (A) Overall free body diagram of unit cell under in-plane positive shear loading τ_{xy}. **(B)** Schematic diagram of deformed shape of the unit cell where the green region represents deformation contributed by only mechanical shear stress, whereas the orange region is contributed by its coupled normal strains in X and Y-directions. **(C)** Force analysis of the unit cell for pure shear strain of the unit cell. **(D)** Force analysis of the unit cell for its coupled normal strains. Here contributions of piezoelectric loading are shown in terms of equivalent nodal forces and moments. The joints in the unit cell (A, B, C and O) are indicated using blue color to differentiate these from subfigure numbers.

An approximate overall deformed configuration of the unit cell is depicted schematically in Figure S4(B), where the coupled normal deformation originates from the piezoelectric voltages of the members. The expressions of these shear and normal deformations are derived separately. Figure S4(A) is analyzed by dividing it into two figures (Figure S4(C-D)), where Figure S4(C) is responsible for shear deformation whereas Figure S4(D) is for normal deformation.

Considering Figure S4(C), the bending deflection of the end A with respect to the joint O in the

direction of τ_{XY} can be given as

$$\delta^b_{AO} = \frac{h^3\, s^E_{11}\, s^m_{11}}{w s^t_e}\, F - \left(\frac{3h^2\, d_{31}\, s^m_{11}\, (t_m + t_p)}{2 s^t_e}\right) (V^3_u + V^3_l) \tag{S36}$$

The point O is acted upon by the reaction moment M_F (due to mechanical load F) and the piezoelectric applied moment M_{V_3} (though M_{V_3} will have no effect on slant members, as discussed in the preceding section). Hence, the moment M_F would be equally distributed to the slant members due to the equal stiffnesses of the members and the rotations ($\phi_{OB} = \phi_{OC} = \phi$) of two adjacent members (OB and OC) at the joint O will be the same. Here M_F can be given as

$$M_F = \frac{Fh}{2} \tag{S37}$$

Now to calculate the rotation, the relative defection of joint O with respect to the end B (or C) can be derived by changing the length-span of the beam in equation S9 from $L/2$ to L and the final expression can be given as

$$\delta_{OB} = \frac{4\, L^3\, s^E_{11}\, s^m_{11}}{w\, s^t_e} \left(\frac{-F \sin\theta}{2} + S \cos\theta\right) - \frac{6\, L^2\, s^E_{11}\, s^m_{11}}{w\, s^t_e} \left(\frac{Fh}{4}\right)$$

$$\Rightarrow \delta_{OB} = \frac{F\, L^2\, h\, s^E_{11}\, s^m_{11}}{2\, w\, s^t_e}$$

Thus the rotation (ϕ) of the joint O can be obtained from δ_{OB} as follows

$$\begin{aligned} \phi &= \frac{\delta_{OB}}{L} \\ &= \frac{F\, L\, h\, s^E_{11}\, s^m_{11}}{2\, w\, s^t_e} \end{aligned} \tag{S38}$$

Therefore the shear deformation in X-direction at the end A as per Figure S4(C) can be expressed as

$$\delta^s_{AO} = \delta^b_{AO} + h\phi \tag{S39}$$

The total axial force acting in BO direction according to Figure S4(C) is given as

$$F^a_{BO} = \frac{F}{2} \cos\theta + S \cos\theta \tag{S40}$$

The axial deflection at joint B with respect to joint O can be obtained from equation S6 as

$$\delta^a_{BO} = \frac{L\, s^m_{11}\, (s^E_{11})}{w\, (s^E_{11}\, t_m + 2\, s^m_{11}\, t_p)} \left(\frac{F}{2} \cos\theta + S \cos\theta\right) \tag{S41}$$

Subsequently, the total shear strain as per Figure S4(C) can be expressed as

$$\gamma_{XY} = \frac{\delta^s_{AO}}{(h + L \sin\theta)} + \frac{\delta^a_{BO} \cos\theta}{(h + L \sin\theta)} + 2 \frac{\delta^a_{BO} \sin\theta}{(2L \cos\theta)} \tag{S42}$$

Now considering Figure S4(D) for the coupled normal deformation of the unit cell, the deformations consist of (1) the bending (δ^b_{BO}) and axial deflections (δ^A_{BO}) of B with respect to joint O due to the

voltages V_u^s and V_l^s and (2) the axial deflection (δ_{AO}^a) of the end A with respect to O due to voltages V_u^3 and V_l^3. These deflections can be derived similar to equations S6 and S12, as follows

$$\delta_{BO}^{A} = \frac{L\, s_{11}^{m}\, (V_l^s\, d_{31}\, w - V_u^s\, d_{31}\, w)}{w\, (s_{11}^{E}\, t_m + 2\, s_{11}^{m}\, t_p)} \tag{S43}$$

$$\delta_{BO}^{b} = -\left(\frac{3L^2\, d_{31}\, s_{11}^{m}\, (t_m + t_p)}{2s_e^t}\right) (V_u^s + V_l^s) \tag{S44}$$

$$\delta_{AO}^{a} = \frac{h\, s_{11}^{m}\, (V_l^3\, d_{31}\, w - V_u^3\, d_{31}\, w)}{w\, (s_{11}^{E}\, t_m + 2\, s_{11}^{m}\, t_p)} \tag{S45}$$

Subsequently, the total coupled normal strain considering Figure S4(D) along X and Y-direction can be expressed as

$$\epsilon_X^C = \frac{\delta_{BO}^A \cos\theta + \delta_{BO}^b \sin\theta}{L\cos\theta} \tag{S46a}$$

$$\epsilon_Y^C = \frac{\delta_{AO}^a + \delta_{BO}^A \sin\theta - \delta_{BO}^b \cos\theta}{h + L\sin\theta} \tag{S46b}$$

Remarks. Effective Shear modulus, G_{12}

The effective shear modulus G_{12} can be deduced by taking the ratio of applied shear stress τ_{XY} and the derived total shear strain γ_{XY} as follows

$$G_{12} = \frac{\tau_{XY}}{\gamma_{XY}} = \frac{\lambda_1^{G_{12}}}{\lambda_2^{G_{12}} + \lambda_3^{G_{12}}\left(\frac{V_u^s}{\tau_{XY}}\right)} \tag{S47}$$

The coefficients $\lambda_i^{G_{12}}(i = 1, 2, 3)$ are given in section S8 of supplementary material. It can be noted that the shear modulus is voltage-dependent in the proposed metamaterial along with Young's moduli and Poisson's ratios.

S4. Uncoupled normal and shear modes: Notion of partial mode cloaking

Conditions for decoupled responses are extracted here for all three stress cases (two normal and one shear far-field stresses) from their final derived strain equations. We aim to achieve an unprecedented completely decoupled response, wherein only shear strain (and no normal strain) can be obtained under far-field normal stresses, and only normal strain (and no shear strain) can be obtained under far-field shear stresses.

Under the application of external mechanical uni-axial normal stress (σ_x) in X-direction, only shear strain (and no normal strain) can be obtained by equating equations 3(a) and 3(b) of the main paper to zero as follows

$$\epsilon_x = \epsilon_y = 0 \tag{S48}$$

Substituting the expressions for δ^b_{BO}, δ^a_{BO} and δ^a_{AO} into equation S48, two three-variable (i.e. V_u, V_l and σ_x) equations can be deduced. To obtain the unique combination of these three variables for getting zero normal strains, one intuitive graphical approach is considered. Two equations ($\epsilon_x = 0$, $\epsilon_y = 0$) are plotted as two separate surfaces taking voltages as independent variables. Here voltages are taken within a reasonable range i.e. -100 V to 100 V. It has been noticed that two surfaces (planes) are intersecting in nature. The values lying on the intersection edge (the red-dotted line in Figure S10) give the desired combination. Figure S10(B) shows the non-zero uncoupled shear strain values for the obtained values of voltages. It is to be noted here that to make shear strain uncoupled, it is crucial to apply the obtained normal stress values along with voltages. As it is noticed that two planes are intersecting only along a particular intersecting line, Equation S48 can also be solved parametrically to obtain closed form expressions of V_u, V_l and σ_x for which uncoupled shear strain γ^{UC}_{XY} is non-zero. The final parameterized expressions are as follows (where t is a free variable)

$$\sigma_x = t \tag{S49}$$

$$V_u = ts^E_{11}(h + L\sin\theta)\frac{(2L^2(L + h\sin\theta)\sin\theta + 3L^2(t_m + t_p)\cos\theta)s^T_e + 2hs^t_e\cos^2\theta}{6Ld_{31}(t_m + t_p)(L + h\sin\theta)s^T_e} \tag{S50}$$

$$V_l = ts^E_{11}(h + L\sin\theta)\frac{(2L^2(L + h\sin\theta)\sin\theta - 3L^2(t_m + t_p)\cos\theta)s^T_e + 2hs^t_e\cos^2\theta}{6Ld_{31}(t_m + t_p)(L + h\sin\theta)s^T_e} \tag{S51}$$

$$\gamma^{UC}_{XY} = -th^2 s^E_{11}s^m_{11}\frac{L^2 s^T_e\sin\theta(L + h\sin\theta) + hs^t_e\cos^2\theta}{L(L + h\sin\theta)s^T_e s^t_e} \tag{S52}$$

where $s^T_e = s^E_{11}t_m + 2s^m_{11}t_p$. In the above set of equations, for a given value t of the applied far-field stress σ_x, the relationship with voltages can be obtained for uncoupled normal and shear modes. Note that under the conditions stated in Equation S49 - S51 (under far-field X-direction stress), $\epsilon_x = \epsilon_y = 0$, while the non-zero γ^{UC}_{XY} is given by Equation S52.

Similar operations are performed for the case of Y-directional normal far-field stress σ_y (refer to Figure S10(C-D)). The final parametric expressions involving V_u, V_l, σ_y and the corresponding uncoupled shear strain γ^{UC}_{XY} are as follows

$$\sigma_y = t \tag{S53}$$

$$V_u = ts^E_{11}\cos\theta\frac{h(s^t_e - L^2 s^T_e)\sin 2\theta - 2s^T_e(L^3 + 2h)\cos\theta + 3Ls^T_e(L + 2h)(t_m + t_p)}{6d_{31}s^T_e(t_m + t_p)(L + h\sin\theta)} \tag{S54}$$

$$V_l = -ts^E_{11}\cos\theta\frac{2s^T_e(L^3 + 2h)\cos\theta - h(s^t_e - L^2 s^T_e)\sin 2\theta + 3Ls^T_e(L + 2h)(t_m + t_p)}{6d_{31}s^T_e(t_m + t_p)(L + h\sin\theta)} \tag{S55}$$

$$\gamma^{UC}_{XY} = t\frac{h^2 s^E_{11}s^m_{11}\cos^2\theta}{s^T_e s^t_e}\frac{(s^T_e L^3 + 2hs^t_e) - h\sin\theta(s^T_e L^2 - s^t_e)}{(L^2 + h^2)\sin\theta + Lh\sin^2\theta} \tag{S56}$$

In the above set of equations, for a given value t of the applied far-field stress σ_y, the relationship with voltages can be obtained for uncoupled normal and shear modes. Note that under the conditions stated

in Equation S53 - S55 (under far-field Y-direction stress), $\epsilon_x = \epsilon_y = 0$, while the non-zero γ_{XY}^{UC} is given by Equation S56.

In the case of applied far-field shear stress, as the objective is to obtain uncoupled normal strain, the derived shear strain expression i.e. Equation 9(c) is equated to zero as follows

$$\gamma_{xy} = 0 \tag{S57}$$

After substituting the expressions δ_{AO}^a and δ_{BO}^a into equation S57, the expression involving V_u, V_l and τ_{xy} is obtained which is plotted in Figure S10(E). The values onto the surface gives the uncoupled state of shear stress. Figure S10(F) shows the non-zero values of two uncoupled normal strains under the application of previously obtained values of V_u, V_l and τ_{xy}. Coming to parametric solution, final closed form expressions of V_u, V_l, τ_{xy} for which two uncoupled normal strains (ϵ_X^{UC} and ϵ_X^{UC}) can be obtained are as follows (t_1, t_2 are defined as two free variables)

$$(V_u, V_l) = (t_1, t_2) \tag{S58}$$

$$\tau_{xy} = \frac{3Ld_{31}h^2 s_e^T w \cos\theta (t_m + t_p)(t_2 + t_1)}{\left(\begin{array}{c} s_{11}^E s_e^T L h^2 \cos\theta (L + 2h) + s_{11}^E s_e^t L^2 (\cos^3\theta + \sin^3\theta) + s_{11}^E s_e^t L h (1 + \sin^2\theta) \\ + s_{11}^E s_e^t \cos\theta \sin\theta (\cos\theta + \sin\theta) + s_{11}^E s_e^t L h \cos\theta \sin\theta + s_{11}^E s_e^t h^2 \sin\theta \end{array} \right)} \tag{S59}$$

$$\epsilon_X^{UC} = -\frac{3Ld_{31}s_{11}^m s_e^T \sin\theta (t_m + t_p)(t_1 + t_2) + 2d_{31}s_{11}^m s_e^t \cos\theta (t_1 - t_2)}{2s_e^T s_e^t \cos\theta} \tag{S60}$$

$$\epsilon_Y^{UC} = \frac{3L^2 d_{31}s_{11}^m s_e^T \cos\theta (t_m + t_p)(t_1 + t_2) + 2d_{31}s_{11}^m s_e^t (h + L\sin\theta)(t_2 - t_1)}{2s_e^T s_e^t (h + L\sin\theta)} \tag{S61}$$

In the above set of equations, for a given values of t_1 and t_2 of the applied voltages, the relationship with shear stress can be obtained for uncoupled normal and shear modes. Note that under the conditions stated in Equation S58 (under far-field shear stress), $\gamma_{xy} = 0$, while non-zero ϵ_X^{UC} and ϵ_Y^{UC} are given by Equation S59 - S61.

Note that in the above three scenarios, even though we have concentrated on the condition of zero strains in the direction and mode of applied far-field stress, other specific targeted values of strains can also be readily obtained depending on application specific requirements.

S5. Additional numerical results

Table S1: Material and structural parameters of the honeycomb lattice for validation study

Parameter	Unit	Value
Elastic Compliance of Piezo layer, s_{11}^{E}	1/Pa	1.65×10^{-11}
Elastic Compliance of substrate layer, s_{11}^{m}	1/Pa	1.42×10^{-11}
Piezoelectric coupling coefficient, d_{31}	C/N	-2.74×10^{-10}
Beam (Cell wall) length, $L(=h)$	mm	60
Piezo layer thickness, t_p	mm	0.1
Substrate layer thickness, t_m	mm	0.3
Out-of-plane width, w	mm	3
Cell angle, θ	deg	30

Table S2: Critical hybrid-voltage ratios for elastic constants

Elastic Constants	t_R	Critical hybrid-voltage-ratio (V_R^0)
E_1	1	-3.302
	5	-4.96
E_2	1	-2.71
	5	-3.96
G_{12}	1	-9.34
	5	-14.904
ν_{12}	1	-3.293
	5	-4.954
ν_{21}	1	0.80
	5	2.02

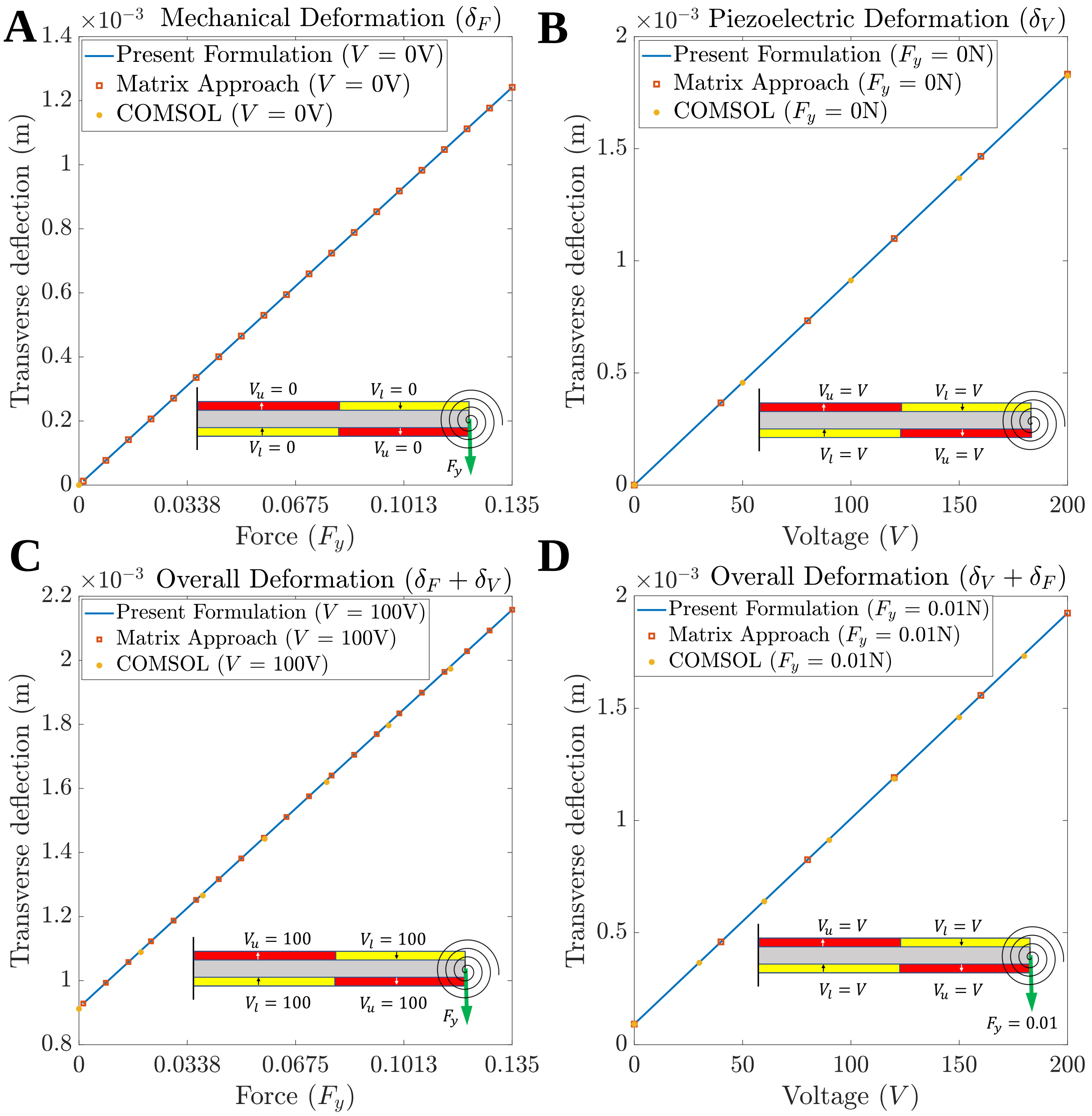

Fig. S5: Beam-level validation under transverse load. Numerical validation of the current analytic beam model is presented with respect to the finite element based approach (COMSOL) and assembled direct stiffness method (matrix approach) under one end fixed and the other rotationally restrained boundary condition. **(A)** Variation of transverse deformation with mechanical load under the mechanical pure-bending state of the beam. **(B)** Variation of transverse deformation with piezoelectric load under piezoelectric pure-bending state of the beam. **(C-D)** Variation of total transverse deformation under simultaneous mechanical and piezoelectric loading. Note that here the results are shown within a force range with a minimum transverse force of 1.35 mN and a maximum force of 0.135 N at the beam (cell-wall) level and this range is determined based on the values of geometric parameters (listed in Table S1) and the actual far-field stress range (10-1000 N m^{-2}) and voltage range (up to 200 V) considered in the present paper (refer to the Normal-shear mode coupling section) for entire honeycomb lattice. A maximum tip-deformation of 2% of the beam length is obtained which is reasonable to assume under the small deformation assumption. The spiral here symbolizes an infinitely stiff torsional spring, denoting the rotationally restrained boundary condition at the beam end. The force is given here in N unit.

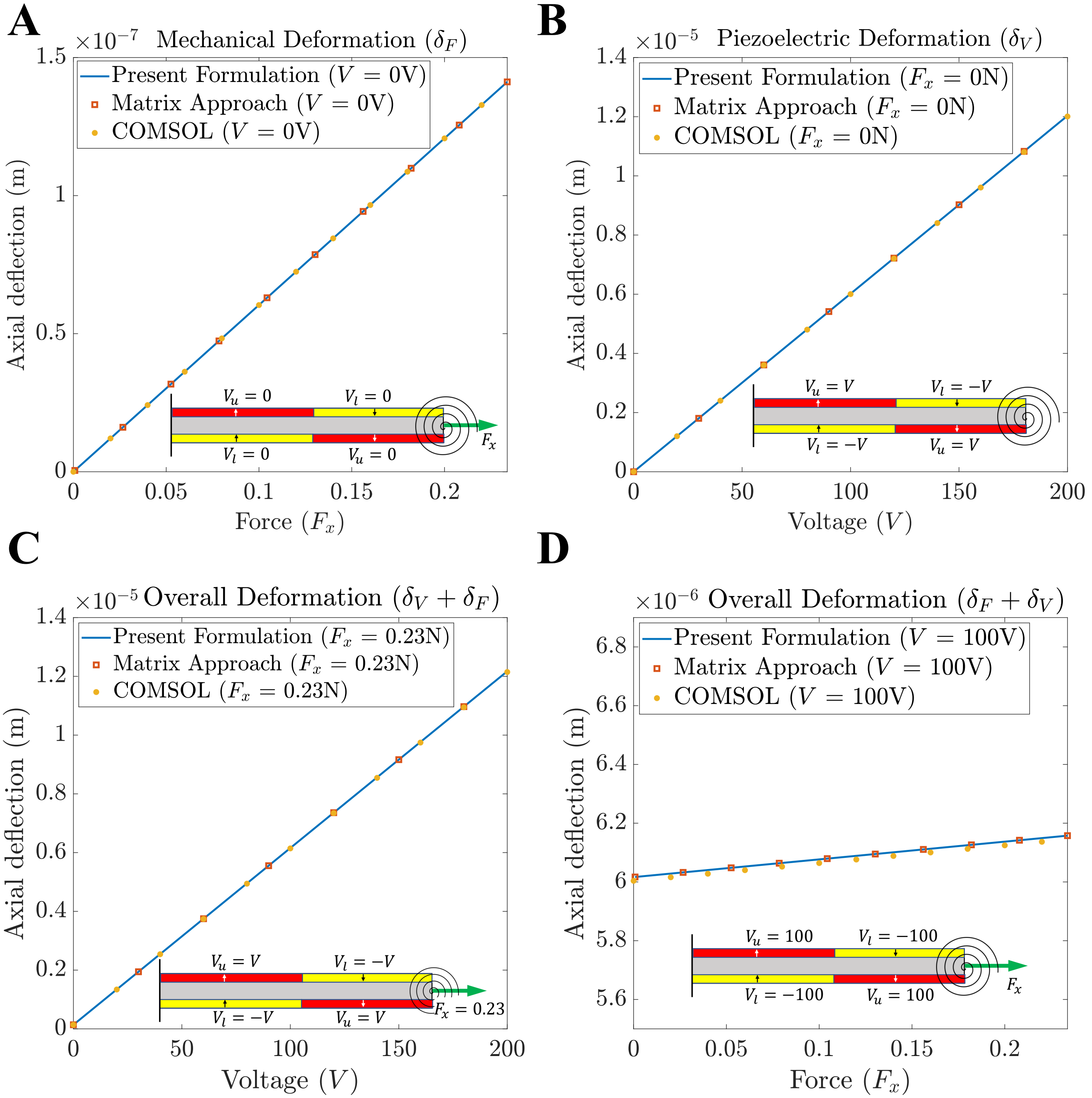

Fig. S6: Beam-level validation under axial load. Numerical validation of the current analytic beam model is presented with respect to the finite element based approach (COMSOL) and assembled direct stiffness method (matrix approach) considering one end fixed and the other rotationally restrained boundary condition. **(A)** Variation of axial deformation with mechanical load (axial). **(B)** Variation of axial deformation with piezoelectric load under piezoelectric pure-axial state of the beam. **(C-D)** Variation of total axial deformation under both mechanical and piezoelectric loading. Note that here the results are shown within a force range with a minimum axial force of 0.78 mN and a maximum force of 0.234 N at the beam (cell-wall) level and this range is determined based on the values of geometric parameters listed in Table S1 along with the actual actual far-field stress range (10-1000 N m^{-2}) and voltage range (up to 200 V) (refer to the Normal-shear mode coupling section) for entire honeycomb lattice. The spiral here symbolizes an infinitely stiff torsional spring, denoting the rotationally restrained boundary condition at the beam end. The force is given here in N unit.

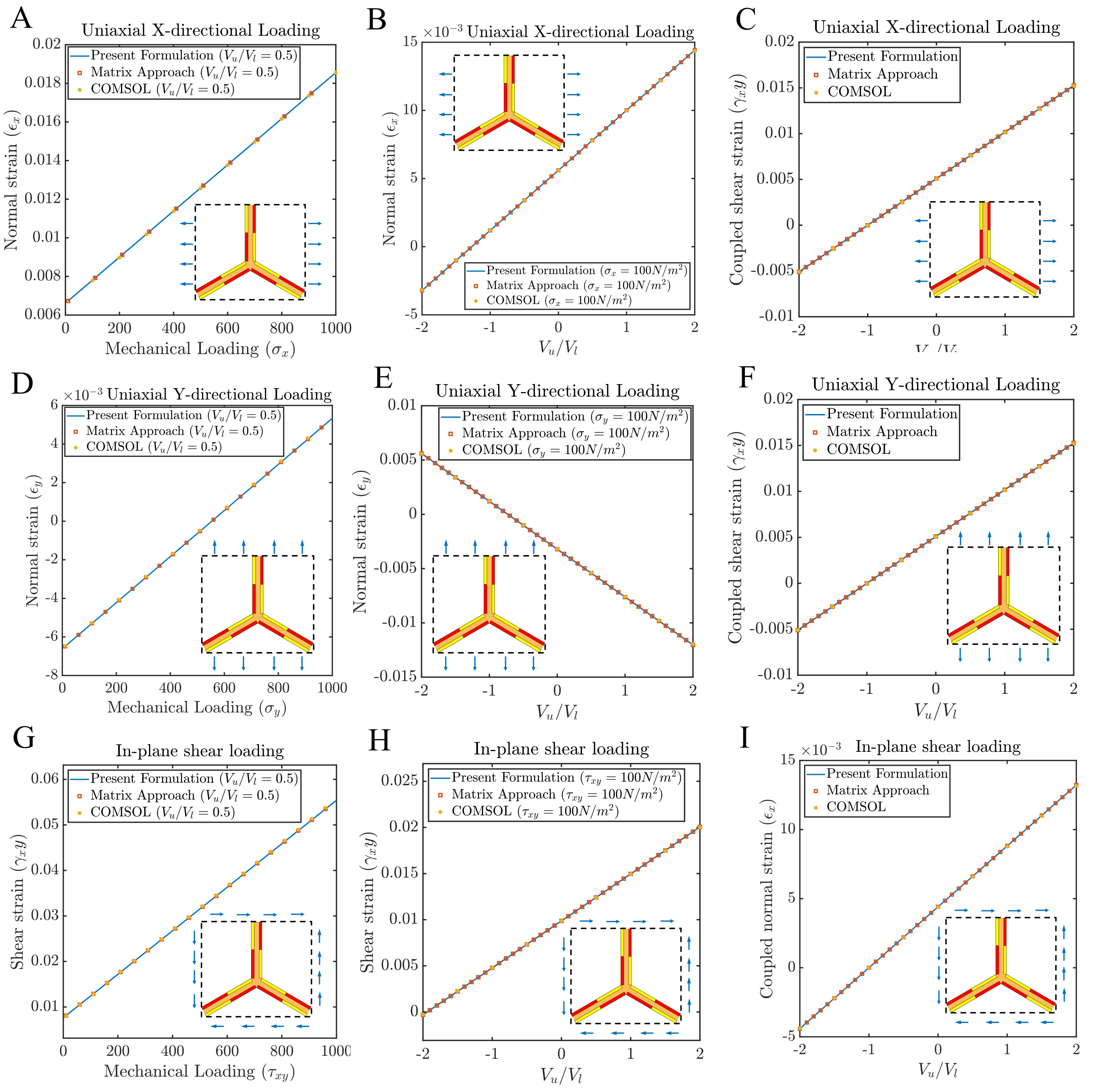

Fig. S7: Unit cell and lattice-level validation of the proposed bottom-up beam-based approach. The present formulation for analyzing the active metamaterial is validated with unit cell based assembled direct stiffness method (matrix approach) and finite element-based approach considering piezoelectric elements. **(A-C)**Variation of X-directional direct strain and its associated shear strain with mechanical uni-axial stress σ_x and piezoelectric hybrid-voltage ratio. **(D-F)** Variation of Y-directional direct strain and its coupled shear strain with mechanical uni-axial stress σ_y and piezoelectric hybrid-voltage ratio. **(G-I)** Variation of in-plane shear strain and its coupled x-directional normal strain with applied stress τ_{xy} and piezoelectric hybrid-voltage ratio. Here the term piezoelectric hybrid-voltage ratio refers to the ratio V_u/V_s i.e. the ratio of applied voltages on the upper and bottom surfaces of the piezoelectric layer. The mechanical loadings are given in N m^{-2} units.

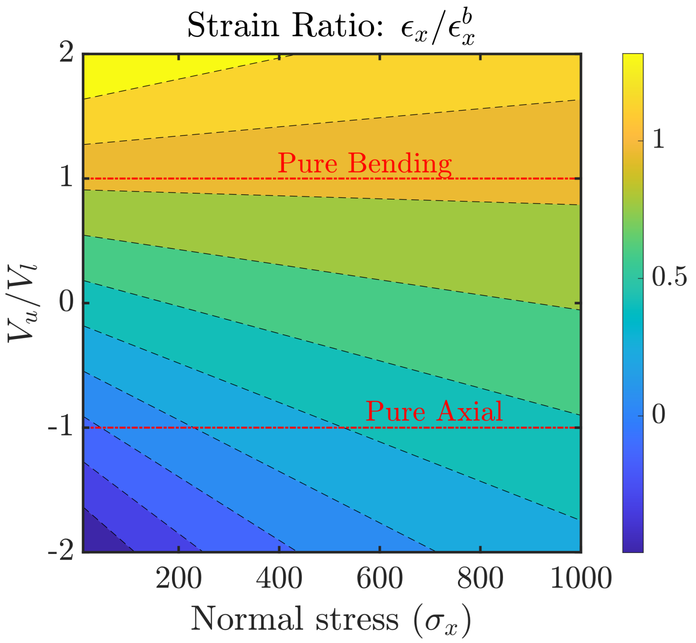

Fig. S8: Contour plot of normalized normal strain in X-directional loading with respect to the normal strain at pure piezoelectric bending state ($V_u = V_l$). This plot highlights the effect of having a difference in V_u and V_l. Similar results can be obtained for other modes of far-field stress and the resulting strain components. The red-dotted lines highlight the two different piezoelectric actuation modes i.e. pure-bending ($V_u = V_l$) and pure-axial ($V_u = -V_l$). The normal stress is given in N m^{-2} units.

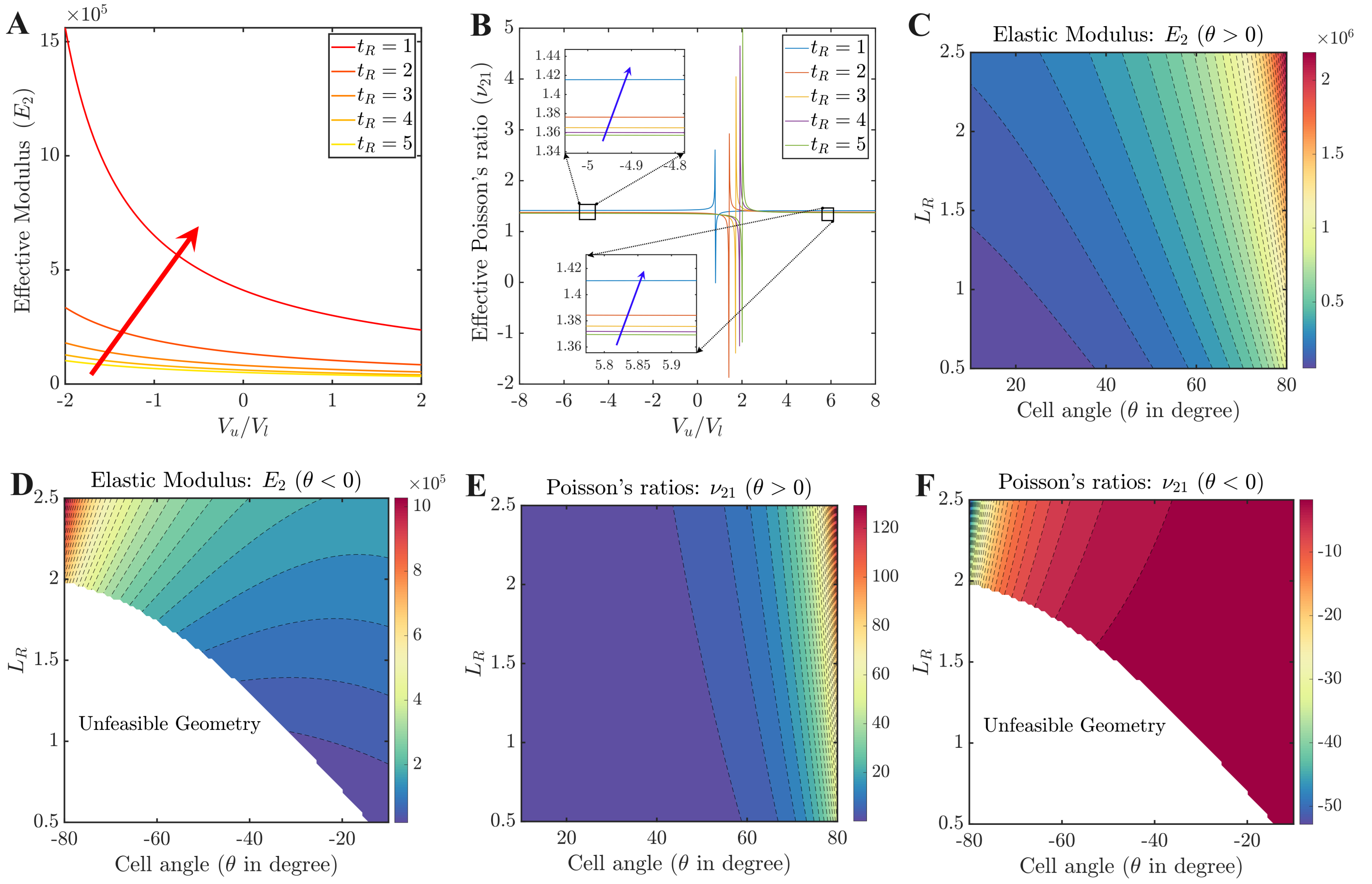

Fig. S9: Influence of lattice architecture and external stimuli on the effective elastic properties. (A) Variation of Young's modulus, E_2 with hybrid-voltage ratio at constant mechanical normal stress (σ_y) for different values of t_R. **(B)** Variation of Poisson's ratio, ν_{21} with hybrid-voltage ratio at constant normal stress (σ_y) for different values of t_R. **(C, D)** Coutour plots of elastic modulus, E_2 for non-auxetic and auxetic lattices as a function of $L_R(= h/L)$ and cell angle (θ). **(E, F)** Coutour plots of effective Poisson's ratio, ν_{21} for non-auxetic and auxetic lattices as a function of $L_R(= h/L)$ and cell angle (θ). Here the constant stresses ($\sigma_x, \sigma_y, \tau_{xy}$) and the voltage, V_l are kept equal to 1000Pa and 100V, whereas the length of inclined member and thickness of the substrate layer are kept at 60 mm and 0.3 mm, respectively. The arrows (red in sub-figure A, blue in sub-figure B) denote an increase of piezo thickness. In first two sub-figures, the following condition is conformed $L_R = 1.5$ and $\theta = 30$, while in the remaining sub-figures we consider $t_R = 1$ and $V_R = 3$. The elastic moduli are here given in N m^{-2}.

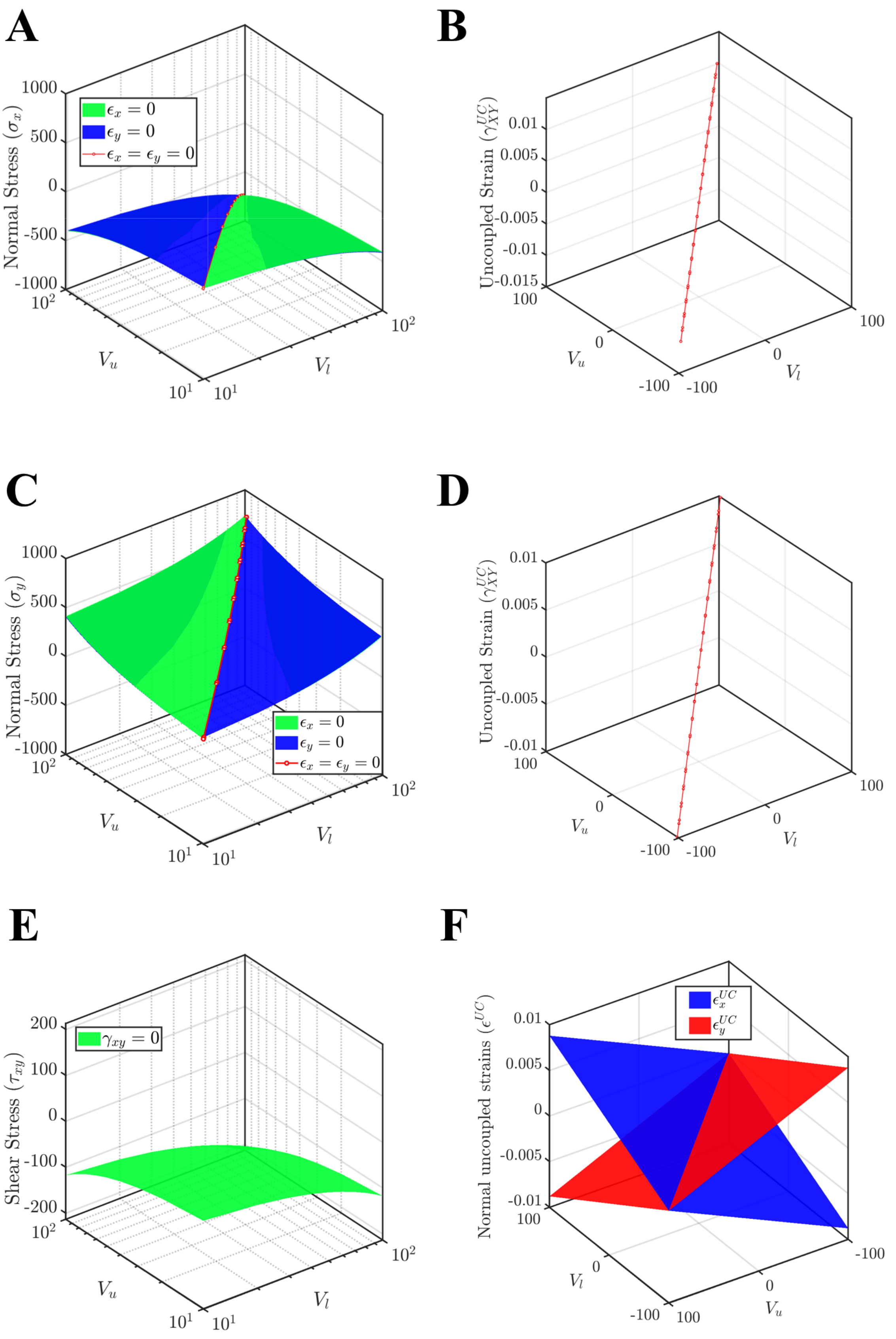

Fig. S10: **(A)** Solution for zero normal strains under X-directional normal far-field stress. **(B)** Uncoupled shear strain under normal stress, σ_x (for the condition of no normal strains). **(C)** Solution for zero normal strains under Y-directional normal far-field stress. **(D)** Uncoupled shear strain under normal stress, σ_y (for the condition of no normal strains). **(E)** Solution for zero shear strain under far-field shear stress. **(F)** Uncoupled normal strains under shear stress, τ_{xy} (for the condition of no shear strain). The mechanical stresses are here given in N m^{-2}, while voltages are given in V units.

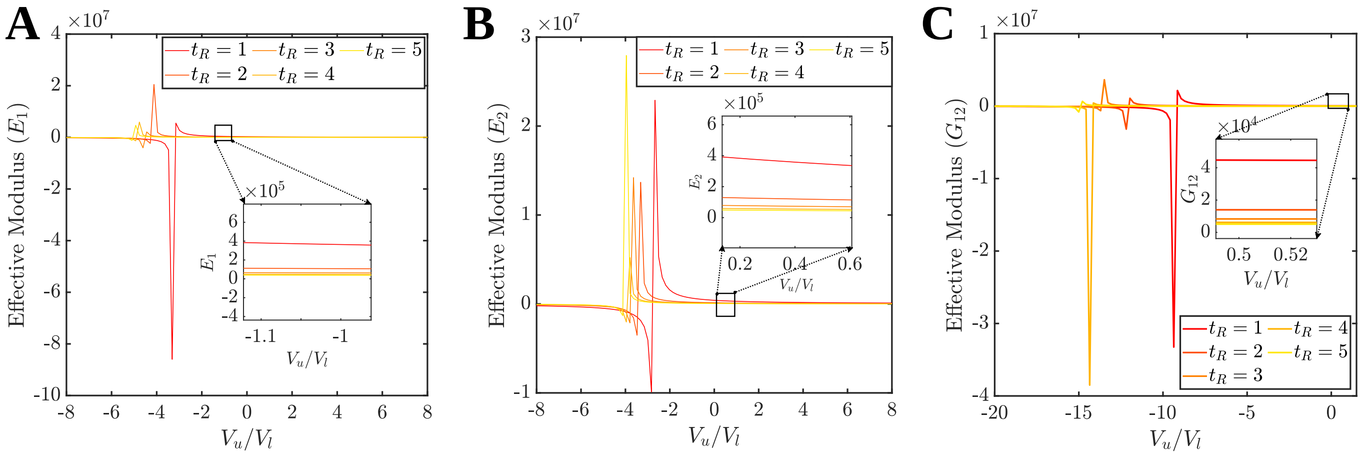

Fig. S11: Existence of a single critical voltage ratios for each piezo-thickness-ratio. The variation is plotted for the following elastic modulli: **(A)** E_1, **(B)** E_2, **(C)** G_{12}. Visual evidence of critical voltages beyond the voltage ratio range mentioned in Figure 5(A-B) and S9(A) is according to the Table S2. The elastic moduli are here given in N m^{-2}.

S6. Coefficients of E_1 and ν_{12}

The expressions are obtained in symbolic environment where the following constants are named as follows: $V_{3,R} = V_R^3$, $Vs_R = V_R^s$, $s_{11R} = s_{11}^R$, $s_{11E} = s_{11}^E$.

$$\lambda_1^{E_1} = 2\,\mathrm{Vs}_R\, t_p\, \cos(\theta)\, \left(2\, s_{11\mathrm{R}} + t_R\right) \left({t_R}^3\, {t_p}^2 + 6\, s_{11\mathrm{R}}\, {t_R}^2\, {t_p}^2 + 12\, s_{11\mathrm{R}}\, t_R\, {t_p}^2 + 8\, s_{11\mathrm{R}}\, {t_p}^2\right)$$

$$\begin{aligned}\lambda_2^{E_1} = {} & 4\,\mathrm{Vs}_R\, s_{11\mathrm{E}}\, L^3\, {s_{11\mathrm{R}}}^2 \sin(\theta)^3 + 4\, L_R\, \mathrm{Vs}_R\, s_{11\mathrm{E}}\, L^3\, {s_{11\mathrm{R}}}^2 \sin(\theta)^2 + 2\,\mathrm{Vs}_R\, s_{11\mathrm{E}}\, L^3\, s_{11\mathrm{R}}\, t_R \sin(\theta)^3 \\
& + 2\, L_R\, \mathrm{Vs}_R\, s_{11\mathrm{E}}\, L^3\, s_{11\mathrm{R}}\, t_R \sin(\theta)^2 + 12\,\mathrm{Vs}_R\, s_{11\mathrm{E}}\, L\, {s_{11\mathrm{R}}}^2\, {t_R}^2\, {t_p}^2 \cos(\theta)^2\, \sin(\theta) \\
& + 12\, L_R\, \mathrm{Vs}_R\, s_{11\mathrm{E}}\, L\, {s_{11\mathrm{R}}}^2\, {t_R}^2\, {t_p}^2 \cos(\theta)^2 + 24\,\mathrm{Vs}_R\, s_{11\mathrm{E}}\, L\, {s_{11\mathrm{R}}}^2\, t_R\, {t_p}^2 \cos(\theta)^2\, \sin(\theta) \\
& + 24\, L_R\, \mathrm{Vs}_R\, s_{11\mathrm{E}}\, L\, {s_{11\mathrm{R}}}^2\, t_R\, {t_p}^2 \cos(\theta)^2 + 16\,\mathrm{Vs}_R\, s_{11\mathrm{E}}\, L\, {s_{11\mathrm{R}}}^2\, {t_p}^2 \cos(\theta)^2\, \sin(\theta) \\
& + 16\, L_R\, \mathrm{Vs}_R\, s_{11\mathrm{E}}\, L\, {s_{11\mathrm{R}}}^2\, {t_p}^2 \cos(\theta)^2 + 2\,\mathrm{Vs}_R\, s_{11\mathrm{E}}\, L\, s_{11\mathrm{R}}\, {t_R}^3\, {t_p}^2 \cos(\theta)^2\, \sin(\theta) \\
& + 2\, L_R\, \mathrm{Vs}_R\, s_{11\mathrm{E}}\, L\, s_{11\mathrm{R}}\, {t_R}^3\, {t_p}^2 \cos(\theta)^2\end{aligned}$$

$$\begin{aligned}\lambda_3^{E_1} = {} & 16\, d_{31}\, {s_{11\mathrm{R}}}^2\, {t_p}^2\, \cos(\theta) + 12\, d_{31}\, {s_{11\mathrm{R}}}^2\, {t_R}^2\, {t_p}^2\, \cos(\theta) - 6\, L\, d_{31}\, {s_{11\mathrm{R}}}^2\, t_p\, \sin(\theta) - 16\,\mathrm{Vs}_R\, d_{31}\, {s_{11\mathrm{R}}}^2\, {t_p}^2\, \cos(\theta) \\
& + 24\, d_{31}\, {s_{11\mathrm{R}}}^2\, t_R\, {t_p}^2\, \cos(\theta) + 2\, d_{31}\, s_{11\mathrm{R}}\, {t_R}^3\, {t_p}^2\, \cos(\theta) - 24\,\mathrm{Vs}_R\, d_{31}\, {s_{11\mathrm{R}}}^2\, t_R\, {t_p}^2\, \cos(\theta) \\
& - 2\,\mathrm{Vs}_R\, d_{31}\, s_{11\mathrm{R}}\, {t_R}^3\, {t_p}^2\, \cos(\theta) - 3\, L\, d_{31}\, s_{11\mathrm{R}}\, t_R\, t_p\, \sin(\theta) - 12\,\mathrm{Vs}_R\, d_{31}\, {s_{11\mathrm{R}}}^2\, {t_R}^2\, {t_p}^2\, \cos(\theta) \\
& - 6\, L\, \mathrm{Vs}_R\, d_{31}\, {s_{11\mathrm{R}}}^2\, t_p\, \sin(\theta) - 3\, L\, d_{31}\, s_{11\mathrm{R}}\, {t_R}^2\, t_p\, \sin(\theta) - 6\, L\, d_{31}\, {s_{11\mathrm{R}}}^2\, t_R\, t_p\, \sin(\theta) \\
& - 3\, L\, \mathrm{Vs}_R\, d_{31}\, s_{11\mathrm{R}}\, {t_R}^2\, t_p\, \sin(\theta) - 6\, L\, \mathrm{Vs}_R\, d_{31}\, {s_{11\mathrm{R}}}^2\, t_R\, t_p\, \sin(\theta) - 3\, L\, \mathrm{Vs}_R\, d_{31}\, s_{11\mathrm{R}}\, t_R\, t_p\, \sin(\theta)\end{aligned}$$

$$\begin{aligned}\beta_1^{\nu_{12}} = {} & -\cos(\theta)\, \big(-2\, V_{3,R}\, V_R\, \mathrm{Vs}_R\, s_{11\mathrm{E}}\, \cos(\theta)\, L^3\, t_R \sin(\theta)^2 - 2\, L_R\, V_{3,R}\, V_R\, \mathrm{Vs}_R\, s_{11\mathrm{E}}\, \cos(\theta)\, L^3\, t_R\, \sin(\theta) \\
& - 4\, V_{3,R}\, V_R\, \mathrm{Vs}_R\, s_{11\mathrm{E}}\, s_{11\mathrm{R}}\, \cos(\theta)\, L^3 \sin(\theta)^2 - 4\, L_R\, V_{3,R}\, V_R\, \mathrm{Vs}_R\, s_{11\mathrm{E}}\, s_{11\mathrm{R}}\, \cos(\theta)\, L^3\, \sin(\theta) \\
& + 2\, V_{3,R}\, V_R\, \mathrm{Vs}_R\, s_{11\mathrm{E}}\, \cos(\theta)\, L\, {t_R}^3\, {t_p}^2 \sin(\theta)^2 + 2\, L_R\, V_{3,R}\, V_R\, \mathrm{Vs}_R\, s_{11\mathrm{E}}\, \cos(\theta)\, L\, {t_R}^3\, {t_p}^2\, \sin(\theta) \\
& + 12\, V_{3,R}\, V_R\, \mathrm{Vs}_R\, s_{11\mathrm{E}}\, s_{11\mathrm{R}}\, \cos(\theta)\, L\, {t_R}^2\, {t_p}^2 \sin(\theta)^2 + 12\, L_R\, V_{3,R}\, V_R\, \mathrm{Vs}_R\, s_{11\mathrm{E}}\, s_{11\mathrm{R}}\, \cos(\theta)\, L\, {t_R}^2\, {t_p}^2\, \sin(\theta) \\
& + 24\, V_{3,R}\, V_R\, \mathrm{Vs}_R\, s_{11\mathrm{E}}\, s_{11\mathrm{R}}\, \cos(\theta)\, L\, t_R\, {t_p}^2 \sin(\theta)^2 + 24\, L_R\, V_{3,R}\, V_R\, \mathrm{Vs}_R\, s_{11\mathrm{E}}\, s_{11\mathrm{R}}\, \cos(\theta)\, L\, t_R\, {t_p}^2\, \sin(\theta) \\
& + 16\, V_{3,R}\, V_R\, \mathrm{Vs}_R\, s_{11\mathrm{E}}\, s_{11\mathrm{R}}\, \cos(\theta)\, L\, {t_p}^2 \sin(\theta)^2 + 16\, L_R\, V_{3,R}\, V_R\, \mathrm{Vs}_R\, s_{11\mathrm{E}}\, s_{11\mathrm{R}}\, \cos(\theta)\, L\, {t_p}^2\, \sin(\theta)\big)\end{aligned}$$

$$
\begin{aligned}
\beta_2^{\nu_{12}} = & -\cos(\theta)\ (2\,L_R\,\mathrm{Vs}_R\,d_{31}\,{t_R}^3\,{t_p}^2 + 16\,L_R\,\mathrm{Vs}_R\,d_{31}\,s_{11\mathrm{R}}\,{t_p}^2 - 16\,L_R\,V_{3,R}\,\mathrm{Vs}_R\,d_{31}\,s_{11\mathrm{R}}\,{t_p}^2 \\
& + 24\,L_R\,\mathrm{Vs}_R\,d_{31}\,s_{11\mathrm{R}}\,t_R\,{t_p}^2 - 2\,L_R\,V_{3,R}\,\mathrm{Vs}_R\,d_{31}\,{t_R}^3\,{t_p}^2 + 12\,L_R\,\mathrm{Vs}_R\,d_{31}\,s_{11\mathrm{R}}\,{t_R}^2\,{t_p}^2 \\
& + 16\,V_{3,R}\,V_R\,d_{31}\,s_{11\mathrm{R}}\,{t_p}^2\,\sin(\theta) + 2\,V_{3,R}\,V_R\,d_{31}\,{t_R}^3\,{t_p}^2\,\sin(\theta) + 24\,V_{3,R}\,V_R\,d_{31}\,s_{11\mathrm{R}}\,t_R\,{t_p}^2\,\sin(\theta) \\
& - 2\,V_{3,R}\,V_R\,\mathrm{Vs}_R\,d_{31}\,{t_R}^3\,{t_p}^2\,\sin(\theta) + 12\,V_{3,R}\,V_R\,d_{31}\,s_{11\mathrm{R}}\,{t_R}^2\,{t_p}^2\,\sin(\theta) - 24\,L_R\,V_{3,R}\,\mathrm{Vs}_R\,d_{31}\,s_{11\mathrm{R}}\,t_R\,{t_p}^2 \\
& + 6\,L\,V_{3,R}\,V_R\,d_{31}\,s_{11\mathrm{R}}\,t_p\,\cos(\theta) + 3\,L\,V_{3,R}\,V_R\,d_{31}\,t_R\,t_p\,\cos(\theta) - 12\,L_R\,V_{3,R}\,\mathrm{Vs}_R\,d_{31}\,s_{11\mathrm{R}}\,{t_R}^2\,{t_p}^2 \\
& + 3\,L\,V_{3,R}\,V_R\,d_{31}\,{t_R}^2\,t_p\,\cos(\theta) - 16\,V_{3,R}\,V_R\,\mathrm{Vs}_R\,d_{31}\,s_{11\mathrm{R}}\,{t_p}^2\,\sin(\theta) + 3\,L\,V_{3,R}\,V_R\,\mathrm{Vs}_R\,d_{31}\,{t_R}^2\,t_p\,\cos(\theta) \\
& - 24\,V_{3,R}\,V_R\,\mathrm{Vs}_R\,d_{31}\,s_{11\mathrm{R}}\,t_R\,{t_p}^2\,\sin(\theta) - 12\,V_{3,R}\,V_R\,\mathrm{Vs}_R\,d_{31}\,s_{11\mathrm{R}}\,{t_R}^2\,{t_p}^2\,\sin(\theta) \\
& + 6\,L\,V_{3,R}\,V_R\,\mathrm{Vs}_R\,d_{31}\,s_{11\mathrm{R}}\,t_p\,\cos(\theta) + 3\,L\,V_{3,R}\,V_R\,\mathrm{Vs}_R\,d_{31}\,t_R\,t_p\,\cos(\theta) + 6\,L\,V_{3,R}\,V_R\,d_{31}\,s_{11\mathrm{R}}\,t_R\,t_p\,\cos(\theta) \\
& + 6\,L\,V_{3,R}\,V_R\,\mathrm{Vs}_R\,d_{31}\,s_{11\mathrm{R}}\,t_R\,t_p\,\cos(\theta))
\end{aligned}
$$

$$
\begin{aligned}
\beta_3^{\nu_{12}} = & V_{3,R}\,V_R\ (L_R + \sin(\theta))\ (2\,\mathrm{Vs}_R\,s_{11\mathrm{E}}\,L^3\,t_R\sin(\theta)^3 + 2\,L_R\,\mathrm{Vs}_R\,s_{11\mathrm{E}}\,L^3\,t_R\sin(\theta)^2 + 4\,\mathrm{Vs}_R\,s_{11\mathrm{E}}\,s_{11\mathrm{R}}\,L^3\sin(\theta)^3 \\
& + 4\,L_R\,\mathrm{Vs}_R\,s_{11\mathrm{E}}\,s_{11\mathrm{R}}\,L^3\sin(\theta)^2 + 2\,\mathrm{Vs}_R\,s_{11\mathrm{E}}\,L\,{t_R}^3\,{t_p}^2\cos(\theta)^2\,\sin(\theta) + 2\,L_R\,\mathrm{Vs}_R\,s_{11\mathrm{E}}\,L\,{t_R}^3\,{t_p}^2\cos(\theta)^2 \\
& + 12\,\mathrm{Vs}_R\,s_{11\mathrm{E}}\,s_{11\mathrm{R}}\,L\,{t_R}^2\,{t_p}^2\cos(\theta)^2\,\sin(\theta) + 12\,L_R\,\mathrm{Vs}_R\,s_{11\mathrm{E}}\,s_{11\mathrm{R}}\,L\,{t_R}^2\,{t_p}^2\cos(\theta)^2 \\
& + 24\,\mathrm{Vs}_R\,s_{11\mathrm{E}}\,s_{11\mathrm{R}}\,L\,t_R\,{t_p}^2\cos(\theta)^2\,\sin(\theta) + 24\,L_R\,\mathrm{Vs}_R\,s_{11\mathrm{E}}\,s_{11\mathrm{R}}\,L\,t_R\,{t_p}^2\cos(\theta)^2 \\
& + 16\,\mathrm{Vs}_R\,s_{11\mathrm{E}}\,s_{11\mathrm{R}}\,L\,{t_p}^2\cos(\theta)^2\,\sin(\theta) + 16\,L_R\,\mathrm{Vs}_R\,s_{11\mathrm{E}}\,s_{11\mathrm{R}}\,L\,{t_p}^2\cos(\theta)^2)
\end{aligned}
$$

$$
\begin{aligned}
\beta_4^{\nu_{12}} = & -V_{3,R}\,V_R\ (L_R + \sin(\theta))\ (16\,\mathrm{Vs}_R\,d_{31}\,s_{11\mathrm{R}}\,{t_p}^2\,\cos(\theta) - 16\,d_{31}\,s_{11\mathrm{R}}\,{t_p}^2\,\cos(\theta) - 2\,d_{31}\,{t_R}^3\,{t_p}^2\,\cos(\theta) \\
& + 3\,L\,d_{31}\,{t_R}^2\,t_p\,\sin(\theta) - 24\,d_{31}\,s_{11\mathrm{R}}\,t_R\,{t_p}^2\,\cos(\theta) + 2\,\mathrm{Vs}_R\,d_{31}\,{t_R}^3\,{t_p}^2\,\cos(\theta) - 12\,d_{31}\,s_{11\mathrm{R}}\,{t_R}^2\,{t_p}^2\,\cos(\theta) \\
& + 6\,L\,d_{31}\,s_{11\mathrm{R}}\,t_p\,\sin(\theta) + 3\,L\,d_{31}\,t_R\,t_p\,\sin(\theta) + 12\,\mathrm{Vs}_R\,d_{31}\,s_{11\mathrm{R}}\,{t_R}^2\,{t_p}^2\,\cos(\theta) + 6\,L\,\mathrm{Vs}_R\,d_{31}\,s_{11\mathrm{R}}\,t_p\,\sin(\theta) \\
& + 3\,L\,\mathrm{Vs}_R\,d_{31}\,t_R\,t_p\,\sin(\theta) + 6\,L\,d_{31}\,s_{11\mathrm{R}}\,t_R\,t_p\,\sin(\theta) + 3\,L\,\mathrm{Vs}_R\,d_{31}\,{t_R}^2\,t_p\,\sin(\theta) + 24\,\mathrm{Vs}_R\,d_{31}\,s_{11\mathrm{R}}\,t_R\,{t_p}^2\,\cos(\theta) \\
& + 6\,L\,\mathrm{Vs}_R\,d_{31}\,s_{11\mathrm{R}}\,t_R\,t_p\,\sin(\theta))
\end{aligned}
$$

S7. Coefficients of E_2 and ν_{21}

The expressions are obtained in symbolic environment where the following constants are named as follows: $V_{3,R} = V_R^3$, $V s_R = V_R^s$, $s_{11R} = s_{11}^R$, $s_{11E} = s_{11}^E$.

$$\lambda_1^{E_2} = 2\,V_{3,R}\,V_R\,\mathrm{Vs}_R\,\left(L_R+\sin\left(\theta\right)\right)\,\left(2\,s_{11\mathrm{R}}+t_R\right)\,\left({t_R}^3\,{t_p}^3+6\,s_{11\mathrm{R}}\,{t_R}^2\,{t_p}^3+12\,s_{11\mathrm{R}}\,t_R\,{t_p}^3+8\,s_{11\mathrm{R}}\,{t_p}^3\right)$$

$$\begin{aligned}\lambda_2^{E_2} =\ & 4\,V_{3,R}\,V_R\,\mathrm{Vs}_R\,s_{11\mathrm{E}}\,L^3\,{s_{11\mathrm{R}}}^2\cos\left(\theta\right)^3+2\,V_{3,R}\,V_R\,\mathrm{Vs}_R\,s_{11\mathrm{E}}\,L^3\,s_{11\mathrm{R}}\,t_R\cos\left(\theta\right)^3\\
&+12\,V_{3,R}\,V_R\,\mathrm{Vs}_R\,s_{11\mathrm{E}}\,L\,{s_{11\mathrm{R}}}^2\,{t_R}^2\,{t_p}^2\,\cos\left(\theta\right)\,\sin\left(\theta\right)^2+24\,V_{3,R}\,V_R\,\mathrm{Vs}_R\,s_{11\mathrm{E}}\,L\,{s_{11\mathrm{R}}}^2\,{t_R}^2\,{t_p}^2\,\cos\left(\theta\right)\\
&+24\,V_{3,R}\,V_R\,\mathrm{Vs}_R\,s_{11\mathrm{E}}\,L\,{s_{11\mathrm{R}}}^2\,t_R\,{t_p}^2\,\cos\left(\theta\right)\,\sin\left(\theta\right)^2+48\,V_{3,R}\,V_R\,\mathrm{Vs}_R\,s_{11\mathrm{E}}\,L\,{s_{11\mathrm{R}}}^2\,t_R\,{t_p}^2\,\cos\left(\theta\right)\\
&+16\,V_{3,R}\,V_R\,\mathrm{Vs}_R\,s_{11\mathrm{E}}\,L\,{s_{11\mathrm{R}}}^2\,{t_p}^2\,\cos\left(\theta\right)\,\sin\left(\theta\right)^2+32\,V_{3,R}\,V_R\,\mathrm{Vs}_R\,s_{11\mathrm{E}}\,L\,{s_{11\mathrm{R}}}^2\,{t_p}^2\,\cos\left(\theta\right)\\
&+2\,V_{3,R}\,V_R\,\mathrm{Vs}_R\,s_{11\mathrm{E}}\,L\,s_{11\mathrm{R}}\,{t_R}^3\,{t_p}^2\,\cos\left(\theta\right)\,\sin\left(\theta\right)^2+4\,V_{3,R}\,V_R\,\mathrm{Vs}_R\,s_{11\mathrm{E}}\,L\,s_{11\mathrm{R}}\,{t_R}^3\,{t_p}^2\,\cos\left(\theta\right)\end{aligned}$$

$$\begin{aligned}\lambda_3^{E_2} =\ & 16\,\mathrm{Vs}_R\,d_{31}\,{s_{11\mathrm{R}}}^2\,{t_p}^2-16\,V_{3,R}\,\mathrm{Vs}_R\,d_{31}\,{s_{11\mathrm{R}}}^2\,{t_p}^2+24\,\mathrm{Vs}_R\,d_{31}\,{s_{11\mathrm{R}}}^2\,t_R\,{t_p}^2+2\,\mathrm{Vs}_R\,d_{31}\,s_{11\mathrm{R}}\,{t_R}^3\,{t_p}^2\\
&+12\,\mathrm{Vs}_R\,d_{31}\,{s_{11\mathrm{R}}}^2\,{t_R}^2\,{t_p}^2-24\,V_{3,R}\,\mathrm{Vs}_R\,d_{31}\,{s_{11\mathrm{R}}}^2\,t_R\,{t_p}^2-2\,V_{3,R}\,\mathrm{Vs}_R\,d_{31}\,s_{11\mathrm{R}}\,{t_R}^3\,{t_p}^2\\
&-12\,V_{3,R}\,\mathrm{Vs}_R\,d_{31}\,{s_{11\mathrm{R}}}^2\,{t_R}^2\,{t_p}^2+16\,V_{3,R}\,V_R\,d_{31}\,{s_{11\mathrm{R}}}^2\,{t_p}^2\,\sin\left(\theta\right)-16\,V_{3,R}\,V_R\,\mathrm{Vs}_R\,d_{31}\,{s_{11\mathrm{R}}}^2\,{t_p}^2\,\sin\left(\theta\right)\\
&+24\,V_{3,R}\,V_R\,d_{31}\,{s_{11\mathrm{R}}}^2\,t_R\,{t_p}^2\,\sin\left(\theta\right)+2\,V_{3,R}\,V_R\,d_{31}\,s_{11\mathrm{R}}\,{t_R}^3\,{t_p}^2\,\sin\left(\theta\right)+12\,V_{3,R}\,V_R\,d_{31}\,{s_{11\mathrm{R}}}^2\,{t_R}^2\,{t_p}^2\,\sin\left(\theta\right)\\
&-6\,L\,V_{3,R}\,V_R\,d_{31}\,{s_{11\mathrm{R}}}^2\,t_p\,\cos\left(\theta\right)-12\,V_{3,R}\,V_R\,\mathrm{Vs}_R\,d_{31}\,{s_{11\mathrm{R}}}^2\,{t_R}^2\,{t_p}^2\,\sin\left(\theta\right)-6\,L\,V_{3,R}\,V_R\,\mathrm{Vs}_R\,d_{31}\,{s_{11\mathrm{R}}}^2\,t_p\,\cos\left(\theta\right)\\
&-3\,L\,V_{3,R}\,V_R\,d_{31}\,s_{11\mathrm{R}}\,{t_R}^2\,t_p\,\cos\left(\theta\right)-6\,L\,V_{3,R}\,V_R\,d_{31}\,{s_{11\mathrm{R}}}^2\,t_R\,t_p\,\cos\left(\theta\right)-24\,V_{3,R}\,V_R\,\mathrm{Vs}_R\,d_{31}\,{s_{11\mathrm{R}}}^2\,t_R\,{t_p}^2\,\sin\left(\theta\right)\\
&-2\,V_{3,R}\,V_R\,\mathrm{Vs}_R\,d_{31}\,s_{11\mathrm{R}}\,{t_R}^3\,{t_p}^2\,\sin\left(\theta\right)-3\,L\,V_{3,R}\,V_R\,d_{31}\,s_{11\mathrm{R}}\,t_R\,t_p\,\cos\left(\theta\right)-3\,L\,V_{3,R}\,V_R\,\mathrm{Vs}_R\,d_{31}\,s_{11\mathrm{R}}\,t_R\,t_p\,\cos\left(\theta\right)\\
&-3\,L\,V_{3,R}\,V_R\,\mathrm{Vs}_R\,d_{31}\,s_{11\mathrm{R}}\,{t_R}^2\,t_p\,\cos\left(\theta\right)-6\,L\,V_{3,R}\,V_R\,\mathrm{Vs}_R\,d_{31}\,{s_{11\mathrm{R}}}^2\,t_R\,t_p\,\cos\left(\theta\right)\end{aligned}$$

$$\begin{aligned}\beta_1^{\nu_{21}} =\ & -V_{3,R}\,V_R\,\left(L_R+\sin\left(\theta\right)\right)\,\left(-2\,\mathrm{Vs}_R\,s_{11\mathrm{E}}\,\sin\left(\theta\right)\,L^3\,t_R\cos\left(\theta\right)^2-4\,\mathrm{Vs}_R\,s_{11\mathrm{E}}\,s_{11\mathrm{R}}\,\sin\left(\theta\right)\,L^3\cos\left(\theta\right)^2\right.\\
&+2\,\mathrm{Vs}_R\,s_{11\mathrm{E}}\,\sin\left(\theta\right)\,L\,{t_R}^3\,{t_p}^2\cos\left(\theta\right)^2+12\,\mathrm{Vs}_R\,s_{11\mathrm{E}}\,s_{11\mathrm{R}}\,\sin\left(\theta\right)\,L\,{t_R}^2\,{t_p}^2\cos\left(\theta\right)^2\\
&\left.+24\,\mathrm{Vs}_R\,s_{11\mathrm{E}}\,s_{11\mathrm{R}}\,\sin\left(\theta\right)\,L\,t_R\,{t_p}^2\cos\left(\theta\right)^2+16\,\mathrm{Vs}_R\,s_{11\mathrm{E}}\,s_{11\mathrm{R}}\,\sin\left(\theta\right)\,L\,{t_p}^2\cos\left(\theta\right)^2\right)\end{aligned}$$

$$\begin{aligned}\beta_2^{\nu_{21}} =\ & V_{3,R}\,V_R\,\left(L_R+\sin\left(\theta\right)\right)\,\left(16\,\mathrm{Vs}_R\,d_{31}\,s_{11\mathrm{R}}\,{t_p}^2\,\cos\left(\theta\right)-16\,d_{31}\,s_{11\mathrm{R}}\,{t_p}^2\,\cos\left(\theta\right)-2\,d_{31}\,{t_R}^3\,{t_p}^2\,\cos\left(\theta\right)\right.\\
&+3\,L\,d_{31}\,{t_R}^2\,t_p\,\sin\left(\theta\right)-24\,d_{31}\,s_{11\mathrm{R}}\,t_R\,{t_p}^2\,\cos\left(\theta\right)+2\,\mathrm{Vs}_R\,d_{31}\,{t_R}^3\,{t_p}^2\,\cos\left(\theta\right)-12\,d_{31}\,s_{11\mathrm{R}}\,{t_R}^2\,{t_p}^2\,\cos\left(\theta\right)\\
&+6\,L\,d_{31}\,s_{11\mathrm{R}}\,t_p\,\sin\left(\theta\right)+3\,L\,d_{31}\,t_R\,t_p\,\sin\left(\theta\right)+12\,\mathrm{Vs}_R\,d_{31}\,s_{11\mathrm{R}}\,{t_R}^2\,{t_p}^2\,\cos\left(\theta\right)+6\,L\,\mathrm{Vs}_R\,d_{31}\,s_{11\mathrm{R}}\,t_p\,\sin\left(\theta\right)\\
&+3\,L\,\mathrm{Vs}_R\,d_{31}\,t_R\,t_p\,\sin\left(\theta\right)+6\,L\,d_{31}\,s_{11\mathrm{R}}\,t_R\,t_p\,\sin\left(\theta\right)+3\,L\,\mathrm{Vs}_R\,d_{31}\,{t_R}^2\,t_p\,\sin\left(\theta\right)+24\,\mathrm{Vs}_R\,d_{31}\,s_{11\mathrm{R}}\,t_R\,{t_p}^2\,\cos\left(\theta\right)\\
&\left.+6\,L\,\mathrm{Vs}_R\,d_{31}\,s_{11\mathrm{R}}\,t_R\,t_p\,\sin\left(\theta\right)\right)\end{aligned}$$

$$\beta_3^{\nu_{21}} = \cos(\theta)\ (2\,V_{3,R}\,V_R\,\mathrm{Vs}_R\,s_{11\mathrm{E}}\,L^3\,t_R\cos(\theta)^3 + 4\,V_{3,R}\,V_R\,\mathrm{Vs}_R\,s_{11\mathrm{E}}\,s_{11\mathrm{R}}\,L^3\cos(\theta)^3$$
$$+ 2\,V_{3,R}\,V_R\,\mathrm{Vs}_R\,s_{11\mathrm{E}}\,L\,{t_R}^3\,{t_p}^2\,\cos(\theta)\,\sin(\theta)^2 + 4\,L_R\,V_{3,R}\,V_R\,\mathrm{Vs}_R\,s_{11\mathrm{E}}\,L\,{t_R}^3\,{t_p}^2\,\cos(\theta)$$
$$+ 12\,V_{3,R}\,V_R\,\mathrm{Vs}_R\,s_{11\mathrm{E}}\,s_{11\mathrm{R}}\,L\,{t_R}^2\,{t_p}^2\,\cos(\theta)\,\sin(\theta)^2 + 24\,L_R\,V_{3,R}\,V_R\,\mathrm{Vs}_R\,s_{11\mathrm{E}}\,s_{11\mathrm{R}}\,L\,{t_R}^2\,{t_p}^2\,\cos(\theta)$$
$$+ 24\,V_{3,R}\,V_R\,\mathrm{Vs}_R\,s_{11\mathrm{E}}\,s_{11\mathrm{R}}\,L\,t_R\,{t_p}^2\,\cos(\theta)\,\sin(\theta)^2 + 48\,L_R\,V_{3,R}\,V_R\,\mathrm{Vs}_R\,s_{11\mathrm{E}}\,s_{11\mathrm{R}}\,L\,t_R\,{t_p}^2\,\cos(\theta)$$
$$+ 16\,V_{3,R}\,V_R\,\mathrm{Vs}_R\,s_{11\mathrm{E}}\,s_{11\mathrm{R}}\,L\,{t_p}^2\,\cos(\theta)\,\sin(\theta)^2 + 32\,L_R\,V_{3,R}\,V_R\,\mathrm{Vs}_R\,s_{11\mathrm{E}}\,s_{11\mathrm{R}}\,L\,{t_p}^2\,\cos(\theta))$$

$$\beta_4^{\nu_{21}} = \cos(\theta)\ (2\,L_R\,\mathrm{Vs}_R\,d_{31}\,{t_R}^3\,{t_p}^2 + 16\,L_R\,\mathrm{Vs}_R\,d_{31}\,s_{11\mathrm{R}}\,{t_p}^2 - 16\,L_R\,V_{3,R}\,\mathrm{Vs}_R\,d_{31}\,s_{11\mathrm{R}}\,{t_p}^2$$
$$+ 24\,L_R\,\mathrm{Vs}_R\,d_{31}\,s_{11\mathrm{R}}\,t_R\,{t_p}^2 - 2\,L_R\,V_{3,R}\,\mathrm{Vs}_R\,d_{31}\,{t_R}^3\,{t_p}^2 + 12\,L_R\,\mathrm{Vs}_R\,d_{31}\,s_{11\mathrm{R}}\,{t_R}^2\,{t_p}^2$$
$$+ 16\,V_{3,R}\,V_R\,d_{31}\,s_{11\mathrm{R}}\,{t_p}^2\,\sin(\theta) + 2\,V_{3,R}\,V_R\,d_{31}\,{t_R}^3\,{t_p}^2\,\sin(\theta) + 24\,V_{3,R}\,V_R\,d_{31}\,s_{11\mathrm{R}}\,t_R\,{t_p}^2\,\sin(\theta)$$
$$- 2\,V_{3,R}\,V_R\,\mathrm{Vs}_R\,d_{31}\,{t_R}^3\,{t_p}^2\,\sin(\theta) + 12\,V_{3,R}\,V_R\,d_{31}\,s_{11\mathrm{R}}\,{t_R}^2\,{t_p}^2\,\sin(\theta) - 24\,L_R\,V_{3,R}\,\mathrm{Vs}_R\,d_{31}\,s_{11\mathrm{R}}\,t_R\,{t_p}^2$$
$$+ 6\,L\,V_{3,R}\,V_R\,d_{31}\,s_{11\mathrm{R}}\,t_p\,\cos(\theta) + 3\,L\,V_{3,R}\,V_R\,d_{31}\,t_R\,t_p\,\cos(\theta) - 12\,L_R\,V_{3,R}\,\mathrm{Vs}_R\,d_{31}\,s_{11\mathrm{R}}\,{t_R}^2\,{t_p}^2$$
$$+ 3\,L\,V_{3,R}\,V_R\,d_{31}\,{t_R}^2\,t_p\,\cos(\theta) - 16\,V_{3,R}\,V_R\,\mathrm{Vs}_R\,d_{31}\,s_{11\mathrm{R}}\,{t_p}^2\,\sin(\theta) + 3\,L\,V_{3,R}\,V_R\,\mathrm{Vs}_R\,d_{31}\,{t_R}^2\,t_p\,\cos(\theta)$$
$$- 24\,V_{3,R}\,V_R\,\mathrm{Vs}_R\,d_{31}\,s_{11\mathrm{R}}\,t_R\,{t_p}^2\,\sin(\theta) - 12\,V_{3,R}\,V_R\,\mathrm{Vs}_R\,d_{31}\,s_{11\mathrm{R}}\,{t_R}^2\,{t_p}^2\,\sin(\theta)$$
$$+ 6\,L\,V_{3,R}\,V_R\,\mathrm{Vs}_R\,d_{31}\,s_{11\mathrm{R}}\,t_p\,\cos(\theta) + 3\,L\,V_{3,R}\,V_R\,\mathrm{Vs}_R\,d_{31}\,t_R\,t_p\,\cos(\theta) + 6\,L\,V_{3,R}\,V_R\,d_{31}\,s_{11\mathrm{R}}\,t_R\,t_p\,\cos(\theta)$$
$$+ 6\,L\,V_{3,R}\,V_R\,\mathrm{Vs}_R\,d_{31}\,s_{11\mathrm{R}}\,t_R\,t_p\,\cos(\theta))$$

S8. Coefficients of G_{12}

The expressions are obtained in symbolic environment where the following constants are named as follows: $V_{3,R} = V_R^3$, $V s_R = V_R^s$, $s_{11R} = s_{11}^R$, $s_{11E} = s_{11}^E$.

$$\lambda_1^{G_{12}} = 2\,V_{3,R}\,V_R\,(L_R + \sin(\theta))\,(2\,s_{11\mathrm{R}} + t_R)\,\left({t_R}^3\,{t_p}^3 + 6\,s_{11\mathrm{R}}\,{t_R}^2\,{t_p}^3 + 12\,s_{11\mathrm{R}}\,t_R\,{t_p}^3 + 8\,s_{11\mathrm{R}}\,{t_p}^3\right)$$

$$
\begin{aligned}
\lambda_2^{G_{12}} &= 8\,V_{3,R}\,V_R\,s_{11\mathrm{E}}\,L^3\,{L_R}^3\,{s_{11\mathrm{R}}}^2\,\cos(\theta) + 4\,V_{3,R}\,V_R\,s_{11\mathrm{E}}\,L^3\,{L_R}^3\,s_{11\mathrm{R}}\,t_R\,\cos(\theta) \\
&+ 12\,V_{3,R}\,V_R\,s_{11\mathrm{E}}\,L\,{L_R}^2\,{s_{11\mathrm{R}}}^2\,{t_R}^2\,{t_p}^2\,\sin(\theta) + 24\,V_{3,R}\,V_R\,s_{11\mathrm{E}}\,L\,{L_R}^2\,{s_{11\mathrm{R}}}^2\,t_R\,{t_p}^2\,\sin(\theta) \\
&+ 16\,V_{3,R}\,V_R\,s_{11\mathrm{E}}\,L\,{L_R}^2\,{s_{11\mathrm{R}}}^2\,{t_p}^2\,\sin(\theta) + 2\,V_{3,R}\,V_R\,s_{11\mathrm{E}}\,L\,{L_R}^2\,s_{11\mathrm{R}}\,{t_R}^3\,{t_p}^2\,\sin(\theta) \\
&+ 12\,V_{3,R}\,V_R\,s_{11\mathrm{E}}\,L\,L_R\,{s_{11\mathrm{R}}}^2\,{t_R}^2\,{t_p}^2\cos(\theta)^2 + 12\,V_{3,R}\,V_R\,s_{11\mathrm{E}}\,L\,L_R\,{s_{11\mathrm{R}}}^2\,{t_R}^2\,{t_p}^2\,\cos(\theta)\,\sin(\theta) \\
&+ 24\,V_{3,R}\,V_R\,s_{11\mathrm{E}}\,L\,L_R\,{s_{11\mathrm{R}}}^2\,{t_R}^2\,{t_p}^2\sin(\theta)^2 + 24\,V_{3,R}\,V_R\,s_{11\mathrm{E}}\,L\,L_R\,{s_{11\mathrm{R}}}^2\,t_R\,{t_p}^2\cos(\theta)^2 \\
&+ 24\,V_{3,R}\,V_R\,s_{11\mathrm{E}}\,L\,L_R\,{s_{11\mathrm{R}}}^2\,t_R\,{t_p}^2\,\cos(\theta)\,\sin(\theta) + 48\,V_{3,R}\,V_R\,s_{11\mathrm{E}}\,L\,L_R\,{s_{11\mathrm{R}}}^2\,t_R\,{t_p}^2\sin(\theta)^2 \\
&+ 16\,V_{3,R}\,V_R\,s_{11\mathrm{E}}\,L\,L_R\,{s_{11\mathrm{R}}}^2\,{t_p}^2\cos(\theta)^2 + 16\,V_{3,R}\,V_R\,s_{11\mathrm{E}}\,L\,L_R\,{s_{11\mathrm{R}}}^2\,{t_p}^2\,\cos(\theta)\,\sin(\theta) \\
&+ 32\,V_{3,R}\,V_R\,s_{11\mathrm{E}}\,L\,L_R\,{s_{11\mathrm{R}}}^2\,{t_p}^2\sin(\theta)^2 + 2\,V_{3,R}\,V_R\,s_{11\mathrm{E}}\,L\,L_R\,s_{11\mathrm{R}}\,{t_R}^3\,{t_p}^2\cos(\theta)^2 \\
&+ 2\,V_{3,R}\,V_R\,s_{11\mathrm{E}}\,L\,L_R\,s_{11\mathrm{R}}\,{t_R}^3\,{t_p}^2\,\cos(\theta)\,\sin(\theta) + 4\,V_{3,R}\,V_R\,s_{11\mathrm{E}}\,L\,L_R\,s_{11\mathrm{R}}\,{t_R}^3\,{t_p}^2\sin(\theta)^2 \\
&+ 12\,V_{3,R}\,V_R\,s_{11\mathrm{E}}\,L\,{s_{11\mathrm{R}}}^2\,{t_R}^2\,{t_p}^2\cos(\theta)^3 + 12\,V_{3,R}\,V_R\,s_{11\mathrm{E}}\,L\,{s_{11\mathrm{R}}}^2\,{t_R}^2\,{t_p}^2\cos(\theta)^2\,\sin(\theta) \\
&+ 12\,V_{3,R}\,V_R\,s_{11\mathrm{E}}\,L\,{s_{11\mathrm{R}}}^2\,{t_R}^2\,{t_p}^2\,\cos(\theta)\,\sin(\theta)^2 + 12\,V_{3,R}\,V_R\,s_{11\mathrm{E}}\,L\,{s_{11\mathrm{R}}}^2\,{t_R}^2\,{t_p}^2\sin(\theta)^3 \\
&+ 24\,V_{3,R}\,V_R\,s_{11\mathrm{E}}\,L\,{s_{11\mathrm{R}}}^2\,t_R\,{t_p}^2\cos(\theta)^3 + 24\,V_{3,R}\,V_R\,s_{11\mathrm{E}}\,L\,{s_{11\mathrm{R}}}^2\,t_R\,{t_p}^2\cos(\theta)^2\,\sin(\theta) \\
&+ 24\,V_{3,R}\,V_R\,s_{11\mathrm{E}}\,L\,{s_{11\mathrm{R}}}^2\,t_R\,{t_p}^2\,\cos(\theta)\,\sin(\theta)^2 + 24\,V_{3,R}\,V_R\,s_{11\mathrm{E}}\,L\,{s_{11\mathrm{R}}}^2\,t_R\,{t_p}^2\sin(\theta)^3 \\
&+ 16\,V_{3,R}\,V_R\,s_{11\mathrm{E}}\,L\,{s_{11\mathrm{R}}}^2\,{t_p}^2\cos(\theta)^3 + 16\,V_{3,R}\,V_R\,s_{11\mathrm{E}}\,L\,{s_{11\mathrm{R}}}^2\,{t_p}^2\cos(\theta)^2\,\sin(\theta) \\
&+ 16\,V_{3,R}\,V_R\,s_{11\mathrm{E}}\,L\,{s_{11\mathrm{R}}}^2\,{t_p}^2\,\cos(\theta)\,\sin(\theta)^2 + 16\,V_{3,R}\,V_R\,s_{11\mathrm{E}}\,L\,{s_{11\mathrm{R}}}^2\,{t_p}^2\sin(\theta)^3 \\
&+ 2\,V_{3,R}\,V_R\,s_{11\mathrm{E}}\,L\,s_{11\mathrm{R}}\,{t_R}^3\,{t_p}^2\cos(\theta)^3 + 2\,V_{3,R}\,V_R\,s_{11\mathrm{E}}\,L\,s_{11\mathrm{R}}\,{t_R}^3\,{t_p}^2\cos(\theta)^2\,\sin(\theta) \\
&+ 2\,V_{3,R}\,V_R\,s_{11\mathrm{E}}\,L\,s_{11\mathrm{R}}\,{t_R}^3\,{t_p}^2\,\cos(\theta)\,\sin(\theta)^2 + 2\,V_{3,R}\,V_R\,s_{11\mathrm{E}}\,L\,s_{11\mathrm{R}}\,{t_R}^3\,{t_p}^2\sin(\theta)^3
\end{aligned}
$$

$$
\begin{aligned}
\lambda_3^{G_{12}} &= -6\,L\,{L_R}^2\,d_{31}\,{s_{11\mathrm{R}}}^2\,t_p - 3\,L\,{L_R}^2\,d_{31}\,s_{11\mathrm{R}}\,t_R\,t_p - 6\,L\,{L_R}^2\,V_{3,R}\,d_{31}\,{s_{11\mathrm{R}}}^2\,t_p - 3\,L\,{L_R}^2\,d_{31}\,s_{11\mathrm{R}}\,{t_R}^2\,t_p \\
&- 6\,L\,{L_R}^2\,d_{31}\,{s_{11\mathrm{R}}}^2\,t_R\,t_p - 3\,L\,{L_R}^2\,V_{3,R}\,d_{31}\,s_{11\mathrm{R}}\,t_R\,t_p - 3\,L\,{L_R}^2\,V_{3,R}\,d_{31}\,s_{11\mathrm{R}}\,{t_R}^2\,t_p \\
&- 6\,L\,{L_R}^2\,V_{3,R}\,d_{31}\,{s_{11\mathrm{R}}}^2\,t_R\,t_p
\end{aligned}
$$

Data availability

All data sets used to generate the results are available in the main paper and the supplementary material. Further details could be obtained from the corresponding author upon reasonable request.

Author contributions

TM and SN conceived the idea. SM carried out the analyses under the supervision of TM and SN. The final manuscript was written and reviewed by all the authors.

Acknowledgments

TM and SN acknowledge the Initiation grant received from the University of Southampton.

Competing interests

The authors declare no competing interests.
