# Supplementary material for: Active heterogeneous mode coupling in bi-level multi-physically architected metamaterials for temporal, on-demand and tunable programming
Source: Commun Eng. 2025 Jun 7;4:103. doi: 10.1038/s44172-025-00420-7 (PMC12145453; doi:10.1038/s44172-025-00420-7)
Supplement: Supplementary file 2 — Description of Additional Supplementary Files [file 44172_2025_420_MOESM2_ESM.pdf]

Description of Additional Supplementary Files

File name: Supplementary Video 1

Description: Robotic locomotion 3D

File name: Supplementary Video 2

Description: Robotic locomotion 2D
